# MicroRNA Signatures in Dental Pulp Stem Cells Following Nicotine Exposure

**DOI:** 10.3390/dj13080338

**Published:** 2025-07-23

**Authors:** David Vang, Leyla Tahrani Hardin, Nabil Abid, Der Thor, Nan Xiao

**Affiliations:** 1 Department of Biomedical Sciences, Arthur A. Dugoni School of Dentistry, University of the Pacific, San Francisco, CA 94103, USA; 2 Laboratory of Transmissible Diseases and Biological Active Substances LR99ES27, Faculty of Pharmacy of Monastir, University of Monastir, Monastir 5000, Tunisia

**Keywords:** nicotine, dental pulp stem cells, miRNAs, cell signaling

## Abstract

**Background and Objectives:** Nicotine is the most well-studied toxic substance in cigarette smoke and e-cigarette vape. However, smoke and vape are composed of other components that have a negative impact on health. The objective of this study is to investigate whether nicotine has a distinctive impact on molecular mechanisms in stem cells. **Methods:** The cellular impact of nicotine on the regenerative capacity of human dental pulp stem cells (DPSCs) and the microRNA (miRNA) profile was examined. Bioinformatic analysis was performed to identify miRNA-regulated cellular pathways associated with nicotine exposure. These pathways were then compared to those induced by cigarette smoke condensate (CSC). **Results:** Prolonged exposure to nicotine significantly impaired the regeneration of DPSCs and changed the expression of miRNAs. Nicotine upregulated the expression of hsa-miR-7977, hsa-miR-3178, and hsa-miR-10400-5p compared to vehicle control. Interestingly, nicotine did not change the expression of hsa-miR-29b-3p, hsa-miR-199b-5p, hsa-miR-26b-5p, or hsa-miR-26a-5p compared to the control. However, the expressions of these miRNAs were significantly altered when compared to CSC treatment. Further analysis revealed that nicotine was distinctively associated with certain miRNA-targeted pathways including apoptosis, ErbB, MAPK signaling, PI3K-Akt, TGF-b signaling, and Wnt signaling. **Conclusions:** Our work provides evidence on the distinctive miRNA signature induced by nicotine. The information will be important for identifying the unique molecular pathways downstream of nicotine from smoking and vaping in different individuals, providing a new direction for personalized disease prevention, prognosis, and treatment.

## 1. Introduction

Despite cumulative evidence demonstrating that cigarette smoking is associated with negative impacts on health, tobacco use continues to cause around six million deaths across the world each year. The prevalence of current tobacco smoking is strikingly different globally, with the highest projected prevalence in Europe and north Asia in 2025 [1].

Nicotine is the most common and well-studied toxic substance in cigarette smoke. According to tests on 23 popular brands on the market [2], one commercial cigarette contains 6.17 to 28.86 mg of nicotine, while the amount of nicotine in electronic cigarettes (e-cigarettes) varies between 0.5 and 15.4 mg [3]. E-smoking typically involves various delivery systems and generates a higher volume of smoke or vape, which increases the risk of the environmental hazards to people exposed to the vape [4].

Both the water-soluble cigarette smoke extract (CSE) collected from the smoke gas and the cigarette smoke condensate (CSC) collected from the tar after combustion [5] contain nicotine. It is a major component in CSC, followed by the nicotine derivative nicotyrine and trace amounts of other chemicals, including ester, ethyl chloride, and phenol. However, the amount of nicotine in CSE was reported to be much lower [6].

In addition to its addictive properties, nicotine has been found to increase risks of cardiovascular diseases, cancers, and genetic diseases of the fetus [7]. Research consistently demonstrates that nicotine promotes cancer stem cell self-renewal, epithelial–mesenchymal transition, and cancer cell migration, as well as tumor formation [8]. However, the effects of nicotine on mesenchymal stem cells (MSCs) remain to be further investigated. The dose of nicotine and the origin of the targeted cells play an important role in determining the effects. For example, nicotine did not alter the behavior of human alveolar bone marrow-derived mesenchymal stem cells (BMMSCs) at doses between 1 and 100 μM. In contrast, at higher doses of 1–2 mM, nicotine increased cell proliferation but inhibited osteogenic differentiation, with cell viability significantly reduced at 5 mM [9]. Nicotine also inhibited the proliferation of human umbilical cord MSCs at concentrations starting at 0.5 mg/mL (3.08 mM) and increased apoptosis at 1–1.5 mg/mL [10]. Conversely, a dose-dependent reduction in both the proliferation and osteogenic differentiation of human periodontal ligament stem cells (PDLSCs) was reported starting at a low concentration of 1 μM nicotine [11]. Human embryonic lung fibroblasts WI38 cells exhibited higher sensitivity to nicotine, with a 24-h exposure to as little as 1 nM nicotine reducing the expression of adipogenic marker PPARγ in a dose-dependent manner [12]. However, rat-derived BMMSCs displayed greater resistance to nicotine-induced damage. Nicotine did not impair their viability at the dose of 25–100 μM but reduced chondrogenic differentiation at 25 μM and above [13]. Further study is warranted to evaluate the tissue-specific effect of nicotine among various adult stem cells.

There is growing interest in MSCs originating from the human dental pulp due to their potential in regenerative medicine. Dental pulp stem cells (DPSCs) are often harvested from the third molars (wisdom teeth), which are generally extracted to prevent recurrent infection and reduce overcrowding of the dentition. They share many characteristics with BMMSCs but can be collected through a simpler procedure with fewer post-operative complications [14], making them especially suitable for preclinical research and clinical treatment. Intravenous injection of DPSCs attenuated inflammation and neurodegeneration in an animal model [15]. A clinical trial showed that autografts of DPSCs significantly improved periodontal bone regeneration in patients [16]. There are several ongoing clinical trials registered on the National Institute of Health ClinicalTrials website that are exploring the application of DPSCs in treating neurodegeneration post ischemic stroke, repairing alveolar cleft, and promoting bone regeneration in periodontal disease. Therefore, we selected DPSCs as the stem cell model in the current study.

Our recent study revealed that cigarette smoke condensate (CSC) significantly inhibited the survival, migration, and differentiation of human DPSCs [17]. CSC altered the microRNA (miRNA) expression profile, showing significant associations with the cell cycle and p53 pathways [18]. MiRNAs are small non-coding RNAs that regulate gene expression after transcription, primarily through inhibition of translation and degradation of mRNA. Recent studies highlighted the crucial roles of miRNAs in controlling the proliferation and differentiation of both embryonic and tissue-specific stem cells [19]. In this study, we explored whether nicotine has an impact on the regenerative capacity of DPSCs and investigated its unique molecular mechanisms through miRNA-associated pathways.

## 2. Materials and Methods

### 2.1. Cell Culture and Treatment

Human DPSCs were purchased from Lonza (Cat# PT-5025). The cells were isolated from a third molar extracted from a deidentified male donor, aged 16. The use of the cells in this study was exempted by the Internal Review Board (IRB) of the University of the Pacific. Ethics, Consent to Participate, and Consent to Publish declarations were not applicable.

DPSCs were cultured in α-MEM containing 10% fetal bovine serum, antibiotics, and glutamate. Cells were exposed to vehicle control (ethanol) or nicotine from 100 nM to 1 mM (Millipore Sigma, MD) over a 6-week period. The culture medium, including the respective treatments, was refreshed twice a week. Cells were split and passaged weekly during the 6-week exposure. Cell counts were performed at each passage, and 1 × 10^6^/mL cells were reseeded at each passage. Passages from 2 to 8 were used in the experiments.

To examine the impact of nicotine on cell survival by the end of the 6-week exposure, DPSCs were trypsinized and reseeded at 2000 cells/well in 6-well plates. Upon seeding, cells were further treated with 100 nM–1 mM nicotine one more time. Cells were left to grow for 10 days without any other intervention. After this period, surviving cells were fixed and stained with crystal violet. Images were acquired and quantified using Image J (version 1.54).

### 2.2. Wound Healing Assay

After the 6-week treatment period, DPSCs were seeded at 1.5 × 10^5^ cells/well in 12-well plates. Once confluence was reached, cells were serum-starved overnight. Scratches were created with a sterile pipette tip through the monolayer. The detached cells and debris were removed by washing with phosphate-buffered saline (PBS). Images were taken at 0, 24, and 48 h after the scratch was created and quantified using Image J.

### 2.3. Cell Differentiation

DPSCs were cultured in the special medium to induce differentiation. The osteo-differentiation medium was supplemented with 10 mM β-glycerophosphate, 10 nM dexamethasone, and 1.8 mM KH_2_PO_4_. The adipo-differentiation medium was supplemented with 0.5 mM isobutylmethylxanthin, 60 μM indomethacin, 0.5 μM hydrocortisone, and 10 μg/mL insulin for up to 4 weeks. Cell lysate was harvested for alkaline phosphatase (ALP) activity measured spectrophotometrically at 405 nm (BioAssay Systems, Hayward, CA, USA) or for quantitative PCR.

### 2.4. Quantitative PCR

A Qiagen RNeasy isolation kit (Valencia, CA, USA) was used to extract total RNA from the treated DPSCs. A high-capacity cDNA reverse transcription kit (Thermo Fisher Scientific, Waltham, MA, USA) was used for cDNA synthesis. Quantitative PCR was performed using the SYBR Green Mater Mix (Thermo Fisher Scientific) in a 10 μL reaction volume following thermocycling conditions at 95 °C for 10 min, 40 cycles of 95 °C for 15 s, and 60 °C for 1 min. Relative gene expression levels were calculated using the 2^(−ΔΔCt) methods using β-actin as the reference gene for normalization. The primer sequences are shown in Supplementary Table S1.

### 2.5. MicroRNA Array

After the 6-week treatment period with 1 mM nicotine or vehicle control, total RNA and miRNAs were harvested from the DPSC lysate using the miRNeasy kit (Qiagen). Approximately 1 μg of total RNA was used for a strand-labeling reaction prior to hybridization on a µParaflo microfluidic chip (#MRA-1001, performed by LC Sciences, Houston, TX, USA) following methods previously described [18]. The chip contained 2632 probes targeting unique mature human miRNAs designed on the latest version of miRBase (ver. 22).

### 2.6. Bioinformatics Analysis

Analysis on miRNAs that were significantly upregulated or downregulated in DPSCs after treatment with 1 mM nicotine for 6 weeks (*p* < 0.05 and signal > 500) were performed using miRTargetLink2.0 and miRTarBase databases, TargetScan and miRDB, as previously described [18]. Briefly, each miRNA was subjected to pathway analysis using the miRNA Pathway Dictionary miRPathDB 2.0 and the EVmiRNA (extracellular vesicle miRNA) expression databases. Shared pathways affected by both sets of miRNAs were identified according to the KEGG gene-based network using the miR + Pathway database. The interaction of pathway networks was mapped using the PathIN tool [20] and cytoscape software 3.8.2 [21].

### 2.7. Data Analysis

Statistical analyses were performed using GraphPad Prism (ver. 10). Student’s *t*-test and one-way ANOVA were applied to determine statistical significance, with *p* < 0.05 considered significant. For bioinformatic analysis, the normality distribution of data was evaluated using the Shapiro–Wilk test, and the homogeneity of variances was determined using Levene’s test. The *p*-values were adjusted using the Bonferroni correction method [22].

## 3. Results

### 3.1. Nicotine Impaired Regeneration of DPSCs

**Figure 1:**
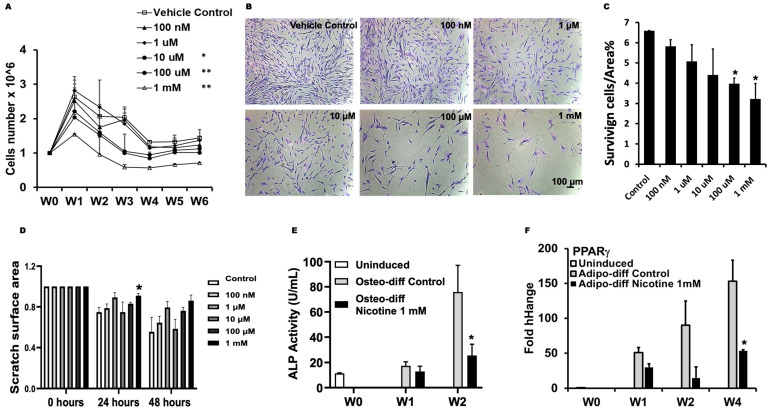
Prolonged exposure to nicotine impaired DPSC regenerative capacity. (**A**) DPSCs were exposed to vehicle control or 100 nM to 1 mM of nicotine for 6 weeks. Cells were passaged weekly at 1 × 10^6^/mL. Total cell numbers were compared at each passage. One-way ANOVA, 1 mM, 100 μM, and 10 uM significantly different than control and lower doses, * *p* < 0.05, ** *p* < 0.01, *n* = 3. (**B**) Following 6-week exposure to nicotine, each set of DPSCs was reseeded and left undisturbed. Surviving cells were stained and (**C**) quantified. (**D**) Quantification of wound scratch area at 0, 24, and 48 h. (**E**) DPSCs were cultured in osteo- or adipo-differentiation induction medium and treated with 1 mM nicotine or vehicle control for up to 4 weeks. Alkaline phosphatase (ALP) activity was measured to determine osteogenic differentiation. (**F**) qRT-PCR of adipogenic marker PPARγ. (**C**–**F**): Student’s t-test compared to control, * *p* < 0.05, *n* = 3.

Nicotine significantly inhibited DPSCs growth at 10 μM and doses above in a dose-dependent manner during the 6-week exposure (Figure 1A). To examine the impact of nicotine on cell survival by the end of the 6-week exposure, DPSCs were reseeded and treated with various doses of nicotine one more time upon seeding. A dose-dependent inhibition of DPSC survival was observed following nicotine exposure, with statistical significance at concentrations of 100 μM, and 1 mM (Figure 1B,C). Prolonged exposure to nicotine also decreased the wound healing capability of DPSCs and showed statistical significance at 1 mM nicotine (Figure 1D and Figure S1).

In addition, nicotine inhibited the differentiation of the DPSCs. At the dose of 1 mM, nicotine significantly reduced the activity of osteogenic marker alkaline phosphatase (ALP) at two weeks (Figure 1E) and the expression of the adipogenic marker Peroxisome Proliferator-Activated Receptor γ (PPARγ) at four weeks (Figure 1F).

### 3.2. Nicotine Induced Distinctive miRNA Expression

Since nicotine significantly impaired DPSC growth, survival, migration, and differentiation at 1 mM during prolonged exposure for 6 weeks, we examined its impact on miRNA expression profile at this dose. A microRNA array based on the miRBase (ver. 22) was used to compare the miRNA expression in DPSCs after 1 mM nicotine or vehicle treatment for 6 weeks. We identified nine miRNAs significantly upregulated by nicotine (hsa-miR-4497, hsa-miR-7977, hsa-miR-3178, hsa-miR-1260b, hsa-miR-10400-5p, hsa-let-7e-5p, hsa-let-7a-5p, hsa-let-7d-5p, and hsa-let-7c-5p), and nine miRNAs significantly downregulated by nicotine (hsa-miR-376c-3p, hsa-miR-377-3p, hsa-miR-222-3p, hsa-miR-130a-3p, hsa-miR-143-3p, hsa-miR-127-3p, hsa-miR-221-3p, hsa-miR-12136, and hsa-miR-22-3p9) (Table 1).

### 3.3. Analysis of miRNA Targets and Associated Pathway Induced by Nicotine

We further analyzed the downstream targets of the 18 miRNAs whose expression was significantly altered by nicotine treatment. These miRNAs also showed strong expression in the DPSCs (*p* < 0.05, signal > 500, *n* = 3).

Four databases, miRTargetLink2.0, miRTarbase, and miRDB/TargetScan, were used to identify the consensus targets of these miRNAs. TRAF3 was the target of miRNA hsa-miR-3178, which was upregulated by nicotine. Multiple targets, including AGO1, BCL2, CCND1, IGF1, DICER1, MAP4K, TGFBR1, and TGFBR3, were identified downstream of the hsa-let7 family of miRNAs, which were upregulated by nicotine as well. Many targets, including RUNX2, TRPS1, VGLL4, MEOX2, KRAS, MAPK7, KIT, PPARG, FOS, and BCL2L11, which are involved in cell growth, migration, differentiation, cellular metabolism, cell cycle, and apoptosis, were found downstream of the miRNAs downregulated by nicotine (Supplementary Table S2).

To investigate the relationship of these miRNAs and their targets in signaling pathways, we used the miRPathDB V.2 (Supplementary Table S3) and EVmiRNA (Supplementary Table S4) databases to analyze the downstream targets in associated KEGG pathways. We identified 39 consensus pathways regulated by the significantly upregulated or downregulated miRNAs in nicotine-treated DPSCs (Supplementary Table S5).

**Figure 2:**
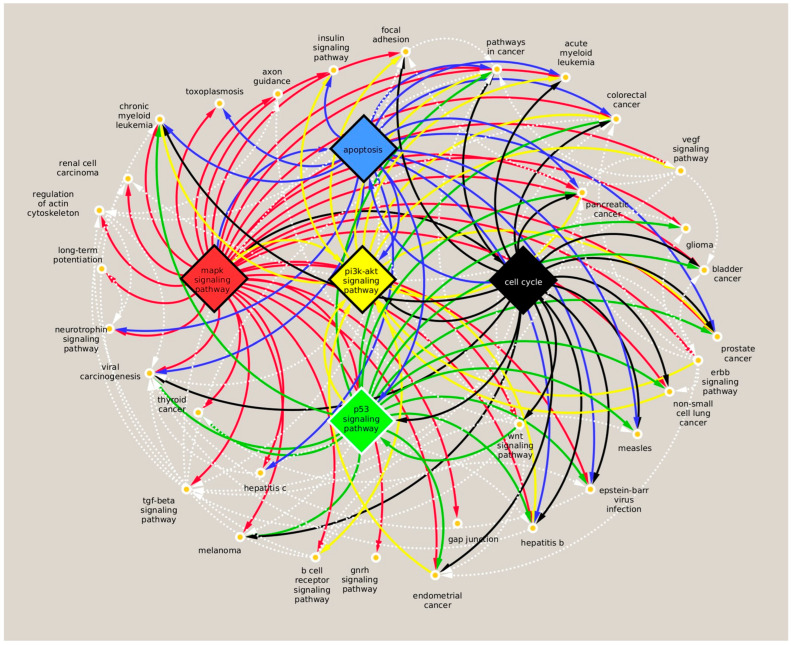
Pathway intersection. The network of signaling pathways associated with miRNAs which were significantly different in nicotine-treated DPSCs compared to controls. The diagram was generated using the PathIN tool and cytoscape. Unweighted edges refer to common genes between two pathways. The pathways showing the most abundant interactions are highlighted in different colors (red, yellow, black, blue, and green). The less abundant interactions are shown with dashed white lines.

The gene-based pathway interaction network showed that, in addition to the cell cycle and p53 signaling pathways, the apoptosis, MAPK, and PI3K-Akt pathways shared many miRNA-targeted genes that play essential roles in other signaling pathways (Figure 2). The apoptosis signaling pathway is significantly associated with upregulated let-7 family miRNAs, let-7e-5p, let-7a-5p, let-7d-5p, and let-7c-5p (red) and downregulated miRNAs, miR-376c-3p, miR-377-3p, miR-222-3p, miR-130a-3p, miR-143-3p, miR-221-3p, and miR-22-3p (blue) in DPSCs after extended exposure to nicotine (Supplementary Figure S2A). In addition, let-7e-5p, let-7a-5p, let-7c-5p, miR-130a-3p, miR-143-3p, miR-221-3p, and miR-22-3p were also associated with MAPK and PI3K-Akt signaling pathways (Supplementary Figure S2B,C) as well as the cell cycle and p53 signaling pathways (Supplementary Figure S2D,E).

### 3.4. Nicotine Induced Unique Molecular Signatures in DPSCs in Comparison to CSC

Since we previously reported that CSC induced changes in the microRNA profile of DPSCs [18], we further compared the differences in miRNA profiles between nicotine and CSC treatment in this study. Both nicotine and CSC significantly upregulated the expression of hsa-miR-7977, hsa-miR-3178, and hsa-miR-10400-5p compared to vehicle control. Nicotine showed significantly stronger induction of hsa-miR-7977 as compared to CSC (Table 2). There were no commonly downregulated miRNAs between nicotine and CSC treatment. Interestingly, nicotine upregulated the expression of hsa-miR-26a-5p, hsa-miR-26b-5p, and hsa-miR-199b-5p and downregulated hsa-miR-29b-3p compared to CSC but not to vehicle control (Table 2).

We also identified 15 identical pathways downstream of nicotine- and CSC-regulated miRNAs. The common pathways mainly involved the cell cycle, focal adhesion, hepatitis B, measles infection, p53 signaling, and cancer-related pathways (chronic myeloid leukemia, colorectal cancer, endometrial cancer, glioma, melanoma, pancreatic cancer, prostate cancer, renal cell carcinoma, and viral carcinogenesis). Nicotine alone was also associated with many other types of cancer signaling, including acute myeloid leukemia, bladder cancer, non-small-cell lung cancer, and thyroid cancer. Notably, nicotine was distinctively associated with critical pathways regulating cell metabolism and stem cell function, such as apoptosis, ErB, insulin, MAPK, neurotrophin, PI3K-Akt, TGF-beta, VEGF, and wnt signaling pathways (Figure 2).

## 4. Discussion

Our results showed that prolonged nicotine exposure significantly impaired DPSC regeneration and resulted in a distinct miRNA profile associated with several key signaling pathways crucial for stem cell regeneration.

It was reported that the plasma nicotine level reached the peak (22.5 ± 17.01 ng/mL) around 1-h post smoking of commercially available high-tar cigarettes [23]. Previous reports showed that nicotine levels in serum typically range between 4 and 72 ng/mL [24]. However, the concentration can be rapidly elevated to approximately 10–100 μM in serum and transiently reached up to 1 mM at the mucosal surface directly exposed to smoke immediately after smoking a single cigarette [25]. To mimic light and heavy smokers, we treated DPSCs with 100 nM–1 mM nicotine.

Our results showed that low doses of nicotine, specifically 100 nM and 1 μM, did not inhibit the growth of DPSCs, although DPSC growth slowed down with each passage over the 6-week experiment. The findings align with our previous report, which revealed that short-term exposure (72 h) to 100 nM–1 mM nicotine did not change the proliferation of DPSCs [17]. In contrast, prolonged exposure to nicotine for up to 6 weeks significantly impaired the growth of DPSCs starting at a concentration of 10 µM. Furthermore, exposure to a high nicotine concentration of 10 mM led to a substantial inhibition of DPSC proliferation and survival, even with short-term exposure [17]. The results suggest that while DPSCs exhibit some resistance to nicotine-induced proliferation impairment at low to moderate doses over short periods, prolonged exposure to nicotine or brief exposure to very high doses still markedly reduces DPSC growth.

Likewise, we did not observe significant impairment of DPSC migration at lower doses of nicotine for either prolonged or short exposure. However, the clonogenic survival of DPSCs was more sensitive to nicotine exposure. A dose-dependent impairment was observed during prolonged exposure, which is consistent with our previous findings for short nicotine exposure. Nicotine also inhibited the differentiation of DPSCs. These results suggest that long-term light smokers may continue to experience adverse effects of nicotine on stem cell.

Nicotine is a major component of cigarette smoke condensate (CSC). It accounts for approximately 55.8% of CSC, which also contains various other toxic components [6]. Results in this study showed that 1 mM nicotine produced a comparable level of inhibition on DPSC function as 100 μg/mL CSC, which equals approximately 350 μM nicotine [17]. These findings indicate that while nicotine alone impairs stem cell function, the presence of additional compounds in CSC may contribute distinct or synergistic effects, highlighting the need to further investigate the shared and unique mechanism in the biological process.

Because miRNAs play important roles in fine-tuning stem cell function, we investigated whether nicotine altered miRNA expression in DPSCs as well as the downstream signaling pathways associated with the miRNA profile. As we previously reported that cigarette smoke condensate (CSC) changed the microRNA profile of DPSCs [18], we also compared the differences between nicotine and CSC treatments.

Both nicotine and CSC upregulated the expression of hsa-miR-7977, hsa-miR-3178, and hsa-miR-10400-5p, with nicotine causing a more pronounced increase in hsa-miR-7977, suggesting that nicotine may be the primary component in CSC responsible for the upregulation of this miRNA in DPSCs. miR-7977 is enriched in acute myeloid leukemia cells [26]. Mimics of miR-7977 can significantly reduce Hippo-YAP signaling, inactivate contact inhibition, and promote cell proliferation in MSCs [27]. Increased expression of miR-7977 in DPSCs may be linked to resistance against the nicotine-induced reduction in proliferation in CSC or nicotine-treated DPSCs.

miR-3178 expression was significantly lower in triple-negative breast cancer (TNBC) and correlated with decreased patient survival rates. Overexpression of miR-3178, on the other hand, inhibited the malignancy of TNBC cells by targeting Notch 1 signaling [28]. Additionally, miR-3178 overexpression has been shown to reduce migration and invasion in prostate, lung, and breast cancer cells [29]. These findings align with our observation that nicotine increased miR-3178 levels and suppressed migration in dental pulp stem cells (DPSCs). Furthermore, an elevation in miR-10400-5p level in patients’ plasma was detected at 4–7 days following severe burn [30] and also in colorectal and bladder cancers [31]. However, its precise function remains to be elucidated.

Interestingly, nicotine did not change the expression level of hsa-miR-26a-5p, hsa-miR-26b-5p, and hsa-miR-199b-5p compared to the vehicle control. However, the expression of the three miRNAs was downregulated by CSC compared to either the control or nicotine. Likewise, nicotine did not change the expression level of hsa-miR-29b-3p, which was upregulated by CSC compared to either the control or nicotine. The findings indicate that non-nicotine substances in CSC are responsible for the significant changes in the four miRNAs in DPSCs.

Pathway analysis revealed that the miRNA profile induced by nicotine was linked to the cell cycle, p53 signaling, and several cancer pathways. Notably, many of these pathways are identical with our previous findings related to the significant altered miRNAs in CSC-treated DPSCs [18]. It is reasonable considering that nicotine is carcinogenic and is a major component of CSC [6]. Nicotine induced a unique miRNA profile associated with MAPK signaling and PI3K-Akt pathways. The results align with our previous findings that nicotine induces phosphorylation of AKT and MAPK, whereas CSC does not [17].

Many downstream targets of the nicotine-induced miRNAs are critical regulators of pathways that play important roles in stem cell proliferation, migration, and differentiation [32,33]. For instance, IGF1, IGF1R, and ITGB3 are associated with the PI3K-Akt, ErbB, and Wnt pathways; NRAS, KRAS, and MAP4K3 are involved in the MAPK, TGF-β, and Wnt pathways; TGFBR1 and TGFBR3 are key receptors in the TGF-β signaling pathway; TRAF3, BCL2L1, BCL2L11, and BBC3 are regulators of apoptosis.

Nicotine elevated the expression of hsa-let-7e-5p, hsa-let-7a-5p, hsa-let-7d-5p, and hsa-let-7c-5p. The hsa-let7 family miRNAs are highly conserved and expressed in higher amounts during embryogenesis. They have been reported to regulate stem cell and progenitor cell self-renewal, proliferation, quiescence, differentiation, and function as a tumor suppressor [34,35]. Patients with metastatic prostate cancer exhibited significantly decreased levels of let-7c. Moreover, let-7c derived from human BMMSCs inhibited the proliferation and migration of prostate cancer cell lines in vitro [36]. let-7c overexpression impaired osteogenic differentiation in MSCs derived from adipose tissue. Its serum level was significantly higher in postmenopausal women with osteoporosis [37]. The findings are in line with our observation that nicotine increased the expression of hsa-let-7c-5p and inhibited the proliferation, migration, and osteogenic differentiation of DPSCs.

However, let-7a, which was highly expressed in human BMMSC extracellular vesicles [38], was reported to enhance BMMSC osteogenic differentiation [39] and promote neural stem cell differentiation and growth [40]. In addition, let-7a-5p also suppressed MAPK signaling-related inflammation in endothelia [38]. let-7e-5p was enriched in umbilical cord MSC extracellular vesicle and alleviated oral mucositis by targeting TAB2 and inhibiting NF-kB signaling [41]. When neural stem cells were transfected with plasmid to overexpress let-7d, cell proliferation was impaired, while differentiation and migration were enhanced [42]. Further research is necessary to identify the role of various let-7 miRNA family members in regulating stem cell regeneration and in mediating nicotine-induced inflammatory responses in DPSC.

Other miRNAs altered by nicotine exposure have also been shown to regulate stem cell activity. For instance, miR-1260b expression was induced by the tumor necrosis factor (TNFα) in MSC derived from human gingiva tissue. Injection of miR-1260b mimic into the periodontal region of mouse molars inhibited osteoclast activity and bone loss in the animal through suppression of the activating transcription factor (ATF)-6β [43]. The report aligns with our observation that nicotine increased the expression of hsa-miR-1260b and inhibited the osteogenic differentiation of DPSCs. Similarly, nicotine decreased the expression of hsa-miR-130a-3p and inhibited the differentiation of DPSCs. Our observation was consistent with previous reports showing that overexpression of miR-130a-3p promoted neural stem cell differentiation through activation of the PI3K-Akt pathway and downregulation of Acsl4 [44], as well as enhanced osteogenic differentiation of adipose-derived MSCs through activation of the Wnt signaling pathway and downregulation of SIRT7 [45].

In contrast, nicotine downregulated the expression of multiple miRNAs, which have been reported to inhibit stem cell regeneration. For instance, miR-376c-3p mimic inhibited the osteogenic differentiation of BMMSCs by downregulation of IGF1R and Akt signaling, whereas its inhibitor promoted the process [46]. miR-377-3p overexpression suppressed the adipogenesis of BMMSCs [47]. miR-222-3p overexpression impaired the proliferation and differentiation of C2C12 myoblast cells through inhibition of the PI3K-Akt pathway [48]. Transfection of a miR-222-3p mimic increased caspase-3 expression and induced apoptosis in HL60 acute myeloid leukemia cells [49]. miR-143-3p was found enriched in inflammatory periodontal ligament stem cells (PDLSCs), suppressed PI3K-Akt signaling, but activated NF-kB signaling [50]. miR-143-3p also targeted CHK2 in the cell cycle pathway, and the repression of it induced autophagy in rat cardiac muscle cell line H9c2 cells [51]. Exosomal miR-221-3p derived from BMMSCs inhibited MAPK signaling by targeting FGF2, which further impaired the proliferation and migration of the airway smooth muscle cells [52]. miR-221-3p targeted FOXP1 and promoted angiogenesis in diabetic wounds of human umbilical vein endothelial cells [53]. In addition, overexpression of miR-22 inhibited the migration and invasion of liver epithelia cells by targeting CBL and suppressing the MAPK pathway [54]. Expression of miR-22-3p decreased in colorectal cancer cells, and mimics of the miRNA reduced the proliferation and invasion of SW480 intestine cells [55]. These miRNAs might be involved in self-recovery activity to antagonize the deleterious impact of nicotine on DPSC regeneration.

Future loss and gain of function assays utilizing miRNA mimics and inhibitors and knockdown and overexpression of the miRNA-targeted genes are warranted to further elucidate the roles of the observed key miRNAs in stem cell regeneration. However, further research is needed to refine miRNA-based therapies, addressing challenges such as the enrichment of exosomal miRNAs, optimal delivery routes, stability and efficacy of miRNA mimics and inhibitors, specificity and off-target effects, toxicity, and the clearance of exogenous miRNA reagents. Despite these challenges, ongoing advancements in these areas hold great promise for transforming experimental results into therapeutic strategies and enhancing care for smokers as well as nonsmokers.

## 5. Conclusions

Our results indicate that prolonged nicotine exposure significantly impaired DPSC regenerative capacity and led to a distinctive miRNA expression profile. Specifically, nicotine-induced miRNA changes were uniquely associated with apoptosis, ErbB, MAPK signaling, PI3K-Akt, TGF-β, and Wnt signaling pathways, distinguishing them from those induced by CSC.

## Figures and Tables

**Table 1:** List of miRNAs with statistical significance in nicotine vs. vehicle-treated DPSCs. List of miRNAs that were significantly upregulated or downregulated in DPSCs after treatment with 1 mM nicotine or vehicle control for 6 weeks (*p* < 0.05 and signal > 500, *n* = 3).

Upregulated Nicotine vs. Vehicle	Downregulated Nicotine vs. Vehicle
hsa-miR-4497	hsa-miR-376c-3p
hsa-miR-7977	hsa-miR-377-3p
hsa-miR-3178	hsa-miR-222-3p
hsa-miR-1260b	hsa-miR-130a-3p
hsa-miR-10400-5p	hsa-miR-143-3p
hsa-let-7e-5p	hsa-miR-127-3p
hsa-let-7a-5p	hsa-miR-221-3p
hsa-let-7d-5p	hsa-miR-12136
hsa-let-7c-5p	hsa-miR-22-3p

**Table 2:** List of miRNAs with statistical significance in nicotine- vs. CSC-treated DPSCs. List of miRNAs that were significantly upregulated or downregulated in DPSCs after treatment with 1 mM nicotine in comparison to that after treatment with 100 mg/mL CSC (*p* < 0.05 and signal > 500, *n* = 3).

Upregulated Nicotine vs. CSC	Downregulated Nicotine vs. CSC
hsa-miR-199b-5p	hsa-miR-10395-5p
hsa-miR-1260b	hsa-miR-222-3p
hsa-miR-7977	hsa-miR-221-3p
hsa-miR-30b-5p	hsa-miR-29b-3p
hsa-miR-26a-5p	hsa-miR-4321
hsa-miR-26b-5p	
hsa-miR-199a-3p	
hsa-miR-199a-5p	

## Data Availability

The original contributions presented in this study are included in the article/Supplementary Materials. Further inquiries can be directed to the corresponding author.

## Supplementary Materials

The following supporting information can be downloaded at https://www.mdpi.com/article/10.3390/dj13080338/s1. Supplementary Figure S1. Impact of prolonged exposure to nicotine on DPSC migration. DPSCs were treated with nicotine for 6 weeks and then seeded at 1.5 × 10^5^ cells/well and serum-starved overnight after reaching confluence. Phase-contrast pictures of the wounds were taken at 0 h, 24 h, and 48 h. Supplementary Figure S2. Pathway intersection. (A) The apoptosis pathway was significantly associated with the upregulated let-7 family miRNAs, let-7e-5p, let-7a-5p, let-7d-5p, and let-7c-5p (red) and the downregulated miRNAs, miR-376c-3p, miR-377-3p, miR-222-3p, miR-130a-3p, miR-143-3p, miR-221-3p, and miR-22-3p (blue). (B) The MAPK pathway and (C) the PI3K-AKT signaling pathway were significantly associated with the upregulated let-7 family miRNAs, let-7e-5p, let-7a-5p, and let-7c-5p (red) and the downregulated miRNAs miR-376c-3p, miR-377-3p, miR-222-3p, miR-130a-3p, miR-143-3p, miR-221-3p, and miR-22-3p (blue). (D) The cell cycle pathway was significantly associated with the upregulated miRNAs miR-1260b, let-7e-5p, let-7a-5p, and let-7c-5p (red) and the downregulated miRNAs miR-130a-3p, miR-143-3p, miR-221-3p, and miR-22-3p (blue). (E) The p53 signaling pathway was significantly associated with the upregulated let-7 family miRNAs let-7e-5p, let-7a-5p, let-7d-5p, and let-7c-5p (red) and the downregulated miRNAs miR-377-3p, miR-222-3p, miR-130a-3p, miR-143-3p, miR-221-3p, and miR-22-3p (blue). Red square: targeted by the upregulated miRNAs. Blue square: targeted by the downregulated miRNAs. The red circle with a number at the top right corner of each gene represents the total number of miRNAs regulating the gene in the miR + Pathway database. Supplementary Table S1: List of qPCR primers. Supplementary Table S2. The miRNA-targeted genes significantly induced by nicotine vs. control in DPSCs. Supplementary Table S3. Pathway analysis using miRPathDB v2.0 downstream of up- and downregulated miRNAs in nicotine-treated DPSCs vs. control. Supplementary Table S4. Pathway analysis using EVmiRNAs downstream of up- and downregulated miRNAs in nicotine-treated DPSCs vs. control. Supplementary Table S5. Consensus pathways using miRPathDB v2.0 and EVmiRNAs downstream of up- and downregulated miRNAs in nicotine-treated DPSCs vs. control.
