# Supplementary material for: MicroRNA Signatures in Dental Pulp Stem Cells Following Nicotine Exposure
Source: Dent J (Basel). 2025 Jul 23;13(8):338. doi: 10.3390/dj13080338 (PMC12385508; doi:10.3390/dj13080338)
Supplement: Supplementary file 1 [file dentistry-13-00338-s001.zip › dentistry-3670005-supplementary.pdf]

Nicotine Induces Distinctive MicroRNA Profiles versus Cigarette Smoke in Dental Pulp Stem Cells

Running title: Unique miRNA profile induced by nicotine in DPSCs

David Vang[1], Leyla Tahrani Hardin[1], Nabil Abid[2], Der Thor[1], Nan Xiao[1*]

[1] Department of Biomedical Sciences at the Arthur A. Dugoni School of Dentistry, University of the Pacific, San Francisco, CA 94103, USA

[2] Laboratory of Transmissible Diseases and Biological Active Substances LR99ES27, Faculty of Pharmacy of Monastir, University of Monastir, Monastir 5000, Tunisia

* Correspondence: nxiao@pacific.edu

Supplementary Information

Supplementary Figure S1. **Impact of prolonged exposure to nicotine on DPSC migration.** DPSCs were treated with nicotine for 6 weeks, then seeded at 1.5 x 10^5 cells/well and serum starved overnight after reaching confluence. Phase-contrast pictures of the wounds were taken at 0 hours, 24 hours, and 48 hours.

Supplementary Figure S2. Pathway intersection. (A) The apoptosis pathway significantly associated with the up-regulated let-7 family miRNAs let-7e-5p, let-7a-5p, let-7d-5p and let-7c-5p (red), and the down regulated miRNAs miR-376c-3p, miR-377-3p, miR-222-3p, miR-130a-3p, miR-143-3p, miR-221-3p and miR-22-3p (blue). **(B)** The MAPK pathway and **(C)** The PI3K-AKT signaling pathway significantly associated with the up-regulated let-7 family miRNAs let-7e-5p, let-7a-5p and let-7c-5p (red), and the down regulated miRNAs miR-376c-3p, miR-377-3p, miR-222-3p, miR-130a-3p, miR-143-3p, miR-221-3p and miR-22-3p (blue). **(D)** The cell cycle pathway way significantly associated with the up-regulated miRNAs miR-1260b, let-7e-5p, let-7a-5p and let-7c-5p (red), and thedown regulated miRNAs miR-130a-3p, miR-143-3p, miR-221-3p and miR-22-3p (blue). **(E) T**he p53 signaling pathway significantly associated with the up-regulated let-7 family miRNAs let-7e-5p, let-7a-5p, let-7d-5p and let-7c-5p (red), and the down regulated miRNAs miR-377-3p, miR-222-3p, miR-130a-3p, miR-143-3p, miR-221-3p and miR-22-3p (blue). Red square: targeted by the up-regulated miRNAs. Blue square: targeted by the down

regulated miRNAs. The red circle with number at the top right corner of each gene represents the total number of miRNAs regulating the gene in the miR + Pathway database.

Supplementary Table S1: List of qPCR primers.

Supplementary Table S2. The miRNA-target genes significantly induced by nicotine vs control in DPSCs.

Supplementary Table S3. Pathway analysis using miRPathDB v2.0 downstream of up and down regulated miRNAs in nicotine-treated DPSCs vs control.

Supplementary Table S4. Pathway analysis using EVmiRNAs downstream of up and down regulated miRNAs in nicotine-treated DPSCs vs control.

Supplementary Table S5. Consensus pathways using miRPathDB v2.0 and EVmiRNAs downstream of up and down regulated miRNAs in nicotine-treated DPSCs vs control.

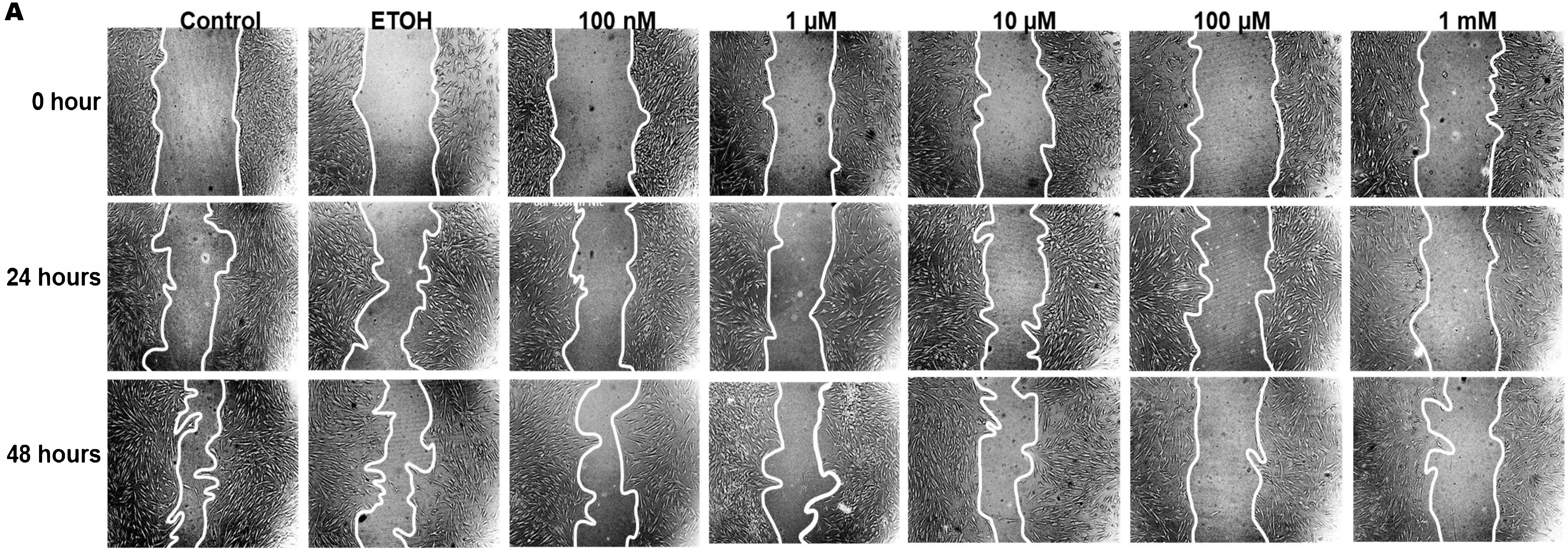

Supplementary Figure S1. Impact of prolonged exposure to nicotine on DPSC migration. DPSCs were treated with nicotine for 6 weeks, then seeded at 1.5 x 10^5/well and serum starved overnight after reaching confluence. Phase-contrast pictures of the wounds were taken at 0 hours, 24 hours, and 48 hours.

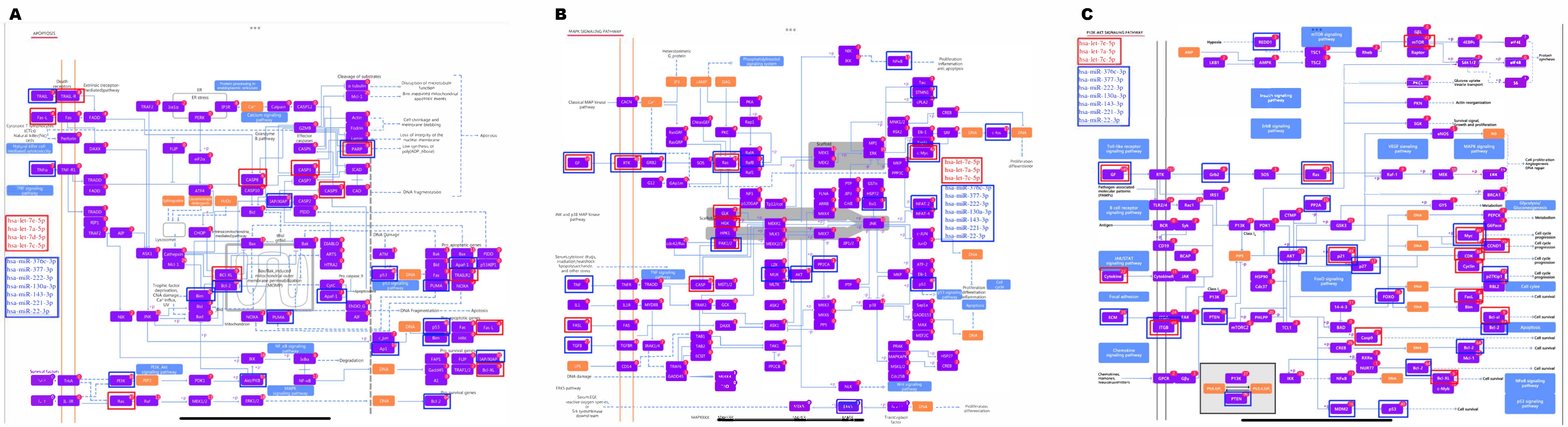

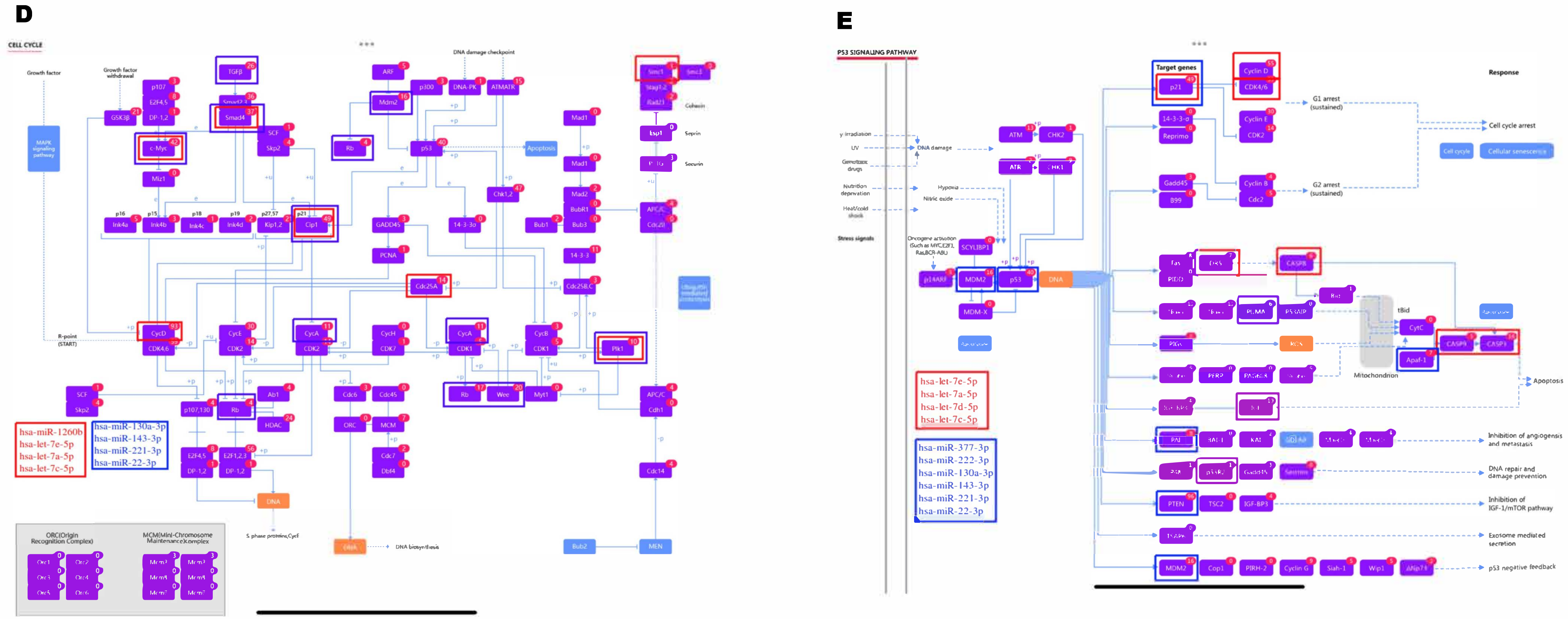

Supplementary Figure S2. Pathway intersection. (A) The apoptosis pathway significantly associated with the up-regulated let-7 family miRNAs let-7e-5p, let-7a-5p, let-7d-5p and let-7c-5p (red), and the down regulated miRNAs miR-376c-3p, miR-377-3p, miR-222-3p, miR-130a-3p, miR-143-3p, miR-221-3p and miR-22-3p (blue). **(B)** The MAPK pathway and **(C)** The PI3K-AKT signaling pathway significantly associated with the up-regulated let-7 family miRNAs let-7e-5p, let-7a-5p and let-7c-5p (red), and the down regulated miRNAs miR-376c-3p, miR-377-3p, miR-222-3p, miR-130a-3p, miR-143-3p, miR-221-3p and miR-22-3p (blue). **(D)** The cell cycle pathway way significantly associated with the up-regulated miRNAs miR-1260b, let-7e-5p, let-7a-5p and let-7c-5p (red), and the down regulated miRNAs miR-130a-3p, miR-143-3p, miR-221-3p and miR-22-3p (blue). **(E)** The p53 signaling pathway significantly associated with the up-regulated let-7 family miRNAs let-7e-5p, let-7a-5p, let-7d-5p and let-7c-5p (red), and the down regulated miRNAs miR-377-3p, miR-222-3p, miR-130a-3p, miR-143-3p, miR-221-3p and miR-22-3p (blue). Red square: targeted by the up-regulated miRNAs. Blue square: targeted by the down regulated miRNAs. The red circle with number at the top right corner of each gene represents the total number of miRNAs regulating the gene in the miR + Pathway database.

Supplementary Table S1. List of qPCR primers.		
**Gene**	**Forward primers**	**Reverse primers**
β-actin	AGAGCTACGAGCTGCCTGAC	AGCACTGTGTTGGCGTACAG
PPARγ	CTCCTATTGACCCAGAAAGC	GTAGAGCTGAGTCTTCTCAG

Supplementary Table S2. The miRNA-target genes significantlyaffected by nicotine vs control in DPSCs.

Upregulated miRNAs were highlighted in red

Downregulated miRNAs were highlighted in blue

ND: No data

	miRNA	log2 (nicotine/ control)	MiRTargetLink2.0 (up to 19/09/2023)	miRTarBase and miRDB /TargetScan (up to 19/09/2023)	Consensus
Up regulated	hsa-miR-4497	1.30	ND	ND	ND
	hsa-miR-7977	1.25	ND	AFMID, ALG9, APOBEC3F, ATG9A, C1QTNF6, CBX5, CCL22, COX18, CRTAP, CSRNP3, FAM71F2, GNG4, GREB1, HAUS5, HSPA1B, IBA57, IKZF3, LONRF2, MARVELD2, MICOS10, MPRIP, OR7D2, PCBD2, PGPEP1, PPP1R16B, RAB36, RBMS2, RNF24, SH3BP2, SHISA2, SLC25A34, SNTG1, SNX1, SNX27, SP1, SRP19, TFDP2, TPM3, TRAPPC2, TRPM6, WDR55, WIZ, YIPF4, ZNF264, ZNF451, ZNF557, ZSCAN29, ZYG11A	ND
	hsa-miR-3178	1.17	TRAF3	BVES, FXYD1, TRAF3	**TRAF3**
	hsa-miR-1260b	1.03	DKK2,SFRP1,SMAD4	GATAD2B, KIF21A, NCAPG2, PHF5A, TSC22D2, UGT8	ND
	hsa-miR-10400-5p	0.92	ND	ND	ND
	hsa-let-7e-5p	0.80	AGO1,ARID3A,AURKB,CCND1,EZH2,FASLG,HMGA2,IGF1,IGF1R,LIN28A,MMP9,MYCN,PLK1,SMC1A,TNFAIP3,TNFRSF10B,WNT1	ADIPOR2, AGO1, ANKRD46, BACH1, BEND4, BZW1, C5orf51, CALU, CBX5, CCND1, CCNT2, CDKN1A, CDV3, CLDN12, COIL, CPA4, CRK, CRY2, DHX57, DVL3, DYRK2, ERO1A, FAM104A, FIGN, FNDC3A, GPAT4, HIF1AN, HMGA1, HMGA2, IGF1, IGF1R, IGF2BP1, KATNAL1, KCTD21, KIAA0930, KLHDC8B, KREMEN1, LIMD2, LIN28A, LMLN, MBD2, MDM4, MIEF1, MLLT10, MSI2, MTUS1, MXD1, NAP1L1, NAT8L, NHLRC3, NME4, NSD1, PACS2, PAX3, PCGF3, PDE12, PDGFB, PDP2, PEX11B, PGM2L1, PLAGL2, PLXND1, POLR3D, PPP1R15B, RAB40C, RANBP2, RBFOX2, RDX, RNF44, RRM2, RUNX1T1, SCD, SLC10A7, SLC5A6, SMARCAD1, SMC1A, SMCR8, SOCS1, STX3, SYT1, TGFBR3, TNFSF9, TRIM71, TSC22D2, TXLNA, TXLNG, UGGT1, USP38, YOD1, ZBTB5, ZNF200, ZNF566, ZNF644	**AGO1,CCND1,HMGA2,IGF1,IGF1R,LIN28A,SMC1A**
	hsa-let-7a-5p	0.76	AGO1,AGO4,ARG2,AURKB,CASP3,CCND2,CCR7,CDC34,CDK6,CDKN1A,DICER1,E2F2,EGFR,EWSR1,EZH2,HAS2,HMGA1,HMGA2,HRAS,IGF2,IGF2BP1,IL6,ITGB3,KRAS,LIN28A,LIN28B,MAP4K4,MYC,NF2,NKIRAS2,NRAS,PAK1,PARP1,PKM,PRDM1,RAB40C,RAVER2,RRM2,STAT3,TGFBR3,TNFAIP3,TNFRSF10B,TRIM71,UHRF1,UHRF2,WNT1	ACER2, AGO1, ANKRD46, AP1S1, ARID3A, ARID3B, ATP2A2, BACH1, BCAT1, BEND4, BZW1, C5orf51, CBX5, CCNT2, CCR7, CDC34, CFL2, CNTRL, COIL, CPA4, DHX57, DICER1, DVL3, E2F2, E2F6, EDEM3, ERO1A, FAM104A, FNIP1, GOLGA4, GPAT4, HMGA2, IGF2BP1, ITGB3, KMT2D, KREMEN1, LIMD2, LIN28A, LIN28B, MDM4, MIB1, MRS2, MTUS1, NAP1L1, NKIRAS2, NME4, NRAS, PBX2, PDP2, PGRMC1, PLAGL2, PLEKHO1, POLR2D, PPP2R2A, PRDM1, RAB11FIP4, RANBP2, RBFOX2, RDX, RNF44, SLC20A1, SLC5A6, SMC1A, SOCS1, SURF4, SYT1, TAB2, TGFBR3, THBS1, TUSC2, TXLNG, WIPI2, YOD1, ZNF644	**AGO1,CCR7,CDC34,DICER1,E2F2,HMGA2,IGF2BP1,ITGB3,LIN28A,LIN28B,NKIRAS2,NRAS,PRDM1,TGFBR3**
	hsa-let-7d-5p	0.63	AGO1,COL3A1,CRYGEP,DICER1,HMGA2,IL13,SLC11A2,TNFRSF10B	AGO1, ANKRD46, AP1S1, ARID3A, ARID3B, BACH1, BZW1, C19orf47, C5orf51, CALU, CBX5, CCNT2, CLDN12, CPA4, CRY2, DHX57, DICER1, DVL3, EDEM3, EIF4G2, EPHA4, ERO1A, ESPL1, FAM104A, FIGN, FNDC3A, GPAT4, HMGA1, IGF2BP1, KATNAL1, KIAA0930, KMT2D, LBR, LRIG3, MBD2, MDM4, MSI2, MTUS1, MXD1, NAA30, NAP1L1, NEMP1, NR6A1, PBX2, PCGF3, PDE12, PDP2, PEG10, PGM2L1, PLD3, PLEKHO1, POLR2D, PPP2R2A, PRSS22, RBFOX2, RDX, RNF44, RPUSD2, RRM2, SEMA4C, SENP5, SLC10A7, SLC16A9, SMCR8, SOCS1, SPATA2, SPRYD4, STRN, SURF4, SYT1, TGFBR1, TGFBR3, THBS1, TSC22D2, TXLNA, TXLNG, USP24, USP47, YOD1, ZBTB39, ZBTB5, ZNF644	**AGO1, DICER1**
	hsa-let-7c-5p	0.55	AGO1,BCL2L1,CASP3,CDC25A,CEBPB,COPS6,COPS8,GPS1,HMGA2,HSPA4,IGF1R,IL10,IL6,ITGB3,MAP4K3,MTOR,MYC,NRAS,NUMB,PBX2,RICTOR,STAT3,TGFBR1,TNFRSF10B,TRIB2,TRIM71	ACER2, AGO1, ANKRD46, ARID3B, BACH1, BCL2L1, BEND4, BZW1, CALU, CCNT2, CDV3, COIL, CPA4, DICER1, DNA2, DUSP7, DVL3, E2F6, EDEM3, EDN1, EPHA4, FAM104A, FIGN, FNDC3A, GOLGA4, GPAT4, HMGA1, ITGB3, KLHDC8B, KMT2D, MAP4K3, MDM4, MIEF1, MLLT10, MTUS1, MXD1, NAP1L1, NME4, NRAS, NSD1, PBX2, PBX3, PCGF3, PDE12, PDP2, PGRMC1, PLAGL2, PLD3, PPP1R15B, PPP2R2A, RAB11FIP4, RAMAC, RBFOX2, RDX, RFX6, RNF44, RRM2, SEMA4C, SLC10A7, SLC20A1, SLK, SMARCC1, SMC1A, SMCR8, STRN, SURF4, SYT1, TGFBR1, TGFBR3, THBS1, TXLNA, TXLNG, YOD1, ZNF644	**AGO1,BCL2L1,ITGB3,MAP4K3,NRAS,PBX2,TGFBR1**
Down regulated	hsa-miR-376c-3p	-0.33	ACVR1C,BCL2,BMI1,GRB2,IGF1R,NR5A2,RUNX2,TGFA,TGFBR1,UGT2B15,UGT2B17	UGT2B15	**UGT2B15**
	hsa-miR-377-3p	-0.40	DNMT1,E2F3,PAK1,PTEN,SOD1,SOD2,TIMP1,TP53	EGR1, HLF, INTS2, SOD2	**SOD2**
	hsa-miR-222-3p	-0.46	ABCG2,ADAM1A,ARID1A,BBC3,BMF,CDKN1B,CDKN1C,CERS2,CORO1A,DICER1,DIRAS3,DKK2,ESR1,ETS1,FOS,FOXO1,FOXO3,GAS5,GJA1,GNAI2,GNAI3,GRB10,ICAM1,KIT,MGMT,MMP1,PLXNC1,PPP2R2A,PRDM1,PTEN,RECK,RUNX2,SMAD5,SOD2,SSSCA1,SSX2IP,STAT5A,TCEAL1,TIMP3,TMED7,TNFSF10,TRPS1,VGLL4	BBC3, DCAF7, ESR1, ETS1, FOS, GNAI3, HIPK2, HMBOX1, PANK3, PHACTR4, PPP2R2A, RNF4, RUNX2, SNX4, STOX2, TLE3, TP53BP2, TRPS1, UBN2, VGLL4, ZFYVE16, ZNF652	**BBC3,ESR1,ETS1,FOS,GNAI3,PPP2R2A,RUNX2,TRPS1,VGLL4**
	hsa-miR-130a-3p	-0.52	Acvr1,ATG2B,ATXN1,CSF1,DICER1,DLL4,ESR1,GJA1,HOXA10,HOXA5,IFITM1,IL18,KLF4,MAFB,MAP3K12,MECP2,MEOX2,MYC,PDGFRA,PPARA,PPARG,PPARGC1A,PTEN,RAB5A,RUNX3,SLAIN1,SMAD4,TAC1,TGFB1,TGFBR2,TNF,XIAP	ACSL4, CAMSAP2, CDK19, DCBLD2, DDX6, DLC1, ESR1, KIF13A, LCLAT1, MAP3K9, MBNL3, MEOX2, MET, NFIB, PIGA, PPARG, PTPN4, QKI, RAB5B, SLAIN1, SOX4, TGFBR1, UBN2, WNK3, YY1, ZFYVE26, ZMAT3	**ESR1,MEOX2,PPARG,SLAIN1**
	hsa-miR-143-3p	-0.56	AKT1,AKT2,BAG3,BCL2,BRAF,CD44,COL3A1,CTGF,CYP2C9,DDX6,DNMT3A,DTNB,FAM83F,FHIT,FNDC3B,FSCN1,GABARAPL1,HK2,HNF4A,HRAS,IGF1R,IL13RA1,ITGB1,ITGB4,KLF5,KRAS,LIMK1,MACC1,MAPK7,MDM2,MMP13,MMP14,MMP2,MMP9,MYO6,NFATC1,NFKB2,NR2C2,OSBPL8,PTGS2,RREB1,SDC1,SERPINE1,TLR2,TNF	FNDC3B, KRAS, MAPK7, SAMD8, SECISBP2L, STOX2, ZBTB44	**FNDC3B,KRAS,MAPK7**
	hsa-miR-127-3p	-0.58	BAG5,BCL6,KMT5A,MAPK4,MGMT,MMP13,PRDM1,RGMA,SEPT7,SERPINB9,SFRP1,SKI,XBP1,XRCC3,ZWINT	SEPTIN7	ND
	hsa-miR-221-3p	-0.62	ADAM1A,ADAMTS6,ANXA1,APAF1,ARF4,ARNT,ASZ1,BBC3,BCL2L11,BECN1,BMF,BNIP3,BNIP3L,CDKN1B,CDKN1C,CERS2,CORO1A,CREBZF,CTCF,CXCL12,DDIT4,DICER1,DIRAS3,DKK2,DVL2,ESR1,ETS1,FMR1,FOS,FOXO3,GJA1,GRB10,HECTD2,HOXB5,ICAM1,KIT,MBD2,MDM2,MEOX2,MGMT,MMP2,MYBL1,NAIP,PAK1,PIK3R1,PTEN,RAB1A,RAD51,RB1,RECK,RUNX1,SOCS1,SOCS3,SSX2IP,STAT5A,STMN1,TBK1,TCEAL1,TICAM1,TIMP3,TMED7,TNFSF10,TRPS1,WEE1,ZEB2	ARID1A, BCL2L11, BMF, BRWD1, CHSY1, CTCF, ERBB4, ESR1, FBXO28, FMR1, KIT, LHFPL2, MAPK10, MIDN, NDFIP1, NUFIP2, PAIP2, PANK3, PCDHA1, PCDHA3, PCDHAC1, POGZ, PPP2R2A, STMN1, TMCC1, TMEM132B, TRPS1, UBE2J1, UBN2, ZNF652	**BCL2L11,BMF,CTCF,ESR1,FMR1,KIT,STMN1,TRPS1**
	hsa-miR-12136	-0.84	ND	ND	ND
	hsa-miR-22-3p	-0.86	ACLY,AKT1,ARPC5,BDNF,BMP6,BMP7,BMPR1B,BSG,BTG1,CCNA2,CD151,CDKN1A,CSF1R,CXCR2,CYR61,ERBB2,ERBB3,ESR1,HDAC4,HDAC6,HIF1A,HMGB1,HTR2C,IRF5,LGALS1,LGALS9,MALAT1,MAOA,MECOM,MMP14,MTA1,MTDH,MTHFR,MYCBP,NCOA1,NET1,NTRK2,PLK1,PPARA,PPM1K,PTEN,PTMS,RAB5B,RCOR1,RGS2,SIRT1,SLC2A1,SNAI1,SP1,TACC1,TCEAL1,TCF7,TET2,TFRC,TIAM1,UBR5,WNT1	ARHGEF26, ARPC5, BRWD3, C5orf24, CCNT2, CSF1R, EDC3, ESR1, MAX, MECOM, NCOA1, NET1, PDIK1L, RAB5B, RCOR1, SIRT1, SNAI1, SP1, TET2, TIAM1	**ARPC5,CSF1R,ESR1,MECOM,NCOA1,NET1,RAB5B,RCOR1,SIRT1,SNAI1,SP1,TET2,TIAM1**

Supplementary Table S3. Pathway analysis using miRPathDB v2.0 downstream of up and down regulated miRNAs in nicotine-treated DPSCs vs control.

Upregulated miRNAs were highlighted in red

Downregulated miRNAs were highlighted in blue

https://mpd.bioinf.uni-sb.de/

ND: No data

miRNA	KEGG Pathway	Evidence	P-value	Targets
**hsa-miR-4497**	Pentose phosphate pathway	experimental (any)	0.020	PRPS1, PRPS1L1
	Adrenergic signaling in cardiomyocytes	predicted (union)	0.022	ADCY1,ADCY9,ADRA1D,ATP1A1,ATP1B4,ATP2B3,CACNA1C,CACNG1,CACNG2,CACNG4,CACNG7,CACNG8,CALM3,CAMK2G,CREB3L3,GNAQ,GNAS,KCNE1,MAPK13,MAPK14,MAPK3,PIK3R1,PIK3R2,PLCB3,PPP2R2C,PPP2R5C,PRKCA,RYR2,SCN4B,SLC9A1,TPM3
	Calcium signaling pathway	predicted (union)	0.022	ADCY1,ADCY9,ADRA1D,ATP2B3,AVPR1A,CACNA1C,CALM3,CAMK2G,CHRM1,CHRM3,DRD1,GNA11,GNAL,GNAQ,GNAS,GRIN1,GRIN2A,GRM1,HRH1,ITPKC,NOS1,NTSR1,ORAI2,OXTR,P2RX7,PDE1C,PHKA1,PHKA2,PHKG2,PLCB3,PRKCA,RYR2,TACR1,TBXA2R
	Hippo signaling pathway	predicted (union)	0.022	AJUBA,APC2,AXIN1,BBC3,BMP7,BMP8A,BMP8B,BTRC,CSNK1D,DLG2,FRMD1,FRMD6,FZD10,GDF6,GDF7,GLI2,LATS1,LATS2,NF2,NKD1,PARD6G,PPP2R2C,SMAD7,TCF7,TCF7L2,TP73,WNT1,WNT5B,WNT8B,WNT9B,WTIP
	Ras signaling pathway	predicted (union)	0.022	ANGPT4,BCL2L1,CALM3,CSF1,EFNA2,FGF17,FGF22,FGFR3,FLT4,GNG2,GNG7,GRIN1,GRIN2A,GRIN2B,INSR,MAP2K2,MAPK3,MET,NGFR,PAK3,PAK4,PIK3R1,PIK3R2,PLA2G2D,PLA2G2F,PLA2G4F,PLAAT3,PRKCA,RAB5B,RALBP1,RASA3,RASA4,RASAL1,RASGRF1,RASGRF2,REL,RGL1,SHC2,SHC3,VEGFA,ZAP70
	Wnt signaling pathway	predicted (union)	0.022	APC2,AXIN1,BAMBI,BTRC,CAMK2G,CREBBP,CTBP1,CUL1,FOSL1,FRAT2,FZD10,NFATC2,NKD1,NKD2,PLCB3,PRICKLE2,PRKCA,PSEN1,SFRP1,SKP1,SOST,TBL1X,TBL1XR1,TCF7,TCF7L2,WNT1,WNT5B,WNT8B,WNT9B
	Insulin secretion	predicted (union)	0.028	ADCY1,ADCY9,ADCYAP1R1,ATP1A1,ATP1B4,CACNA1C,CAMK2G,CHRM3,CREB3L3,FFAR1,GNA11,GNAQ,GNAS,KCNMA1,KCNN1,KCNN3,KCNN4,PLCB3,PRKCA,RYR2
	Estrogen signaling pathway	predicted (union)	0.040	ADCY1,ADCY9,CALM3,CREB3L3,FKBP5,GABBR2,GNAO1,GNAQ,GNAS,GPER1,GRM1,HSPA6,MAP2K2,MAPK3,MMP2,PIK3R1,PIK3R2,PLCB3,SHC2,SHC3,SP1
	Biosynthesis of amino acids	experimental (any)	0.040	PRPS1, PRPS1L1
	Cardiac muscle contraction	experimental (any)	0.040	ATP1B4, TPM3
**hsa-miR-7977**	Proteoglycans in cancer	predicted (union)	0.022	ACTB,ACTG1,AKT2,ANK1,ANK2,ARAF,ARHGEF1,ARHGEF12,CAMK2A,CAMK2B,CAMK2D,CAMK2G,CASP3,CAV2,CBL,CBLB,CD44,CDC42,CDKN1A,COL21A1,CTTN,DCN,DDX5,EGFR,EIF4B,ELK1,ERBB2,ERBB4,ESR1,EZR,FGF1,FGF11,FGF12,FGF17,FGF18,FGF19,FGF20,FGF23,FGF4,FGF5,FGF9,FGFR1,FLNC,FN1,FRS2,FZD2,FZD3,FZD4,FZD5,FZD7,FZD8,GAB1,GPC1,HIF1A,HOXD10,HPSE,HPSE2,IGF1,IL12B,IQGAP1,ITGB1,ITGB3,ITPR2,KDR,KRAS,MAP2K1,MAPK1,MAPK11,MAPK13,MAPK14,MET,MMP9,MRAS,NANOG,NRAS,NUDT16L1,PDCD4,PDPK1,PIK3CA,PIK3CG,PIK3R2,PIK3R3,PIK3R5,PLAU,PLCE1,PLCG1,PPP1R12A,PPP1R12B,PPP1R12C,PRKACA,PRKACG,PRKCA,PRKCG,PRKX,PTCH1,PTPN11,PTPN6,PXN,RAF1,ROCK1,ROCK2,RPS6,RPS6KB1,RPS6KB2,SDC1,SDC2,SDC4,SMAD2,SMO,SRC,STAT3,TFAP4,TGFB1,THBS1,TIMP3,TNF,TP53,TWIST1,VAV2,VEGFA,WNT10B,WNT16,WNT2B,WNT3,WNT3A,WNT4,WNT7A,WNT7B,WNT8A,WNT9A,WNT9B
	Circadian entrainment	predicted (union)	0.041	ADCY1,ADCY2,ADCY3,ADCY5,ADCY9,ADCYAP1R1,CACNA1C,CACNA1G,CACNA1H,CACNA1I,CALM1,CALM3,CALML3,CALML5,CAMK2A,CAMK2B,CAMK2D,CAMK2G,CREB1,GNAI3,GNAO1,GNAQ,GNAS,GNB4,GNG10,GNG11,GNG2,GNG3,GNG4,GNG7,GNGT2,GRIA4,GRIN2A,GRIN2B,GRIN2C,GRIN2D,GUCY1A1,GUCY1A2,GUCY1B1,KCNJ3,KCNJ5,KCNJ6,KCNJ9,MAPK1,MTNR1B,NOS1,NOS1AP,PER1,PER2,PER3,PLCB1,PLCB2,PLCB4,PRKACA,PRKACG,PRKCA,PRKCG,PRKG1,PRKG2,PRKX,RASD1,RYR2
	Glycosaminoglycan biosynthesis - heparan sulfate / heparin	predicted (union)	0.041	B3GALT6,B3GAT3,B4GALT7,EXT1,EXT2,EXTL1,EXTL2,EXTL3,HS2ST1,HS3ST1,HS3ST2,HS3ST3A1,HS3ST3B1,HS6ST1,HS6ST3,NDST1,NDST2,NDST3,NDST4,XYLT1
**hsa-miR-3178**	TNF signaling pathway	experimental (any)	0.044	JUN, JUNB,MAP2K7,TRAF3
**hsa-miR-1260b**	Wnt signaling pathway	experimental (strong)	2.87e-5	DKK2, SFRP1,SMAD4
	Rap1 signaling pathway	predicted (union)	0.008	ACTG1,ADCY1,ADCY2,ADCY3,ADCY5,ADCY9,AFDN,AKT2,ANGPT4,CALM3,CALML3,CDC42,CRK,CRKL,CSF1,CSF1R,CTNNB1,EFNA1,EFNA2,EFNA3,EFNA5,EGFR,F2RL3,FGF1,FGF10,FGF12,FGF14,FGF16,FGF19,FGF2,FGF22,FGF3,FGF5,FGFR1,FGFR3,FLT1,FLT4,FPR1,GNAI3,GNAO1,GNAS,GRIN2A,GRIN2B,ID1,IGF1,INSR,ITGAL,ITGB3,KDR,KIT,KRAS,KRIT1,LCP2,LPAR4,LPAR5,MAGI1,MAP2K1,MAP2K2,MAP2K3,MAP2K6,MAPK1,MAPK11,MAPK13,MAPK14,MAPK3,MET,MRAS,NGFR,P2RY1,PARD3,PDGFA,PDGFB,PDGFD,PGF,PIK3CB,PIK3CG,PIK3R1,PIK3R3,PLCB2,PLCB3,PLCE1,PLCG1,PRKCA,PRKCG,PRKCI,PRKD1,PRKD2,RAC1,RALA,RALB,RALGDS,RAP1B,RAP1GAP,RAPGEF1,RAPGEF3,RAPGEF5,RAPGEF6,RASGRP2,RASGRP3,RHOA,SIPA1,SIPA1L3,SKAP1,SRC,THBS1,TLN1,TLN2,VASP,VAV2
	Glycosaminoglycan biosynthesis - heparan sulfate / heparin	predicted (union)	0.013	B3GALT6,B3GAT3,B4GALT7,EXT2,EXTL3,HS2ST1,HS3ST1,HS3ST2,HS3ST3A1,HS3ST3B1,HS3ST5,HS6ST1,HS6ST3,NDST1,NDST2,NDST3,NDST4,XYLT1,XYLT2
	Carbon metabolism	experimental (any)	0.025	ACAT1, ALDOA,ENO1,IDH2,PDHB,PKM,PSAT1,RPE

	Ribosome	experimental (any)	0.026	MRPL24, RPL10A,RPL3,RPL36A,RPL4,RPS15,RPS27,RPS6,RPS7
	Biosynthesis of amino acids	experimental (any)	0.028	ALDOA, ENO1,IDH2,PKM,PSAT1,RPE
	Neurotrophin signaling pathway	predicted (union)	0.030	ABL1,AKT2,ARHGDIA,BAX,BCL2,CALM3,CALML3,CAMK2A,CAMK2B,CAMK2D,CAMK2G,CAMK4,CDC42,CRK,CRKL,FRS2,GAB1,GSK3B,IRAK2,IRAK3,IRAK4,IRS1,JUN,KRAS,MAP2K1,MAP2K2,MAP2K7,MAP3K3,MAP3K5,MAPK1,MAPK11,MAPK13,MAPK14,MAPK3,MAPK8,MAPK9,MAPKAPK2,NFKBIA,NFKBIB,NGFR,NTRK1,NTRK2,PDPK1,PIK3CB,PIK3CG,PIK3R1,PIK3R3,PLCG1,PRDM4,PRKCD,PSEN1,PTPN11,RAC1,RAP1B,RAPGEF1,RHOA,RPS6KA2,RPS6KA3,RPS6KA6,SH2B2,SH2B3,SHC3,SORT1,TRAF6,ZNF274
	Pathways in cancer	predicted (union)	0.030	ABL1,AKT2,APC2,AR,ARNT2,BAX,BCL2,BCR,BID,CASP9,CBL,CBLB,CCDC6,CCND1,CCNE2,CDC42,CDK2,CDK6,CDKN1B,CDKN2B,CEBPA,CKS1B,COL4A2,COL4A4,COL4A5,CRK,CRKL,CSF1R,CSF3R,CTNNA2,CTNNA3,CTNNB1,CYCS,DAPK3,DVL1,E2F1,E2F3,EGFR,EGLN1,EP300,ERBB2,FADD,FGF1,FGF10,FGF12,FGF14,FGF16,FGF19,FGF2,FGF22,FGF3,FGF5,FGFR1,FGFR3,FLT3,FN1,FOS,FZD4,FZD5,FZD6,FZD8,GLI3,GSK3B,HDAC2,HIF1A,HSP90B1,IGF1,ITGA2,ITGAV,JUN,JUP,KIT,KLK3,KRAS,LAMA4,LAMB4,LAMC1,LAMC2,LAMC3,LEF1,MAP2K1,MAP2K2,MAPK1,MAPK3,MAPK8,MAPK9,MECOM,MET,MLH1,MMP2,MSH2,MSH3,NFKBIA,NKX3-1,NTRK1,PAX8,PDGFA,PDGFB,PGF,PIK3CB,PIK3CG,PIK3R1,PIK3R3,PLCG1,PML,PPARD,PRKCA,PRKCG,PTCH1,PTEN,RAC1,RAD51,RALA,RALB,RALBP1,RALGDS,RARA,RB1,RBX1,RET,RHOA,RUNX1,RUNX1T1,RXRA,RXRB,SKP2,SLC2A1,SMAD3,SMO,STAT1,STAT3,STK4,SUFU,TCF7,TCF7L2,TGFB1,TGFBR1,TGFBR2,TPM3,TRAF1,TRAF2,TRAF4,TRAF5,TRAF6,VHL,WNT10B,WNT2B,WNT3,WNT3A,WNT5B,WNT6,WNT8B,WNT9A,WNT9B,XIAP,ZBTB16
	Estrogen signaling pathway	predicted (union)	0.033	ADCY1,ADCY2,ADCY3,ADCY5,ADCY9,AKT2,ATF2,ATF6B,CALM3,CALML3,CREB1,CREB3L2,CREB5,EGFR,ESR2,FKBP4,FKBP5,FOS,GABBR1,GABBR2,GNAI3,GNAO1,GNAS,GPER1,HBEGF,HSP90B1,HSPA1B,HSPA6,ITPR2,JUN,KCNJ3,KCNJ6,KCNJ9,KRAS,MAP2K1,MAP2K2,MAPK1,MAPK3,MMP2,OPRM1,PIK3CB,PIK3CG,PIK3R1,PIK3R3,PLCB2,PLCB3,PRKACA,PRKACB,PRKCD,PRKX,SHC3,SP1,SRC
	Morphine addiction	predicted (union)	0.033	ADCY1,ADCY2,ADCY3,ADCY5,ADCY9,ADORA1,ARRB1,ARRB2,CACNA1A,GABBR1,GABBR2,GABRA3,GABRA4,GABRB1,GABRD,GABRE,GABRG1,GABRP,GABRR2,GNAI3,GNAO1,GNAS,GNB1,GNB5,GNG4,GNG7,GRK3,GRK5,GRK6,KCNJ3,KCNJ6,KCNJ9,OPRM1,PDE10A,PDE11A,PDE1A,PDE1C,PDE2A,PDE3A,PDE3B,PDE4A,PDE4D,PDE7A,PDE7B,PRKACA,PRKACB,PRKCA,PRKCG,PRKX
	Type II diabetes mellitus	predicted (union)	0.033	CACNA1A,CACNA1C,GCK,HK2,HKDC1,INSR,IRS1,IRS2,IRS4,KCNJ11,MAFA,MAPK1,MAPK3,MAPK8,MAPK9,PDX1,PIK3CB,PIK3CG,PIK3R1,PIK3R3,PKLR,PKM,PRKCD,PRKCE,SLC2A4,SOCS2,SOCS3,SOCS4
	Insulin signaling pathway	predicted (union)	0.038	ACACB,AKT2,CALM3,CALML3,CBL,CBLB,CRK,CRKL,EIF4E,EIF4E1B,EIF4E2,EIF4EBP1,ELK1,FASN,G6PC,G6PC2,GCK,GSK3B,HK2,HKDC1,INPPL1,INSR,IRS1,IRS2,IRS4,KRAS,MAP2K1,MAP2K2,MAPK1,MAPK3,MAPK8,MAPK9,PDE3A,PDE3B,PDPK1,PHKA2,PHKG1,PHKG2,PIK3CB,PIK3CG,PIK3R1,PIK3R3,PKLR,PPARGC1A,PPP1CB,PPP1R3B,PPP1R3C,PPP1R3D,PPP1R3E,PPP1R3F,PRKAA1,PRKAB1,PRKAB2,PRKACA,PRKACB,PRKAR1B,PRKAR2A,PRKAR2B,PRKCI,PRKX,PTPN1,PYGB,RAPGEF1,SH2B2,SHC3,SLC2A4,SOCS2,SOCS3,SOCS4,SORBS1,TSC1
	Adrenergic signaling in cardiomyocytes	predicted (union)	0.044	ACTC1,ADCY1,ADCY2,ADCY3,ADCY5,ADCY9,ADRA1A,ADRA1B,ADRA1D,AKT2,ATF2,ATF6B,ATP1A2,ATP1A3,ATP1B3,ATP1B4,ATP2B1,ATP2B3,ATP2B4,BCL2,CACNA1C,CACNA2D1,CACNA2D2,CACNA2D4,CACNB1,CACNB2,CACNB4,CACNG2,CACNG6,CACNG7,CACNG8,CALM3,CALML3,CAMK2A,CAMK2B,CAMK2D,CAMK2G,CREB1,CREB3L2,CREB5,GNAI3,GNAS,KCNE1,MAPK1,MAPK11,MAPK13,MAPK14,MAPK3,MYL3,PIK3CB,PIK3CG,PIK3R1,PIK3R3,PLCB2,PLCB3,PPP1CB,PPP1R1A,PPP2CA,PPP2R1A,PPP2R2A,PPP2R2C,PPP2R5E,PRKACA,PRKACB,PRKCA,PRKX,RAPGEF3,SCN1B,SCN4B,SLC8A1,SLC9A1,TNNT2,TPM3,TPM4
	GABAergic synapse	predicted (union)	0.044	ABAT,ADCY1,ADCY2,ADCY3,ADCY5,ADCY9,CACNA1A,CACNA1C,GABARAP,GABARAPL1,GABBR1,GABBR2,GABRA3,GABRA4,GABRB1,GABRD,GABRE,GABRG1,GABRP,GABRR2,GAD1,GAD2,GLS2,GLUL,GNAI3,GNAO1,GNB1,GNB5,GNG4,GNG7,HAP1,KCNJ6,NSF,PLCL1,PRKACA,PRKACB,PRKCA,PRKCG,PRKX,SLC12A5,SLC38A1,SLC38A2,SLC6A1,SLC6A11,SRC,TRAK2
	MAPK signaling pathway	predicted (union)	0.044	AKT2,ARRB1,ARRB2,ATF2,CACNA1A,CACNA1C,CACNA1H,CACNA1I,CACNA2D1,CACNA2D2,CACNA2D4,CACNB1,CACNB2,CACNB4,CACNG2,CACNG6,CACNG7,CACNG8,CDC25B,CDC42,CRK,CRKL,DAXX,DUSP10,DUSP16,DUSP2,DUSP3,DUSP4,DUSP5,DUSP7,DUSP8,EGFR,ELK1,ELK4,FGF1,FGF10,FGF12,FGF14,FGF16,FGF19,FGF2,FGF22,FGF3,FGF5,FGFR1,FGFR3,FOS,GADD45G,HSPA1B,HSPA6,JMJD7-PLA2G4B,JUN,KRAS,MAP2K1,MAP2K2,MAP2K3,MAP2K6,MAP2K7,MAP3K12,MAP3K13,MAP3K20,MAP3K3,MAP3K5,MAP4K2,MAP4K4,MAPK1,MAPK11,MAPK13,MAPK14,MAPK3,MAPK8,MAPK8IP3,MAPK9,MAPKAPK2,MAPKAPK3,MAPKAPK5,MAPT,MECOM,MEF2C,MRAS,NF1,NFATC3,NLK,NTRK1,NTRK2,PDGFA,PDGFB,PLA2G4F,PPM1A,PPM1B,PPP3R1,PRKACA,PRKACB,PRKCA,PRKCG,PRKX,PTPN7,RAC1,RAP1B,RASA1,RASA2,RASGRF2,RASGRP1,RASGRP2,RASGRP3,RPS6KA2,RPS6KA3,RPS6KA6,SRF,STK4,STMN1,TAB1,TAB2,TAOK1,TAOK2,TGFB1,TGFBR1,TGFBR2,TRAF2,TRAF6
	HIF-1 signaling pathway	experimental (any)	0.045	ALDOA, CDKN1A,CDKN1B,ENO1,MKNK2,PDHB,RPS6
**hsa-miR-10400-5p**	Endometrial cancer	predicted (union)	0.015	AKT2,APC2,AXIN1,BAD,CTNNA1,EGFR,ELK1,FOXO3,GSK3B,MAP2K2,MAPK1,PDPK1,PIK3R2,RAF1,TCF7,TCF7L1,TCF7L2
	ErbB signaling pathway	predicted (union)	0.015	ABL1,AKT2,BAD,CAMK2A,CAMK2G,CBL,CDKN1A,CRKL,EGFR,ELK1,GSK3B,JUN,MAP2K2,MAP2K7,MAPK1,NRG2,PIK3R2,PRKCA,PRKCG,RAF1,RPS6KB2,SHC2,SRC,STAT5A
	Wnt signaling pathway	predicted (union)	0.015	APC2,AXIN1,CAMK2A,CAMK2G,CCND3,CREBBP,CSNK1E,CSNK2A2,CTBP1,CTNNBIP1,CUL1,FOSL1,FRAT1,FRAT2,FZD1,GSK3B,JUN,MAP3K7,NFATC1,NKD2,PPARD,PRKCA,PRKCG,PRKX,RAC3,SOST,TBL1X,TCF7,TCF7L1,TCF7L2,VANGL2,WNT2B,WNT6,WNT8B,WNT9B
	Melanogenesis	predicted (union)	0.023	ADCY1,ADCY9,CALML5,CAMK2A,CAMK2G,CREB3L3,CREBBP,FZD1,GNAI2,GNAO1,GNAQ,GNAS,GSK3B,MAP2K2,MAPK1,POMC,PRKCA,PRKCG,PRKX,RAF1,TCF7,TCF7L1,TCF7L2,WNT2B,WNT6,WNT8B,WNT9B
	SNARE interactions in vesicular transport	predicted (union)	0.027	BNIP1, GOSR1,SNAP25,SNAP47,STX11,STX1A,STX1B,STX4,STX5,STX6,VAMP1,VAMP7,VTI1A
	Axon guidance	predicted (union)	0.030	ABL1,CXCL12,DPYSL5,EFNA2,EPHA4,EPHA8,EPHB2,GNAI2,GSK3B,L1CAM,MAPK1,NGEF,NTN1,NTN3,NTNG2,PLXNA1,PLXNA2,PLXNA3,PLXNB1,PLXNB2,RAC3,RHOD,SEMA3F,SEMA4B,SEMA4C,SEMA4D,SEMA5B,SEMA7A,SLIT1,SRGAP3

**hsa-let-7e-5p**	MicroRNAs in cancer	experimental (any)	8.70e-4	CCND1,CCNG1,CDKN1A,CREBBP,CRK,EZH2,HMGA2,IGF2BP1,IRS2,MDM4,MMP9,MYC,PDGFB,PLCG2,RDX,SOCS1,STAT3,THBS1,TIMP3,TRIM71
	FoxO signaling pathway	experimental (strong)	0.002	CCND1, FASLG,IGF1,IGF1R,PLK1
	Oocyte meiosis	experimental (strong)	0.002	IGF1, IGF1R,PLK1,SMC1A
	Proteoglycans in cancer	experimental (strong)	0.002	CCND1, FASLG,IGF1,IGF1R,MMP9,WNT1
	Transcriptional misregulation in cancer	experimental (strong)	0.002	HMGA2, IGF1,IGF1R,MMP9,MYCN
	Measles	experimental (strong)	0.007	CCND1, FASLG,TNFAIP3,TNFRSF10B
	Pathways in cancer	experimental (strong)	0.010	CCND1, FASLG,IGF1,IGF1R,MMP9,WNT1
	Glioma	experimental (strong)	0.011	CCND1, IGF1,IGF1R
	Progesterone-mediated oocyte maturation	experimental (strong)	0.011	IGF1, IGF1R,PLK1
	p53 signaling pathway	experimental (strong)	0.011	CCND1, IGF1,TNFRSF10B
	Melanoma	experimental (strong)	0.012	CCND1, IGF1,IGF1R
	Ovarian steroidogenesis	experimental (strong)	0.015	IGF1, IGF1R
	Cell cycle	experimental (strong)	0.022	CCND1, PLK1,SMC1A
	Long-term depression	experimental (strong)	0.022	IGF1, IGF1R
	MicroRNAs in cancer	experimental (strong)	0.022	CCND1, EZH2,HMGA2,MMP9
	Prostate cancer	experimental (strong)	0.022	CCND1, IGF1,IGF1R
	FoxO signaling pathway	predicted (intersection)	0.034	AKT2,ATG12,ATM,CCNB1,CCND2,CCNG2,CDKN1A,CHUK,EGF,EGFR,FASLG,FBXO32,FOXO1,GADD45G,IGF1,IL10,INSR,IRS1,IRS2,KLF2,KRAS,MAPK1,MAPK11,MAPK14,MAPK8,MAPK9,NLK,NRAS,PIK3CA,PRKAA2,PRKAB2,SETD7,SKP2,SMAD2,STAT3,STK4,TGFBR1,TGFBR2,TNFSF10
	MAPK signaling pathway	predicted (intersection)	0.034	AKT2,ARRB1,CACNB2,CACNB4,CACNG4,CASP3,CDC25B,CHUK,DUSP1,DUSP16,DUSP2,DUSP4,DUSP6,DUSP7,EGF,EGFR,ELK1,ELK4,FAS,FASLG,FGF11,FGF13,FGF5,FGFR1,FOS,GADD45G,KRAS,MAP2K6,MAP2K7,MAP3K1,MAP3K13,MAP3K3,MAP4K3,MAP4K4,MAPK1,MAPK11,MAPK14,MAPK8,MAPK9,MAPKAPK5,MRAS,NGF,NLK,NRAS,PAK1,PDGFA,PDGFB,PLA2G4A,PLA2G4E,PPP3R1,PRKACB,PTPN7,RAC1,RASGRF1,RASGRF2,RASGRP1,RPS6KA2,RPS6KA3,RPS6KA6,STK4,TAB1,TAOK1,TGFBR1,TGFBR2,TP53,TRAF6
	Bladder cancer	experimental (strong)	0.037	CCND1, MMP9
	PI3K-Akt signaling pathway	experimental (strong)	0.040	CCND1, FASLG,IGF1,IGF1R
	Epstein-Barr virus infection	experimental (any)	0.042	CDKN1A,CREBBP,HLAC,LYN,MAP2K7,MYC,PLCG2,POLR2D,POLR3D,PSMD2,SPN,STAT3,TNFAIP3,YWHAE,YWHAG,YWHAQ,YWHAZ
	FoxO signaling pathway	experimental (any)	0.042	ATG12, CCND1,CDKN1A,CREBBP,FASLG,IGF1,IGF1R,IRS2,IRS4,PLK1,SOD2,STAT3,STK4
	Pathogenic Escherichia coli infection	experimental (any)	0.042	OCLN, TUBA1B,TUBB2A,TUBB4A,TUBB4B,WASL,YWHAQ,YWHAZ
	p53 signaling pathway	experimental (any)	0.042	CCND1, CCNG1,CDKN1A,IGF1,MDM4,PMAIP1,RRM2,THBS1,TNFRSF10B
	Oocyte meiosis	experimental (any)	0.044	BTRC, ESPL1,IGF1,IGF1R,PLK1,PPP2R1A,SMC1A,YWHAE,YWHAG,YWHAQ,YWHAZ
	Hepatitis B	experimental (strong)	0.045	CCND1, FASLG,MMP9
	Ras signaling pathway	experimental (strong)	0.049	FASLG, IGF1,IGF1R
**hsa-let-7a-5p**	MicroRNAs in cancer	experimental (strong)	1.54e-9	CASP3,CCND2,CDK6,CDKN1A,DICER1,E2F2,EGFR,EZH2,HMGA2,HRAS,IGF2BP1,ITGB3,KRAS,MYC,NRAS,STAT3,TRIM71
	MicroRNAs in cancer	experimental (any)	2.08e-9	BCL2,BCL2L11,CASP3,CCND1,CCND2,CCNG1,CDC25B,CDK6,CDKN1A,DICER1,E2F2,EGFR,EZH2,HMGA2,HRAS,IGF2BP1,ITGB3,KRAS,MDM4,MYC,NFKB1,NRAS,PDGFB,PLCG2,RDX,SOCS1,STAT3,THBS1,TRIM71
	p53 signaling pathway	experimental (any)	3.40e-5	CASP3,CCNB2,CCND1,CCND2,CCNG1,CDK6,CDKN1A,MDM4,PMAIP1,RRM2,SESN1,SESN2,THBS1,TNFRSF10B
	Bladder cancer	experimental (strong)	4.75e-5	CDKN1A, E2F2,EGFR,HRAS,KRAS,MYC,NRAS
	Bladder cancer	experimental (any)	8.55e-5	CCND1, CDKN1A,CXCL8,E2F2,EGFR,HRAS,KRAS,MYC,NRAS,THBS1
	FoxO signaling pathway	experimental (any)	8.55e-5	ATG12,BCL2L11,CCNB2,CCND1,CCND2,CDKN1A,EGFR,HRAS,IGF1R,IL6,KRAS,MAPK11,NRAS,PIK3R1,SETD7,SOD2,STAT3,STK4
	Glioma	experimental (any)	8.55e-5	CAMK2D, CCND1,CDK6,CDKN1A,E2F2,EGFR,HRAS,IGF1R,KRAS,NRAS,PDGFB,PIK3R1,PLCG2

Hepatitis B	experimental (any)	8.55e-5	BCL2,CASP3,CCND1,CDK6,CDKN1A,CXCL8,E2F2,EGR3,HRAS,IL6,KRAS,MAP3K1,MYC,NFKB1,NRAS,PIK3R1,STAT2,STAT3,YWHAZ
Proteoglycans in cancer	experimental (strong)	1.23e-4	CASP3, CDKN1A,EGFR,HRAS,IGF2,ITGB3,KRAS,MYC,NRAS,PAK1,STAT3,WNT1
Hepatitis B	experimental (strong)	1.49e-4	CASP3, CDK6,CDKN1A,E2F2,HRAS,IL6,KRAS,MYC,NRAS,STAT3
Glioma	experimental (strong)	1.76e-4	CDK6, CDKN1A,E2F2,EGFR,HRAS,KRAS,NRAS
Melanoma	experimental (strong)	2.47e-4	CDK6, CDKN1A,E2F2,EGFR,HRAS,KRAS,NRAS
Viral carcinogenesis	experimental (strong)	2.47e-4	CASP3, CCND2,CDK6,CDKN1A,HRAS,KRAS,NRAS,PKM,STAT3
Viral carcinogenesis	experimental (any)	4.44e-4	CASP3,CCND1,CCND2,CDK6,CDKN1A,EGR3,HDAC5,HIST1H2BD,HIST1H2BK,HIST1H4D,HIST2H2BE,HIST2H2BF,HRAS,KRAS,LYN,NFKB1,NRAS,PIK3R1,PKM,PMAIP1,STAT3,YWHAZ
Chronic myeloid leukemia	experimental (strong)	6.07e-4	CDK6, CDKN1A,E2F2,HRAS,KRAS,MYC,NRAS
ErbB signaling pathway	experimental (strong)	6.07e-4	CDKN1A, EGFR,HRAS,KRAS,MYC,NRAS,PAK1
Non-small cell lung cancer	experimental (strong)	6.14e-4	CDK6, E2F2,EGFR,HRAS,KRAS,NRAS
p53 signaling pathway	experimental (strong)	6.14e-4	CASP3, CCND2,CDK6,CDKN1A,RRM2,TNFRSF10B
Proteoglycans in cancer	experimental (any)	6.64e-4	ACTB,CAMK2D,CASP3,CCND1,CDKN1A,EGFR,FZD9,HRAS,IGF1R,IGF2,ITGB3,KRAS,MAPK11,MYC,NRAS,PAK1,PIK3R1,PLCG2,RDX,STAT3,THBS1,VAV2,WNT1
Epithelial cell signaling in Helicobacter pylori infection	experimental (any)	7.89e-4	ATP6V1B2, ATP6V1F,ATP6V1G1,CASP3,CXCL8,EGFR,LYN,MAPK11,NFKB1,PAK1,PLCG2
FoxO signaling pathway	experimental (strong)	9.08e-4	CCND2, CDKN1A,EGFR,HRAS,IL6,KRAS,NRAS,STAT3
Natural killer cell mediated cytotoxicity	experimental (strong)	9.51e-4	CASP3, HRAS,KRAS,NRAS,PAK1,TNFRSF10B
Melanoma	experimental (any)	0.001	CCND1, CDK6,CDKN1A,E2F2,EGFR,HRAS,IGF1R,KRAS,NRAS,PDGFB,PIK3R1
Non-small cell lung cancer	experimental (any)	0.001	CCND1, CDK6,E2F2,EGFR,HRAS,KRAS,NRAS,PIK3R1,PLCG2,STK4
Prolactin signaling pathway	experimental (any)	0.001	CCND1, CCND2,HRAS,KRAS,MAPK11,NFKB1,NRAS,PIK3R1,SOCS1,SOCS7,STAT3
Pathways in cancer	experimental (strong)	0.002	CASP3, CDK6,CDKN1A,E2F2,EGFR,HRAS,IL6,KRAS,MYC,NRAS,STAT3,WNT1
Thyroid cancer	experimental (strong)	0.002	HRAS, KRAS,MYC,NRAS
Prostate cancer	experimental (any)	0.002	BCL2, CCND1,CDKN1A,E2F2,EGFR,HRAS,IGF1R,KRAS,NFKB1,NRAS,PDGFB,PIK3R1
Endometrial cancer	experimental (strong)	0.003	EGFR, HRAS,KRAS,MYC,NRAS
HTLV-I infection	experimental (strong)	0.003	CCND2, CDKN1A,E2F2,HRAS,IL6,KRAS,MYC,NRAS,WNT1
PI3K-Akt signaling pathway	experimental (strong)	0.003	CCND2, CDK6,CDKN1A,EGFR,HRAS,IL6,ITGB3,KRAS,MYC,NRAS
Transcriptional misregulation in cancer	experimental (strong)	0.003	CCND2, CCR7,CDKN1A,EWSR1,HMGA2,IL6,MYC
Acute myeloid leukemia	experimental (strong)	0.004	HRAS, KRAS,MYC,NRAS,STAT3
Hepatitis C	experimental (strong)	0.004	CDKN1A, EGFR,HRAS,KRAS,NRAS,STAT3
Prostate cancer	experimental (strong)	0.004	CDKN1A, E2F2,EGFR,HRAS,KRAS,NRAS
MAPK signaling pathway	experimental (strong)	0.006	CASP3, EGFR,HRAS,KRAS,MAP4K4,MYC,NRAS,PAK1
Measles	experimental (strong)	0.006	CCND2, CDK6,IL6,STAT3,TNFAIP3,TNFRSF10B
Prolactin signaling pathway	experimental (strong)	0.006	CCND2, HRAS,KRAS,NRAS,STAT3
Pancreatic cancer	experimental (strong)	0.007	CDK6, E2F2,EGFR,KRAS,STAT3
Serotonergic synapse	experimental (strong)	0.009	CASP3, HRAS,KRAS,NRAS
Chronic myeloid leukemia	experimental (any)	0.009	CCND1, CDK6,CDKN1A,E2F2,HRAS,KRAS,MYC,NFKB1,NRAS,PIK3R1
ErbB signaling pathway	experimental (any)	0.009	CAMK2D, CDKN1A,EGFR,HRAS,KRAS,MAP2K7,MYC,NRAS,PAK1,PIK3R1,PLCG2

HIF-1 signaling pathway	experimental (any)	0.009	BCL2, CAMK2D,CDKN1A,EDN1,EGFR,IGF1R,IL6,IL6R,NFKB1,PIK3R1,PLCG2,STAT3
Chemokine signaling pathway	experimental (strong)	0.010	CCR7, HRAS,KRAS,NRAS,PAK1,STAT3
Ribosome	experimental (any)	0.011	MRPL12, MRPL15,MRPS2,RPL30,RPL35A,RPL4,RPL9,RPS10,RPS13,RPS15A,RPS24,RPS3A,RPS4X,RPSA
FoxO signaling pathway	predicted (intersection)	0.012	AKT2,ATG12,ATM,CCNB1,CCND2,CCNG2,CDKN1A,CHUK,EGF,EGFR,FASLG,FBXO32,FOXO1,GADD45G,HOMER1,IGF1,IL10,INSR,IRS1,IRS2,KLF2,MAPK1,MAPK11,MAPK8,MAPK9,NLK,NRAS,PIK3CA,PRKAA2,PRKAB2,SETD7,SKP2,SMAD2,STAT3,STK4,TGFBR1,TGFBR2,TNFSF10
MicroRNAs in cancer	predicted (intersection)	0.012	ABCC1,APC2,ATM,BAK1,BCL2,BCL2L2,BMF,CASP3,CCND2,CD44,CDC25A,CDC25B,CDK6,CDKN1A,DNMT3A,E2F2,EGFR,FZD3,GLS,HMGA2,IGF2BP1,IRS1,IRS2,ITGB3,MAPK1,MDM4,MMP16,NOTCH2,NRAS,PDCD4,PDGFA,PDGFB,PIK3CA,RDX,ROCK1,SHC1,SOCS1,STAT3,THBS1,TNR,TP53,TP63,TRIM71
Gap junction	experimental (strong)	0.012	EGFR, HRAS,KRAS,NRAS
Regulation of actin cytoskeleton	experimental (strong)	0.012	EGFR, HRAS,ITGB3,KRAS,NRAS,PAK1
Fc epsilon RI signaling pathway	experimental (any)	0.012	HRAS, KRAS,LYN,MAP2K7,MAPK11,NRAS,PIK3R1,PLCG2,VAV2
Cell cycle	experimental (any)	0.013	CCNB2, CCND1,CCND2,CDC25B,CDC7,CDK6,CDKN1A,E2F2,ESPL1,MYC,RAD21,SMC1A,YWHAZ
Thyroid hormone signaling pathway	experimental (strong)	0.014	HRAS, ITGB3,KRAS,MYC,NRAS
Epstein-Barr virus infection	experimental (any)	0.014	BCL2,CDKN1A,CSNK2A1,HDAC5,LYN,MAP2K7,MAPK11,MYC,NFKB1,PIK3R1,PLCG2,POLR2D,POLR3D,STAT3,TAB2,TNFAIP3,YWHAZ
PI3K-Akt signaling pathway	experimental (any)	0.014	BCL2,BCL2L11,CCND1,CCND2,CDK6,CDKN1A,EGFR,F2R,GNG5,HRAS,IGF1R,IL6,IL6R,ITGA3,ITGB3,KRAS,MYC,NFKB1,NRAS,PDGFB,PIK3R1,PPP2R2A,THBS1,YWHAZ
Thyroid hormone signaling pathway	experimental (any)	0.014	ACTB, ATP2A2,CCND1,HRAS,ITGB3,KRAS,MED13L,MYC,NCOA3,NRAS,PIK3R1,PLCG2
Acute myeloid leukemia	experimental (any)	0.016	CCND1, HRAS,KRAS,MYC,NFKB1,NRAS,PIK3R1,STAT3
Hepatitis C	experimental (any)	0.016	CDKN1A, CXCL8,EGFR,HRAS,KRAS,MAPK11,NFKB1,NRAS,PIK3R1,PPP2R2A,STAT2,STAT3
Cell cycle	experimental (strong)	0.018	CCND2, CDK6,CDKN1A,E2F2,MYC
GnRH signaling pathway	experimental (strong)	0.018	EGFR, HRAS,KRAS,NRAS
Long-term depression	experimental (strong)	0.021	HRAS, KRAS,NRAS
Measles	experimental (any)	0.023	CCND1, CCND2,CDK6,CSNK2A1,IL6,NFKB1,PIK3R1,STAT2,STAT3,TAB2,TNFAIP3,TNFRSF10B
Pathways in cancer	experimental (any)	0.023	BCL2,CASP3,CCND1,CDK6,CDKN1A,CXCL8,DVL3,E2F2,EGFR,FZD9,HRAS,IGF1R,IL6,ITGA3,KRAS,MYC,NFKB1,NRAS,PDGFB,PIK3R1,PLCG2,STAT3,STK4,WNT1
Estrogen signaling pathway	experimental (strong)	0.026	EGFR, HRAS,KRAS,NRAS
Melanogenesis	experimental (strong)	0.027	HRAS, KRAS,NRAS,WNT1
Renal cell carcinoma	experimental (strong)	0.028	HRAS, KRAS,NRAS,PAK1
Endometrial cancer	experimental (any)	0.030	CCND1, EGFR,HRAS,KRAS,MYC,NRAS,PIK3R1
Thyroid cancer	experimental (any)	0.030	CCND1, HRAS,KRAS,MYC,NRAS
MicroRNAs in cancer	predicted (union)	0.034	ABCC1,ABL1,APC2,ATM,BAK1,BCL2,BCL2L2,BMF,BMPR2,CASP3,CCND1,CCND2,CCNE2,CCNG1,CD44,CDC25A,CDC25B,CDK6,CDKN1A,CRK,CRKL,DICER1,DNMT3A,DNMT3B,E2F2,EGFR,EZR,FOXP1,FZD3,GLS,HDAC4,HMGA2,HNRNPK,IGF2BP1,IRS1,IRS2,ITGB3,KRAS,MAP2K1,MAP2K2,MAPK1,MDM4,MMP16,NOTCH2,NRAS,PAK4,PDCD4,PDGFA,PDGFB,PDGFRB,PIK3CA,PRKCA,PRKCE,PTEN,PTGS2,RAF1,RDX,ROCK1,SHC1,SOCS1,SOS2,SOX4,STAT3,STMN1,TGFB2,THBS1,TNR,TP53,TP63,TRIM71,WNT3A,ZEB1,ZFPM2
Neurotrophin signaling pathway	predicted (union)	0.034	ABL1,AKT2,ARHGDIG,BAX,BCL2,BDNF,CALM1,CALM3,CAMK2A,CAMK2D,CAMK4,CRK,CRKL,FASLG,FOXO3,FRS2,GAB1,GSK3B,IRAK2,IRAK4,IRS1,KIDINS220,KRAS,MAP2K1,MAP2K2,MAP2K7,MAP3K1,MAP3K3,MAPK1,MAPK11,MAPK13,MAPK14,MAPK8,MAPK9,MAPKAPK2,NFKBIA,NGF,NGFR,NRAS,NTRK2,NTRK3,PIK3CA,PIK3CB,PIK3CG,PIK3R3,PTPN11,RAC1,RAF1,RAP1A,RPS6KA2,RPS6KA3,RPS6KA6,SH2B3,SHC1,SHC2,SHC3,SORT1,SOS2,TP53,TRAF6,YWHAE,ZNF274
Long-term potentiation	experimental (strong)	0.034	HRAS, KRAS,NRAS
Pancreatic cancer	experimental (any)	0.034	CCND1, CDK6,E2F2,EGFR,KRAS,NFKB1,PIK3R1,STAT3

	Epithelial cell signaling in Helicobacter pylori infection	experimental (strong)	0.036	CASP3, EGFR,PAK1
	NF-kappa B signaling pathway	experimental (any)	0.036	BCL2, CSNK2A1,CXCL8,LYN,NFKB1,PARP1,PLCG2,TAB2,TNFAIP3
	Regulation of actin cytoskeleton	experimental (any)	0.036	ACTB, CFL2,EGFR,F2R,HRAS,ITGA3,ITGB3,KRAS,NRAS,PAK1,PDGFB,PIK3R1,RDX,VAV2,VCL,WASL
	Axon guidance	experimental (strong)	0.037	HRAS, KRAS,NRAS,PAK1
	B cell receptor signaling pathway	experimental (any)	0.037	HRAS, KRAS,LYN,NFKB1,NRAS,PIK3R1,PLCG2,VAV2
	Neurotrophin signaling pathway	experimental (any)	0.042	BCL2, CAMK2D,HRAS,KRAS,MAP2K7,MAP3K1,MAPK11,NFKB1,NRAS,PIK3R1,PLCG2
	VEGF signaling pathway	experimental (strong)	0.043	HRAS, KRAS,NRAS
	Biosynthesis of amino acids	experimental (strong)	0.048	ARG2, PKM
	Dorso-ventral axis formation	experimental (strong)	0.048	EGFR, KRAS
	Chemokine signaling pathway	experimental (any)	0.048	CCR7, CXCL8,GNG5,HRAS,KRAS,LYN,NFKB1,NRAS,PAK1,PIK3R1,STAT2,STAT3,VAV2,WASL
	Axon guidance	predicted (union)	0.049	ABL1,ARHGEF12,CFL2,CXCL12,DCC,DPYSL5,EFNA5,EFNB3,EPHA2,EPHA3,EPHA4,EPHA5,EPHA6,EPHA7,EPHB1,EPHB2,GNAI3,GSK3B,KRAS,L1CAM,LIMK2,MAPK1,NCK1,NCK2,NFATC2,NFATC3,NRAS,NRP1,NTN1,NTN4,PAK1,PAK3,PAK4,PLXNA1,PLXNA2,PLXNA3,PLXNB1,PLXNC1,PPP3R1,RAC1,RASA1,RND1,ROBO1,ROCK1,ROCK2,SEMA3A,SEMA3D,SEMA3F,SEMA4C,SEMA4D,SEMA4F,SEMA4G,SEMA5A,SEMA6A,SLIT2,SLIT3,SRGAP1,SRGAP3,UNC5B,UNC5C,UNC5D
	FoxO signaling pathway	predicted (union)	0.049	AKT2,ATG12,ATM,CCNB1,CCND1,CCND2,CCNG2,CDKN1A,CDKN2B,CHUK,CSNK1E,EGF,EGFR,FASLG,FBXO32,FOXO1,FOXO3,G6PC,G6PC2,GADD45G,HOMER1,IGF1,IL10,IL6,INSR,IRS1,IRS2,IRS4,KLF2,KRAS,MAP2K1,MAP2K2,MAPK1,MAPK11,MAPK13,MAPK14,MAPK8,MAPK9,NLK,NRAS,PCK1,PIK3CA,PIK3CB,PIK3CG,PIK3R3,PRKAA2,PRKAB2,PTEN,RAF1,RAG1,S1PR1,SETD7,SKP2,SMAD2,SOD2,SOS2,STAT3,STK4,TGFB2,TGFBR1,TGFBR2,TNFSF10
	Prolactin signaling pathway	predicted (union)	0.049	AKT2,CCND1,CCND2,CISH,ESR1,ESR2,FOS,FOXO3,GSK3B,JAK2,KRAS,LHCGR,MAP2K1,MAP2K2,MAPK1,MAPK11,MAPK13,MAPK14,MAPK8,MAPK9,NRAS,PIK3CA,PIK3CB,PIK3CG,PIK3R3,PRLR,RAF1,SHC1,SHC2,SHC3,SLC2A2,SOCS1,SOCS4,SOCS5,SOS2,STAT3,TNFRSF11A
**hsa-let-7d-5p**	MicroRNAs in cancer	experimental (any)	0.002	CCND1,CDKN1A,DICER1,HMGA2,IGF2BP1,MDM4,MYC,PDGFA,PDGFB,PLCG2,RDX,SOCS1,THBS1,TRIM71,ZEB2
	FoxO signaling pathway	predicted (intersection)	0.013	AKT2,ATG12,ATM,CCNB1,CCNG2,CDKN1A,CHUK,EGF,EGFR,FASLG,FBXO32,FOXO1,GADD45G,IGF1,IL10,INSR,IRS1,IRS2,KLF2,MAPK1,MAPK11,MAPK8,MAPK9,NLK,NRAS,PIK3CA,PIK3CB,PRKAA2,PRKAB2,RAF1,SETD7,SKP2,SMAD2,SOD2,STAT3,STK4,TGFBR1,TGFBR2,TNFSF10
	MicroRNAs in cancer	predicted (intersection)	0.013	ABCC1,APC2,ATM,BAK1,BCL2,BCL2L2,BMF,CASP3,CDC25A,CDC25B,CDK6,CDKN1A,DNMT3A,E2F2,EGFR,FZD3,GLS,HMGA2,IGF2BP1,IRS1,IRS2,ITGB3,MAPK1,MDM4,MMP16,NOTCH2,NRAS,PDCD4,PDGFA,PDGFB,PIK3CA,PRKCE,RAF1,RDX,ROCK1,SHC1,SOCS1,STAT3,THBS1,TNR,TP53,TP63,TRIM71
**hsa-let-7c-5p**	MicroRNAs in cancer	experimental (any)	1.78e-8	CASP3,CCND1,CCNG1,CDC25A,CDKN1A,CRK,DNMT1,EZH2,HMGA2,IGF2BP1,ITGB3,MDM4,MTOR,MYC,NOTCH2,NRAS,PAK4,PDGFB,PLCG2,RDX,SIRT1,SOCS1,STAT3,THBS1,TRIM71
	MicroRNAs in cancer	experimental (strong)	1.79e-4	CASP3, CDC25A,HMGA2,ITGB3,MTOR,MYC,NRAS,STAT3,TRIM71
	FoxO signaling pathway	experimental (strong)	0.003	IGF1R, IL10,IL6,NRAS,STAT3,TGFBR1
	Pathways in cancer	experimental (strong)	0.003	BCL2L1, CASP3,IGF1R,IL6,MTOR,MYC,NRAS,STAT3,TGFBR1
	Transcriptional misregulation in cancer	experimental (strong)	0.003	BCL2L1, CEBPB,HMGA2,IGF1R,IL6,MYC
	FoxO signaling pathway	experimental (any)	0.003	ATG12, CCNB2,CCND1,CDKN1A,FOXO3,IGF1R,IL10,IL6,NRAS,SIRT1,SOD2,STAT3,STK4,TGFBR1
	p53 signaling pathway	experimental (any)	0.003	CASP3, CCNB2,CCND1,CCNG1,CDKN1A,MDM4,PMAIP1,RRM2,THBS1,TNFRSF10B
	Hepatitis B	experimental (strong)	0.005	CASP3, IL6,MYC,NRAS,STAT3,TGFBR1
	Jak-STAT signaling pathway	experimental (strong)	0.005	BCL2L1, IL10,IL6,MYC,STAT3
	Proteoglycans in cancer	experimental (strong)	0.005	CASP3, IGF1R,ITGB3,MTOR,MYC,NRAS,STAT3
	Neurotrophin signaling pathway	predicted (union)	0.006	ABL1,AKT2,ARHGDIB,ARHGDIG,BAX,BCL2,BDNF,CALM1,CALM3,CAMK2A,CAMK2D,CAMK4,CRK,CRKL,FASLG,FOXO3,FRS2,GAB1,GSK3B,IRAK2,IRAK4,IRS1,KIDINS220,KRAS,MAP2K1,MAP2K2,MAP2K7,MAP3K1,MAP3K3,MAPK1,MAPK11,MAPK13,MAPK14,MAPK8,MAPK9,MAPKAPK2,NFKBIA,NFKBIB,NGF,NGFR,NRAS,NTRK2,NTRK3,PIK3CA,PIK3CB,PIK3CG,PIK3R3,PTPN11,RAC1,RAF1,RAP1A,RAPGEF1,RPS6KA2,RPS6KA3,RPS6KA6,SH2B3,SHC1,SHC2,SHC3,SORT1,SOS2,TP53,TP73,TRAF6,YWHAE,ZNF274
	Acute myeloid leukemia	experimental (strong)	0.007	MTOR, MYC,NRAS,STAT3

PI3K-Akt signaling pathway	experimental (strong)	0.009	BCL2L1, IGF1R,IL6,ITGB3,MTOR,MYC,NRAS
Proteoglycans in cancer	experimental (any)	0.014	ACTB,CASP3,CBL,CCND1,CDKN1A,FLNB,FZD8,FZD9,IGF1R,ITGB3,MTOR,MYC,NRAS,PLCG2,PTK2,RDX,STAT3,THBS1
ErbB signaling pathway	experimental (any)	0.015	CBL, CDKN1A,CRK,MAP2K7,MTOR,MYC,NRAS,PAK4,PLCG2,PTK2
Jak-STAT signaling pathway	experimental (any)	0.015	BCL2L1, CBL,CCND1,IFNLR1,IL10,IL6,IL6R,MYC,SOCS1,SOCS7,STAT2,STAT3
Pathways in cancer	experimental (any)	0.015	BCL2L1,CASP3,CBL,CCND1,CDKN1A,CRK,CXCL8,DVL3,FZD8,FZD9,IGF1R,IL6,ITGA3,MTOR,MYC,NRAS,PDGFB,PLCG2,PTK2,RARB,STAT3,STK4,TGFBR1
Chronic myeloid leukemia	experimental (strong)	0.018	BCL2L1, MYC,NRAS,TGFBR1
Thyroid hormone signaling pathway	experimental (strong)	0.022	ITGB3, MTOR,MYC,NRAS
HIF-1 signaling pathway	experimental (strong)	0.024	IGF1R, IL6,MTOR,STAT3
FoxO signaling pathway	predicted (intersection)	0.025	AKT2,ATG12,ATM,CCNB1,CCND2,CCNG2,CDKN1A,CHUK,EGF,EGFR,FASLG,FBXO32,FOXO1,GADD45G,HOMER1,IGF1,IL10,INSR,IRS1,IRS2,KLF2,MAPK1,MAPK11,MAPK8,MAPK9,NLK,NRAS,PIK3CA,PRKAA2,PRKAB2,SETD7,SKP2,SMAD2,STAT3,STK4,TGFBR1,TGFBR2,TNFSF10
MicroRNAs in cancer	predicted (intersection)	0.025	ABCC1,APC2,ATM,BAK1,BCL2,BCL2L2,CASP3,CCND2,CD44,CDC25A,CDC25B,CDK6,CDKN1A,DNMT3A,E2F2,EGFR,FZD3,GLS,HMGA2,IGF2BP1,IRS1,IRS2,ITGB3,MAPK1,MDM4,MMP16,NOTCH2,NRAS,PDCD4,PDGFA,PDGFB,PIK3CA,PRKCE,RDX,ROCK1,SHC1,SOCS1,STAT3,THBS1,TNR,TP53,TP63,TRIM71
Inflammatory bowel disease (IBD)	experimental (strong)	0.027	IL10, IL6,STAT3
Toxoplasmosis	experimental (strong)	0.027	BCL2L1, CASP3,IL10,STAT3
Tuberculosis	experimental (strong)	0.027	CASP3, CEBPB,IL10,IL6
Arrhythmogenic right ventricular cardiomyopathy (ARVC)	experimental (any)	0.027	ACTB, ACTN4,ATP2A2,CACNG8,ITGA10,ITGA3,ITGB3,RYR2
Bladder cancer	experimental (any)	0.027	CCND1, CDKN1A,CXCL8,MYC,NRAS,THBS1
Transcriptional misregulation in cancer	experimental (any)	0.027	BCL2L1, CCNT2,CDKN1A,CEBPB,CXCL8,DOT1L,EWSR1,HMGA2,IGF1R,IL6,MEF2C,MYC,PBX3,PTK2
Pertussis	experimental (strong)	0.030	CASP3, IL10,IL6
African trypanosomiasis	experimental (strong)	0.033	IL10, IL6
Amoebiasis	experimental (strong)	0.033	CASP3, IL10,IL6
Apoptosis	experimental (strong)	0.033	BCL2L1, CASP3,TNFRSF10B
Colorectal cancer	experimental (strong)	0.033	CASP3, MYC,TGFBR1
Glioma	experimental (strong)	0.033	IGF1R, MTOR,NRAS
HTLV-I infection	experimental (strong)	0.033	BCL2L1, IL6,MYC,NRAS,TGFBR1
Intestinal immune network for IgA production	experimental (strong)	0.033	IL10, IL6
MAPK signaling pathway	experimental (strong)	0.033	CASP3, MAP4K3,MYC,NRAS,TGFBR1
Natural killer cell mediated cytotoxicity	experimental (strong)	0.033	CASP3, NRAS,TNFRSF10B
Chronic myeloid leukemia	experimental (any)	0.034	BCL2L1, CBL,CCND1,CDKN1A,CRK,MYC,NRAS,TGFBR1
Hypertrophic cardiomyopathy (HCM)	experimental (any)	0.034	ACTB, ATP2A2,CACNG8,IL6,ITGA10,ITGA3,ITGB3,RYR2
Notch signaling pathway	experimental (any)	0.034	DTX3L, DVL3,HES5,NOTCH2,NUMB,SNW1

	PI3K-Akt signaling pathway	experimental (any)	0.034	BCL2L1,CCND1,CDKN1A,FOXO3,GNG5,IGF1R,IL6,IL6R,ITGA10,ITGA3,ITGB3,MTOR,MYC,NRAS,PDGFB,PPP2R2A,PPP2R5E,PTK2,THBS1,YWHAZ
	Thyroid hormone signaling pathway	experimental (any)	0.037	ACTB, ATP2A2,CCND1,ITGB3,MTOR,MYC,NCOA3,NOTCH2,NRAS,PLCG2
	Cytokine-cytokine receptor interaction	experimental (strong)	0.041	IL10, IL6,TGFBR1,TNFRSF10B
	Pancreatic cancer	experimental (strong)	0.041	BCL2L1, STAT3,TGFBR1
	Thyroid cancer	experimental (strong)	0.044	MYC, NRAS
	Focal adhesion	experimental (any)	0.045	ACTB, ACTN4,CCND1,CRK,FLNB,IGF1R,ITGA10,ITGA3,ITGB3,PAK4,PDGFB,PTK2,THBS1,VCL
	Hepatitis B	experimental (any)	0.045	CASP3, CCND1,CDKN1A,CXCL8,IL6,MYC,NRAS,STAT2,STAT3,TGFBR1,YWHAZ
	TGF-beta signaling pathway	experimental (any)	0.045	ACVR1B, E2F5,ID1,LEFTY1,MYC,SMAD6,TGFBR1,THBS1
	Vibrio cholerae infection	experimental (any)	0.045	ACTB, ATP6V1F,ATP6V1G1,ERO1A,PLCG2,SLC12A2
	Glioma	experimental (any)	0.046	CCND1, CDKN1A,IGF1R,MTOR,NRAS,PDGFB,PLCG2
	HIF-1 signaling pathway	experimental (any)	0.046	CDKN1A, EDN1,IGF1R,IL6,IL6R,LDHA,MTOR,PLCG2,STAT3
	ErbB signaling pathway	experimental (strong)	0.050	MTOR, MYC,NRAS
**hsa-miR-376c-3p**	Prostate cancer	experimental (any)	4.51e-4	BCL2, GRB2,IGF1R,MDM2,PDPK1,TGFA
	Proteoglycans in cancer	experimental (any)	7.96e-4	ANK1, FRS2,FZD6,GRB2,IGF1R,MDM2,PDPK1,WNT10B
	FoxO signaling pathway	experimental (any)	0.001	ATM, GRB2,IGF1R,MDM2,PDPK1,TGFBR1
	Pathways in cancer	experimental (any)	0.001	BCL2, DAPK1,FZD6,GRB2,IGF1R,MDM2,TGFA,TGFBR1,WNT10B
	Ascorbate and aldarate metabolism	experimental (strong)	0.002	UGT2B15, UGT2B17
	Drug metabolism - other enzymes	experimental (strong)	0.002	UGT2B15, UGT2B17
	Pentose and glucuronate interconversions	experimental (strong)	0.002	UGT2B15, UGT2B17
	Porphyrin and chlorophyll metabolism	experimental (strong)	0.002	UGT2B15, UGT2B17
	Prostate cancer	experimental (strong)	0.002	BCL2, GRB2,IGF1R,TGFA
	Starch and sucrose metabolism	experimental (strong)	0.002	UGT2B15, UGT2B17
	Glioma	experimental (strong)	0.003	GRB2, IGF1R,TGFA
	Metabolism of xenobiotics by cytochrome P450	experimental (strong)	0.003	UGT2B15, UGT2B17
	Retinol metabolism	experimental (strong)	0.003	UGT2B15, UGT2B17
	Steroid hormone biosynthesis	experimental (strong)	0.003	UGT2B15, UGT2B17
	Glioma	experimental (any)	0.003	GRB2, IGF1R,MDM2,TGFA
	MicroRNAs in cancer	experimental (any)	0.003	ATM, BCL2,BMI1,GRB2,MDM2,RPS6KA5
	Neurotrophin signaling pathway	experimental (any)	0.003	BCL2, FRS2,GRB2,PDPK1,RPS6KA5
	Transcriptional misregulation in cancer	experimental (any)	0.003	ATM, BMI1,CDK14,IGF1R,MDM2,RUNX2
	p53 signaling pathway	experimental (any)	0.003	ATM, CHEK2,MDM2,TNFRSF10B

Drug metabolism - cytochrome P450	experimental (strong)	0.004	UGT2B15, UGT2B17
Pathways in cancer	experimental (strong)	0.004	BCL2, GRB2,IGF1R,TGFA,TGFBR1
Chemical carcinogenesis	experimental (strong)	0.005	UGT2B15, UGT2B17
Apoptosis	experimental (any)	0.006	ATM, BCL2,DFFA,TNFRSF10B
Bladder cancer	experimental (any)	0.007	DAPK1, MDM2,RPS6KA5
Retinol metabolism	experimental (any)	0.010	ADH4, UGT2B15,UGT2B17
FoxO signaling pathway	experimental (strong)	0.013	GRB2, IGF1R,TGFBR1
Transcriptional misregulation in cancer	experimental (strong)	0.013	BMI1, IGF1R,RUNX2
Drug metabolism - cytochrome P450	experimental (any)	0.013	ADH4, UGT2B15,UGT2B17
Ascorbate and aldarate metabolism	experimental (any)	0.015	UGT2B15, UGT2B17
Cell cycle	experimental (any)	0.015	ATM, CHEK2,MDM2,ORC4
Chemical carcinogenesis	experimental (any)	0.015	ADH4, UGT2B15,UGT2B17
Endocytosis	experimental (any)	0.015	ARF6, IGF1R,MDM2,NEDD4L,TGFBR1
Metabolism of xenobiotics by cytochrome P450	experimental (any)	0.015	ADH4, UGT2B15,UGT2B17
Non-small cell lung cancer	experimental (any)	0.015	GRB2, PDPK1,TGFA
Hepatitis B	experimental (strong)	0.017	BCL2, GRB2,TGFBR1
Focal adhesion	experimental (strong)	0.020	BCL2, GRB2,IGF1R
Chronic myeloid leukemia	experimental (any)	0.024	GRB2, MDM2,TGFBR1
Pentose and glucuronate interconversions	experimental (any)	0.024	UGT2B15, UGT2B17
Non-small cell lung cancer	experimental (strong)	0.026	GRB2, TGFA
Adherens junction	experimental (strong)	0.027	IGF1R, TGFBR1
Colorectal cancer	experimental (strong)	0.027	BCL2, TGFBR1
MicroRNAs in cancer	experimental (strong)	0.027	BCL2, BMI1,GRB2
TGF-beta signaling pathway	experimental (strong)	0.029	ACVR1C, TGFBR1
Pancreatic cancer	experimental (strong)	0.030	TGFA, TGFBR1
Renal cell carcinoma	experimental (strong)	0.030	GRB2, TGFA
Chronic myeloid leukemia	experimental (strong)	0.034	GRB2, TGFBR1
ErbB signaling pathway	experimental (strong)	0.034	GRB2, TGFA
HTLV-I infection	experimental (any)	0.034	ATM, CHEK2,FZD6,TGFBR1,WNT10B
ErbB signaling pathway	predicted (union)	0.036	ABL2,AKT2,CAMK2A,CAMK2D,CBL,CDKN1A,CRKL,EGF,EGFR,EIF4EBP1,ELK1,ERBB2,EREG,GSK3B,HBEGF,JUN,KRAS,MAP2K2,MAP2K4,MAPK1,MAPK10,NCK1,NCK2,NRAS,NRG1,NRG2,NRG3,NRG4,PAK1,PAK2,PAK3,PAK4,PIK3CA,PIK3CG,PIK3R1,PIK3R3,PLCG1,RPS6KB1,SHC1,SHC3,SHC4,SRC,STAT5A,TGFA

	MicroRNAs in cancer	predicted (union)	0.036	ATM,BAK1,BCL2,BCL2L11,BMF,BMI1,BMPR2,CASP3,CCND1,CCND2,CCNE2,CD44,CDK6,CDKN1A,COMMD3,BMI1,CREBBP,CRKL,DICER1,DNMT1,DNMT3A,E2F3,EFNA3,EGFR,ERBB2,FZD3,GLS,HDAC1,HMGA2,HNRNPK,HOXD10,IGF2BP1,IRS1,IRS2,ITGB3,KRAS,MAP2K2,MAPK1,MCL1,MDM4,MET,MMP16,NOTCH1,NOTCH2,NOTCH4,NRAS,PAK4,PDGFA,PDGFRA,PDGFRB,PIK3CA,PLCG1,PRKCE,PTEN,PTGS2,RDX,ROCK1,SHC1,SHC4,SLC7A1,SOX4,TGFB2,THBS1,TIMP3,TNN,TNR,TP63,UBE2I,VEGFA,WNT3,ZEB1
	Proteoglycans in cancer	predicted (union)	0.036	AKT2,ANK1,ANK2,ARHGEF12,CAMK2A,CAMK2D,CASP3,CBL,CCND1,CD44,CDKN1A,COL21A1,CTTN,DCN,DROSHA,EGFR,EIF4B,ELK1,ERBB2,ESR1,FAS,FGF10,FGF12,FGF14,FGF2,FGF23,FGF5,FGF9,FGFR1,FN1,FRS2,FZD1,FZD3,FZD4,FZD5,FZD6,FZD7,GPC3,HBEGF,HGF,HIF1A,HOXD10,HPSE,HPSE2,IGF1,IQGAP1,ITGA2,ITGAV,ITGB3,ITPR1,ITPR2,KRAS,LUM,MAP2K2,MAPK1,MAPK13,MAPK14,MET,MMP2,NRAS,NUDT16L1,PAK1,PDPK1,PIK3CA,PIK3CG,PIK3R1,PIK3R3,PLCE1,PLCG1,PPP1CB,PPP1CC,PPP1R12A,PPP1R12B,PRKACB,PTCH1,RAC1,RDX,ROCK1,RPS6,RPS6KB1,RRAS2,SDC2,SDC4,SMAD2,SMO,SRC,TGFB2,THBS1,TIAM1,TIMP3,TLR2,VEGFA,WNT10B,WNT2,WNT2B,WNT3,WNT9A
	Drug metabolism - other enzymes	experimental (any)	0.037	UGT2B15, UGT2B17
	Porphyrin and chlorophyll metabolism	experimental (any)	0.037	UGT2B15, UGT2B17
	HIF-1 signaling pathway	experimental (strong)	0.040	BCL2, IGF1R
	PI3K-Akt signaling pathway	experimental (strong)	0.040	BCL2, GRB2,IGF1R
	Tyrosine metabolism	experimental (any)	0.040	ADH4, TAT
	Endocytosis	experimental (strong)	0.041	IGF1R, TGFBR1
	Aldosterone-regulated sodium reabsorption	experimental (any)	0.041	NEDD4L, PDPK1
	Dopaminergic synapse	predicted (union)	0.044	ADCY5,AKT2,ARNTL,ATF2,CACNA1C,CAMK2A,CAMK2D,CREB1,CREB3L2,CREB5,GNAL,GNAO1,GNAQ,GNAS,GNB4,GNB5,GNG10,GNG11,GNG12,GNG2,GNG4,GNG7,GNGT1,GRIA1,GRIA2,GRIA4,GRIN2A,GRIN2B,GSK3B,ITPR1,ITPR2,KCNJ3,KCNJ6,KCNJ9,KIF5A,KIF5C,MAOA,MAPK10,MAPK13,MAPK14,PLCB1,PLCB4,PPP1CB,PPP1CC,PPP2CA,PPP2CB,PPP2R1A,PPP2R2A,PPP2R2B,PPP2R2C,PPP2R3A,PPP2R5A,PPP2R5C,PPP2R5E,PPP3CB,PRKACB,SCN1A,SLC18A1,SLC18A2,SLC6A3
	Prostate cancer	predicted (union)	0.044	AKT2,AR,BCL2,CASP9,CCND1,CCNE2,CDKN1A,CHUK,CREB1,CREB3L2,CREB5,CREBBP,E2F3,EGF,EGFR,ERBB2,FGFR1,GSK3B,HSP90AA1,IGF1,KLK3,KRAS,MAP2K2,MAPK1,NFKBIA,NKX3-1,NRAS,PDGFA,PDGFC,PDGFD,PDGFRA,PDGFRB,PDPK1,PIK3CA,PIK3CG,PIK3R1,PIK3R3,PTEN,RB1,TCF7L1,TCF7L2,TGFA
	Renal cell carcinoma	predicted (union)	0.044	AKT2,ARNT,ARNT2,CREBBP,CRKL,EGLN1,EGLN3,EPAS1,FH,FLCN,HGF,HIF1A,JUN,KRAS,MAP2K2,MAPK1,MET,NRAS,PAK1,PAK2,PAK3,PAK4,PIK3CA,PIK3CG,PIK3R1,PIK3R3,RAC1,RAP1A,SLC2A1,TGFA,TGFB2,VEGFA,VHL
	Neurotrophin signaling pathway	experimental (strong)	0.045	BCL2, GRB2
	Osteoclast differentiation	experimental (strong)	0.045	GRB2, TGFBR1
	Starch and sucrose metabolism	experimental (any)	0.048	UGT2B15, UGT2B17
	Focal adhesion	experimental (any)	0.049	BCL2, GRB2,IGF1R,PDPK1
	Steroid hormone biosynthesis	experimental (any)	0.049	UGT2B15, UGT2B17
**hsa-miR-377-3p**	Wnt signaling pathway	predicted (union)	0.001	AXIN1,BAMBI,BTRC,CAMK2A,CAMK2B,CAMK2D,CCND1,CCND2,CCND3,CSNK1A1,CSNK2A1,CTBP1,CTBP2,CTNNBIP1,CUL1,CXXC4,DAAM1,DAAM2,DKK1,DKK2,DVL2,DVL3,EP300,FBXW11,FZD1,FZD10,FZD2,FZD3,FZD4,FZD5,FZD6,FZD7,GPC4,GSK3B,LEF1,LRP6,MAP3K7,MAPK10,MAPK8,MAPK9,MMP7,NFATC1,NFATC2,NFATC3,NFATC4,NKD1,NLK,PLCB1,PLCB2,PLCB3,PLCB4,PPARD,PPP3CC,PPP3R1,PPP3R2,PRICKLE2,PRKACB,PRKCA,PRKX,PSEN1,RAC1,RBX1,ROCK2,RUVBL1,SENP2,SFRP1,SKP1,SMAD3,SOX17,TBL1X,TBL1XR1,TBL1Y,TCF7L1,TCF7L2,VANGL1,VANGL2,WNT10B,WNT2B,WNT7A,WNT9A,WNT9B
	Glioma	experimental (strong)	0.002	E2F3, PTEN,TP53
	Huntington's disease	experimental (strong)	0.002	SOD1, SOD2,TP53
	Melanoma	experimental (strong)	0.002	E2F3, PTEN,TP53
	MicroRNAs in cancer	experimental (strong)	0.002	DNMT1, E2F3,PTEN,TP53
	Peroxisome	experimental (strong)	0.002	SOD1, SOD2
	Small cell lung cancer	experimental (strong)	0.002	E2F3, PTEN,TP53
	Prostate cancer	experimental (strong)	0.003	E2F3, PTEN,TP53

Wnt signaling pathway	predicted (intersection)	0.005	AXIN1,BTRC,CAMK2A,CAMK2B,CAMK2D,CCND2,CCND3,CSNK1A1,CSNK2A1,CTBP2,CTNNBIP1,CUL1,CXXC4,DAAM1,DVL2,DVL3,EP300,FZD2,FZD4,FZD6,GSK3B,LEF1,LRP6,MAP3K7,MAPK9,NFATC1,NKD1,PLCB1,PLCB2,PLCB4,PPARD,PPP3CC,PPP3R1,PPP3R2,PRICKLE2,PRKACB,PRKCA,PRKX,PSEN1,RAC1,RBX1,ROCK2,SENP2,SMAD3,TBL1X,TBL1XR1,TBL1Y,TCF7L2,VANGL1,VANGL2,WNT10B, WNT7A
Amyotrophic lateral sclerosis (ALS)	experimental (strong)	0.007	SOD1, TP53
Hepatitis B	experimental (strong)	0.007	E2F3, PTEN,TP53
Axon guidance	predicted (union)	0.008	ABLIM1,ABLIM2,ABLIM3,ARHGEF12,CDC42,CFL2,CXCL12,DCC,DPYSL2,DPYSL5,EFNA1,EFNA5,EFNB2,EPHA2,EPHA3,EPHA4,EPHA5,EPHA6,EPHA7,EPHA8,EPHB2,EPHB3,GNAI3,GSK3B,KRAS,LIMK2,MAPK1,MAPK3,MET,NCK1,NFATC2,NFATC3,NFATC4,NRAS,NRP1,NTN4,NTNG1,NTNG2,PAK1,PAK2,PAK3,PAK4,PLXNA2,PLXNA3,PLXNB1,PLXNC1,PPP3CC,PPP3R1,PPP3R2,RAC1,RASA1,ROBO2,ROCK2,SEMA3A,SEMA3C,SEMA3D,SEMA3G,SEMA4C,SEMA4D,SEMA4F,SEMA5A,SEMA6A,SEMA6D,SLIT1,SLIT2,SLIT3,SRGAP1,SRGAP3,UNC5B,UNC5C
Bladder cancer	experimental (strong)	0.010	E2F3, TP53
Endometrial cancer	experimental (strong)	0.012	PTEN, TP53
Non-small cell lung cancer	experimental (strong)	0.013	E2F3, TP53
p53 signaling pathway	experimental (strong)	0.013	PTEN, TP53
Amyotrophic lateral sclerosis (ALS)	predicted (union)	0.017	APAF1,BAD,BAX,BCL2,BCL2L1,BID,CASP1,CASP3,CASP9,CCS,CYCS,DERL1,GRIA1,GRIA2,GRIN1,GRIN2A,GRIN2B,MAP2K3,MAP2K6,MAPK11,MAPK13,MAPK14,NOS1,PPP3CC,PPP3R1,PPP3R2,RAC1,SLC1A2,SOD1,TNFRSF1B,TOMM40,TOMM40L
Colorectal cancer	predicted (union)	0.017	AKT2,APPL1,ARAF,AXIN1,BAD,BAX,BCL2,BIRC5,CASP3,CASP9,CCND1,CYCS,DCC,GSK3B,KRAS,LEF1,MAPK1,MAPK10,MAPK3,MAPK8,MAPK9,MLH1,MSH2,MSH3,PIK3CA,PIK3CB,PIK3CG,PIK3R1,RAC1,SMAD2,SMAD3,TCF7L1,TCF7L2,TGFB2,TGFB3,TGFBR1,TGFBR2
Protein processing in endoplasmic reticulum	predicted (union)	0.017	AMFR,ATF6,ATF6B,ATXN3L,BAG2,BAX,BCL2,CANX,CKAP4,CUL1,DERL1,DERL2,DERL3,DNAJB1,DNAJB12,DNAJC10,DNAJC3,DNAJC5,DNAJC5B,EDEM3,EIF2AK2,EIF2AK3,EIF2S1,ERN1,ERO1A,ERO1B,ERP29,HSP90AA1,HSPA1L,HSPA4L,HSPA6,HSPH1,LMAN1,LMAN2,MAN1A1,MAN1A2,MAN1B1,MAN1C1,MAP2K7,MAPK10,MAPK8,MAPK9,MARCH6,MBTPS2,NPLOC4,OS9,PDIA4,PLAA,PPP1R15A,PREB,RBX1,RNF185,RPN1,RPN2,SAR1A,SAR1B,SEC23B,SEC31B,SEC61A1,SEC61G,SEC63,SKP1,SSR1,SSR3,STT3B,STUB1,SVIP,TRAF2,UBE2D1,UBE2D2,UBE2D3,UBE2D4,UBE2E2,UBE2E3,UBE2G1,UBE2G2,UBE2J1,UBE2J2,UBE4B,UBQLN3,UBQLN4,UGGT1,VCP,WFS1,YOD1
TGF-beta signaling pathway	predicted (union)	0.017	ACVR1B,ACVR2A,ACVR2B,BAMBI,BMP5,BMP6,BMP7,BMP8A,BMP8B,BMPR1A,BMPR1B,BMPR2,CDKN2B,CUL1,DCN,EP300,GDF5,GDF6,GDF7,ID4,INHBA,INHBE,LEFTY1,LEFTY2,MAPK1,MAPK3,NODAL,PITX2,PPP2CA,PPP2R1A,PPP2R1B,RBL1,RBX1,RPS6KB1,RPS6KB2,SKP1,SMAD2,SMAD3,SMAD5,SMAD7,SMURF1,SP1,TGFB2,TGFB3,TGFBR1,TGFBR2,THBS1
Pancreatic cancer	experimental (strong)	0.018	E2F3, TP53
Chronic myeloid leukemia	experimental (strong)	0.021	E2F3, TP53
Retrograde endocannabinoid signaling	predicted (union)	0.024	ADCY1,ADCY2,ADCY3,ADCY9,CACNA1B,CACNA1C,CNR1,FAAH,GABRA1,GABRA4,GABRB1,GABRB3,GABRE,GABRG1,GABRG3,GABRR2,GNAI3,GNAO1,GNAQ,GNB4,GNB5,GNG12,GNG13,GNG2,GNG4,GNG7,GNGT2,GRIA1,GRIA2,GRIA4,KCNJ3,KCNJ5,KCNJ6,KCNJ9,MAPK1,MAPK10,MAPK11,MAPK13,MAPK14,MAPK3,MAPK8,MAPK9,NAPEPLD,PLCB1,PLCB2,PLCB3,PLCB4,PRKACB,PRKCA,PRKX,PTGS2,SLC17A8
Glycosaminoglycan biosynthesis - chondroitin sulfate / dermatan sulfate	predicted (union)	0.025	B3GALT6,B3GAT3,B4GALT7,CHST11,CHST12,CHST14,CHST15,CHST3,CHST7,CHSY1,CHSY3,CSGALNACT1,CSGALNACT2,DSE,UST,XYLT1
mTOR signaling pathway	predicted (union)	0.025	AKT2,CAB39,CAB39L,DDIT4,EIF4B,EIF4E,EIF4EBP1,IGF1,IRS1,MAPK1,MAPK3,PDPK1,PIK3CA,PIK3CB,PIK3CG,PIK3R1,PRKAA1,PRKAA2,PRKCA,PTEN,RICTOR,RPS6,RPS6KA1,RPS6KA3,RPS6KA6,RPS6KB1,RPS6KB2,RPTOR,RRAGC,RRAGD,STRADA,TSC1,ULK1,ULK2,VEGFA
FoxO signaling pathway	predicted (union)	0.026	AGAP2,AKT2,ARAF,ATG12,ATM,BCL2L11,BNIP3,CCNB1,CCND1,CCND2,CCNG2,CDKN1B,CDKN2B,CDKN2D,CHUK,EGF,EGFR,EP300,FBXO32,FOXO1,FOXO3,G6PC,G6PC3,GABARAP,HOMER1,IGF1,IL7R,INSR,IRS1,KLF2,KRAS,MAPK1,MAPK10,MAPK11,MAPK13,MAPK14,MAPK3,MAPK8,MAPK9,NLK,NRAS,PDPK1,PIK3CA,PIK3CB,PIK3CG,PIK3R1,PRKAA1,PRKAA2,PRKAB2,PRKAG2,PTEN,RAG1,RBL2,S1PR1,S1PR4,SETD7,SGK1,SIRT1,SLC2A4,SMAD2,SMAD3,SOD2,TGFB2,TGFB3,TGFBR1,TGFBR2,USP7
Circadian entrainment	predicted (intersection)	0.027	ADCY1,ADCY2,ADCY3,ADCYAP1R1,CACNA1C,CAMK2A,CAMK2B,CAMK2D,GNAI3,GNAQ,GNB5,GNG12,GNG2,GNG4,GNG7,GRIA1,GRIA2,GRIA4,GRIN2A,GRIN2B,GUCY1A2,KCNJ3,KCNJ6,MAPK1,NOS1,NOS1AP,PER2,PER3,PLCB1,PLCB2,PLCB4,PRKACB,PRKCA,PRKG1,PRKX
ErbB signaling pathway	predicted (intersection)	0.027	AKT2,CAMK2A,CAMK2B,CAMK2D,CBL,CBLB,CDKN1B,EGF,EGFR,EIF4EBP1,ELK1,ERBB4,GSK3B,KRAS,MAPK1,MAPK9,NCK1,NRG1,NRG2,NRG3,PAK1,PAK2,PAK3,PAK4,PIK3CB,PIK3CG,PLCG1,PRKCA,RPS6KB1,SHC3,STAT5B,TGFA
Glutamatergic synapse	predicted (intersection)	0.027	ADCY1,ADCY2,ADCY3,CACNA1C,DLGAP1,GLS,GNAI3,GNAQ,GNB5,GNG12,GNG2,GNG4,GNG7,GRIA1,GRIA2,GRIA4,GRIK1,GRIK3,GRIN2A,GRIN2B,GRK3,GRM4,GRM6,HOMER1,KCNJ3,MAPK1,PLA2G4A,PLCB1,PLCB2,PLCB4,PPP3CC,PPP3R1,PPP3R2,PRKACB,PRKCA,PRKX,SLC17A8,SLC1A7,SLC38A1
Retrograde endocannabinoid signaling	predicted (intersection)	0.027	ADCY1,ADCY2,ADCY3,CACNA1B,CACNA1C,CNR1,GABRA4,GABRB1,GABRE,GABRG1,GABRR2,GNAI3,GNAQ,GNB5,GNG12,GNG2,GNG4,GNG7,GRIA1,GRIA2,GRIA4,KCNJ3,KCNJ6,MAPK1,MAPK13,MAPK14,MAPK9,PLCB1,PLCB2,PLCB4,PRKACB,PRKCA,PRKX,SLC17A8

mTOR signaling pathway	predicted (intersection)	0.027	AKT2,CAB39,CAB39L,EIF4B,EIF4E,EIF4EBP1,IGF1,MAPK1,PDPK1,PIK3CB,PIK3CG,PRKAA1,PRKAA2,PRKCA,PTEN,RICTOR,RPS6KA3,RPS6KA6,RPS6KB1,RRAGC,TSC1,ULK1,ULK2,VEGFA
Cell cycle	experimental (strong)	0.027	E2F3, TP53
ErbB signaling pathway	predicted (union)	0.028	AKT2,ARAF,BAD,CAMK2A,CAMK2B,CAMK2D,CBL,CBLB,CDKN1B,CRK,EGF,EGFR,EIF4EBP1,ELK1,ERBB2,ERBB4,EREG,GSK3B,KRAS,MAP2K7,MAPK1,MAPK10,MAPK3,MAPK8,MAPK9,NCK1,NRAS,NRG1,NRG2,NRG3,PAK1,PAK2,PAK3,PAK4,PIK3CA,PIK3CB,PIK3CG,PIK3R1,PLCG1,PRKCA,RPS6KB1,RPS6KB2,SHC3,SHC4,SRC,STAT5B,TGFA
Hippo signaling pathway	predicted (union)	0.028	ACTB,AFP,AMOT,AXIN1,BBC3,BIRC5,BMP5,BMP6,BMP7,BMP8A,BMP8B,BMPR1A,BMPR1B,BMPR2,BTRC,CCN2,CCND1,CCND2,CCND3,CRB1,CRB2,CTNNA2,DLG2,DVL2,DVL3,FBXW11,FGF1,FRMD1,FZD1,FZD10,FZD2,FZD3,FZD4,FZD5,FZD6,FZD7,GDF5,GDF6,GDF7,GLI2,GSK3B,LATS2,LEF1,LIMD1,MOB1A,MOB1B,MPP5,NF2,NKD1,PPP2CA,PPP2R1A,PPP2R1B,PPP2R2C,PRKCI,RASSF1,RASSF6,SAV1,SERPINE1,SMAD2,SMAD3,SMAD7,STK3,TCF7L1,TCF7L2,TGFB2,TGFB3,TGFBR1,TGFBR2,TP53BP2,TP73,WNT10B,WNT2B,WNT7A,WNT9A,WNT9B,WTIP,WWC1,YWHAG,YWHAZ
Pathways in cancer	predicted (union)	0.028	AKT2,APPL1,AR,ARAF,ARNT,ARNT2,AXIN1,BAD,BAX,BCL2,BCL2L1,BCR,BID,BIRC5,CASP3,CASP9,CBL,CBLB,CCDC6,CCND1,CCNE2,CDC42,CDK6,CDKN1B,CDKN2B,CHUK,CKS1B,COL4A1,COL4A4,COL4A6,CRK,CTBP1,CTBP2,CTNNA2,CYCS,DCC,DVL2,DVL3,E2F1,E2F3,EGF,EGFR,EGLN1,EGLN3,EP300,EPAS1,ERBB2,FADD,FGF1,FGF10,FGF12,FGF14,FGF2,FGF5,FGF9,FGFR1,FLT3LG,FOXO1,FZD1,FZD10,FZD2,FZD3,FZD4,FZD5,FZD6,FZD7,GLI2,GLI3,GSK3B,HDAC2,HGF,HHIP,HSP90AA1,IGF1,ITGA3,ITGAV,KIT,KLK3,KRAS,LAMA1,LAMA4,LAMA5,LAMB2,LAMC1,LAMC2,LAMC3,LEF1,MAPK1,MAPK10,MAPK3,MAPK8,MAPK9,MAX,MET,MITF,MLH1,MMP1,MSH2,MSH3,NFKBIA,NRAS,PDGFB,PDGFRA,PIAS2,PIK3CA,PIK3CB,PIK3CG,PIK3R1,PLCG1,PPARD,PRKCA,PTCH1,PTCH2,PTEN,PTGS2,RAC1,RAD51,RALA,RASSF1,RB1,RBX1,RET,RUNX1,RUNX1T1,RXRA,RXRG,SHH,SMAD2,SMAD3,STAT5B,SUFU,TCF7L1,TCF7L2,TFG,TGFA,TGFB2,TGFB3,TGFBR1,TGFBR2,TPM3,TRAF1,TRAF2,TRAF3,TRAF4,TRAF6,VEGFA,VHL,WNT10B,WNT2B,WNT7A,WNT9A,WNT9B,XIAP
MAPK signaling pathway	predicted (intersection)	0.033	AKT2,ARRB1,CACNA1B,CACNA1C,CACNA2D1,CACNA2D2,CACNB2,CACNB3,CACNB4,CDC42,CHUK,DUSP1,DUSP2,DUSP6,DUSP7,EGF,EGFR,ELK1,ELK4,FGF10,FGF12,FGF9,FGFR1,FLNC,GNG12,HSPA6,IL1R1,KRAS,LAMTOR3,MAP2K3,MAP2K6,MAP3K1,MAP3K13,MAP3K20,MAP3K7,MAP4K2,MAPK1,MAPK13,MAPK14,MAPK9,MAPKAPK3,MAPKAPK5,MAPT,MAX,MEF2C,MRAS,NFATC1,NTRK2,PAK1,PAK2,PDGFRA,PLA2G4A,PPM1A,PPM1B,PPP3CC,PPP3R1,PPP3R2,PRKACB,PRKCA,PRKX,RAC1,RAP1A,RASA1,RASA2,RASGRF2,RPS6KA3,RPS6KA6,RRAS,SRF,STK3,TAB1,TAB2,TAOK1,TGFBR2,TRAF6
TGF-beta signaling pathway	predicted (intersection)	0.033	ACVR2A,ACVR2B,BMP6,BMP7,BMP8B,BMPR1A,BMPR2,CDKN2B,CUL1,DCN,EP300,GDF6,GDF7,INHBA,LEFTY2,MAPK1,NODAL,PITX2,PPP2R1A,PPP2R1B,RBL1,RBX1,RPS6KB1,SMAD2,SMAD3,SMAD7,SMURF1,SP1,TGFBR2,THBS1
Pathways in cancer	experimental (strong)	0.034	E2F3, PTEN,TP53
Long-term potentiation	predicted (intersection)	0.036	ADCY1,CACNA1C,CAMK2A,CAMK2B,CAMK2D,CAMK4,EP300,GNAQ,GRIA1,GRIA2,GRIN2A,GRIN2B,KRAS,MAPK1,PLCB1,PLCB2,PLCB4,PPP3CC,PPP3R1,PPP3R2,PRKACB,PRKCA,PRKX,RAP1A,RPS6KA3,RPS6KA6
FoxO signaling pathway	experimental (strong)	0.037	PTEN, SOD2
Focal adhesion	predicted (union)	0.038	ACTB,ACTN1,ACTN4,AKT2,ARHGAP5,BAD,BCAR1,BCL2,CAV2,CCND1,CCND2,CCND3,CDC42,COL1A2,COL2A1,COL4A1,COL4A4,COL4A6,COL5A1,COL5A2,CRK,DIAPH1,DOCK1,EGF,EGFR,ELK1,ERBB2,FLNC,FLT4,GSK3B,HGF,IGF1,ITGA1,ITGA11,ITGA3,ITGA4,ITGA8,ITGA9,ITGAV,ITGB3,ITGB5,ITGB6,ITGB8,KDR,LAMA1,LAMA4,LAMA5,LAMB2,LAMC1,LAMC2,LAMC3,MAPK1,MAPK10,MAPK3,MAPK8,MAPK9,MET,MYL10,MYLK3,PAK1,PAK2,PAK3,PAK4,PARVA,PARVB,PARVG,PDGFB,PDGFC,PDGFRA,PDPK1,PIK3CA,PIK3CB,PIK3CG,PIK3R1,PPP1R12A,PPP1R12B,PPP1R12C,PRKCA,PTEN,PXN,RAC1,RAP1A,RAPGEF1,RASGRF1,ROCK2,SHC3,SHC4,SPP1,SRC,THBS1,THBS2,TLN1,TLN2,TNN,TNR,VAV3,VCL,VEGFA,XIAP
MicroRNAs in cancer	predicted (union)	0.038	ATM,BCL2,BCL2L11,BCL2L2,BMF,BMPR2,CASP3,CCND1,CCND2,CCNE2,CDC25A,CDC25B,CDC25C,CDCA5,CDK6,CDKN1B,CRK,CYP24A1,DDIT4,DNMT1,DNMT3A,DNMT3B,E2F1,E2F3,EGFR,EP300,ERBB2,EZR,FOXP1,FZD3,GLS,HDAC4,HMGA2,HMOX1,HNRNPK,HOXD10,IRS1,ITGB3,KIF23,KRAS,MAPK1,MDM4,MET,MMP16,NOTCH2,NRAS,PAK4,PDGFB,PDGFRA,PIK3CA,PIM1,PLAU,PLCG1,PRKCA,PRKCE,PTEN,PTGS2,RASSF1,RDX,RPS6KA5,RPTOR,SHC4,SIRT1,SLC7A1,SOX4,TGFB2,THBS1,TIMP3,TNN,TNR,TP63,TRIM71,UBE2I,VEGFA,ZEB1
Proteoglycans in cancer	predicted (union)	0.038	ACTB,AKT2,ANK3,ARAF,ARHGEF1,ARHGEF12,CAMK2A,CAMK2B,CAMK2D,CASP3,CAV2,CBL,CBLB,CCND1,CDC42,CTSL,CTTN,DCN,DDX5,EGFR,EIF4B,ELK1,ERBB2,ERBB4,ESR1,EZR,FGF1,FGF10,FGF12,FGF14,FGF2,FGF5,FGF9,FGFR1,FLNC,FRS2,FZD1,FZD10,FZD2,FZD3,FZD4,FZD5,FZD6,FZD7,HCLS1,HGF,HOXD10,HPSE,HPSE2,IGF1,ITGAV,ITGB3,ITGB5,KDR,KRAS,LUM,MAPK1,MAPK11,MAPK13,MAPK14,MAPK3,MET,MRAS,NRAS,NUDT16L1,PAK1,PDPK1,PIK3CA,PIK3CB,PIK3CG,PIK3R1,PLAU,PLAUR,PLCE1,PLCG1,PPP1R12A,PPP1R12B,PPP1R12C,PRKACB,PRKCA,PRKX,PTCH1,PTPN11,PXN,RAC1,RDX,ROCK2,RPS6,RPS6KB1,RPS6KB2,RRAS,SDC1,SDC2,SLC9A1,SMAD2,SRC,TGFB2,THBS1,TIAM1,TIMP3,VEGFA,WNT10B,WNT2B,WNT7A,WNT9A,WNT9B
Rap1 signaling pathway	predicted (union)	0.038	ACTB,ADCY1,ADCY2,ADCY3,ADCY9,ADORA2A,ADORA2B,AFDN,AKT2,ARAP2,BCAR1,CDC42,CNR1,CRK,CSF1,DOCK4,EFNA1,EFNA5,EGF,EGFR,EPHA2,F2R,FGF1,FGF10,FGF12,FGF14,FGF2,FGF5,FGF9,FGFR1,FLT4,FPR1,GNAI3,GNAO1,GNAQ,GNAS,GRIN1,GRIN2A,GRIN2B,HGF,IGF1,INSR,ITGB3,KDR,KIT,KRAS,LAT,LPAR1,LPAR4,MAGI1,MAGI2,MAGI3,MAP2K3,MAP2K6,MAPK1,MAPK11,MAPK13,MAPK14,MAPK3,MET,MRAS,NGFR,NRAS,P2RY1,PDGFB,PDGFC,PDGFRA,PFN1,PFN2,PFN4,PIK3CA,PIK3CB,PIK3CG,PIK3R1,PLCB1,PLCB2,PLCB3,PLCB4,PLCE1,PLCG1,PRKCA,PRKCI,PRKD1,RAC1,RALA,RAP1A,RAP1GAP,RAPGEF1,RAPGEF3,RAPGEF6,RASGRP3,RRAS,SIPA1L1,SIPA1L3,SRC,TEK,THBS1,TIAM1,TLN1,TLN2,VEGFA
SNARE interactions in vesicular transport	predicted (union)	0.038	BET1,BET1L,GOSR1,GOSR2,SNAP25,SNAP29,SNAP47,STX11,STX16,STX17,STX1A,STX1B,STX2,STX4,STX6,STX7,VAMP1,VAMP2,VAMP3,VAMP4,VAMP5,VTI1A,VTI1B

	Small cell lung cancer	predicted (union)	0.038	AKT2,APAF1,BCL2,BCL2L1,CASP9,CCND1,CCNE2,CDK6,CDKN1B,CDKN2B,CHUK,CKS1B,COL4A1,COL4A4,COL4A6,CYCS,E2F1,E2F3,FHIT,ITGA3,ITGAV,LAMA1,LAMA4,LAMA5,LAMB2,LAMC1,LAMC2,LAMC3,MAX,NFKBIA,PIAS2,PIK3CA,PIK3CB,PIK3CG,PIK3R1,PTEN,PTGS2,RB1,RXRA,RXRG,TRAF1,TRAF2,TRAF3,TRAF4,TRAF6,XIAP
	Vibrio cholerae infection	predicted (union)	0.038	ACTB, ADCY3,ADCY9,ARF1,ATP6V0A2,ATP6V0B,ATP6V0D1,ATP6V0D2,ATP6V0E1,ATP6V0E2,ATP6V1A,ATP6V1E1,ATP6V1G1,ATP6V1G3,ERO1A,GNAS,KCNQ1,KDELR1,KDELR3,PDIA4,PLCG1,PRKACB,PRKCA,PRKX,SEC61A1,SEC61G,SLC12A2,TCIRG1,TJP1,TJP2
	Adherens junction	predicted (union)	0.039	ACTB,ACTN1,ACTN4,AFDN,BAIAP2,CDC42,CSNK2A1,CTNNA2,EGFR,EP300,ERBB2,FER,FGFR1,INSR,LEF1,LMO7,MAP3K7,MAPK1,MAPK3,MET,NECTIN3,NECTIN4,NLK,PTPRB,PTPRJ,RAC1,SMAD2,SMAD3,SNAI1,SORBS1,SRC,SSX2IP,TCF7L1,TCF7L2,TGFBR1,TGFBR2,TJP1,VCL,WASF1,WASL
	Chronic myeloid leukemia	predicted (union)	0.039	AKT2,ARAF,BAD,BCL2L1,BCR,CBL,CBLB,CCND1,CDK6,CDKN1B,CHUK,CRK,CTBP1,CTBP2,E2F1,E2F3,GAB2,HDAC2,KRAS,MAPK1,MAPK3,NFKBIA,NRAS,PIK3CA,PIK3CB,PIK3CG,PIK3R1,PTPN11,RB1,RUNX1,SHC3,SHC4,SMAD3,STAT5B,TGFB2,TGFB3,TGFBR1,TGFBR2
	Pancreatic cancer	predicted (union)	0.039	AKT2,ARAF,BAD,BCL2L1,CASP9,CCND1,CDC42,CDK6,CHUK,E2F1,E2F3,EGF,EGFR,ERBB2,KRAS,MAPK1,MAPK10,MAPK3,MAPK8,MAPK9,PIK3CA,PIK3CB,PIK3CG,PIK3R1,RAC1,RAD51,RALA,RB1,SMAD2,SMAD3,TGFA,TGFB2,TGFB3,TGFBR1,TGFBR2,VEGFA
	Renal cell carcinoma	predicted (union)	0.039	AKT2,ARAF,ARNT,ARNT2,CDC42,CRK,EGLN1,EGLN3,EP300,EPAS1,HGF,KRAS,MAPK1,MAPK3,MET,NRAS,PAK1,PAK2,PAK3,PAK4,PDGFB,PIK3CA,PIK3CB,PIK3CG,PIK3R1,PTPN11,RAC1,RAP1A,RAPGEF1,RBX1,TGFA,TGFB2,TGFB3,VEGFA,VHL
	Shigellosis	predicted (union)	0.039	ACTB,ARPC2,ARPC4,BTRC,CDC42,CHUK,CRK,CTTN,DIAPH1,DOCK1,FBXW11,HCLS1,MAPK1,MAPK10,MAPK11,MAPK13,MAPK14,MAPK3,MAPK8,MAPK9,NFKBIA,NFKBIB,NOD2,PFN1,PFN2,PFN4,RAC1,RIPK2,ROCK2,SRC,UBE2D2,VCL,WASF1,WASL
	Huntington's disease	predicted (union)	0.047	AP2A2,AP2M1,AP2S1,APAF1,ATP5F1A,ATP5F1B,ATP5MC1,ATP5MC3,ATP5PB,BAX,BBC3,CASP3,CASP9,CLTC,COX4I1,COX5A,COX6A2,COX6B1,COX7A2L,CREB1,CREB3L2,CREB5,CYCS,DCTN2,DNAH2,DNAL1,DNAL4,EP300,GNAQ,GRIN1,GRIN2B,HAP1,HDAC2,HIP1,IFT57,NDUFA10,NDUFA12,NDUFA3,NDUFA7,NDUFA9,NDUFB10,NDUFB4,NDUFB5,NDUFB6,NDUFC1,NDUFS1,NDUFS2,NDUFS6,NDUFV3,NRF1,PLCB1,PLCB2,PLCB3,PLCB4,POLR2B,POLR2D,POLR2E,POLR2G,PPARGC1A,PPIF,PTGS1,RCOR1,REST,SDHA,SDHC,SIN3A,SLC25A31,SLC25A4,SOD1,SOD2,SP1,TAF4,TAF4B,TBPL1,TFAM,UCP1,UQCR11,UQCRB,UQCRFS1,UQCRQ,VDAC1,VDAC2,VDAC3
	Thyroid hormone signaling pathway	predicted (union)	0.047	ACTB,AKT2,ATP1B3,ATP1B4,BAD,CASP9,CCND1,DIO2,EP300,ESR1,FOXO1,GATA4,GSK3B,HDAC2,ITGAV,ITGB3,KAT2A,KAT2B,KRAS,MAPK1,MAPK3,MED1,MED12L,MED13L,MED17,MED4,NCOA1,NCOA2,NCOR1,NOTCH2,NRAS,PDPK1,PFKFB2,PIK3CA,PIK3CB,PIK3CG,PIK3R1,PLCB1,PLCB2,PLCB3,PLCB4,PLCD4,PLCE1,PLCG1,PRKACB,PRKCA,PRKX,RCAN2,RXRA,RXRG,SIN3A,SLC16A2,SLC9A1,SLCO1C1,SRC,TBC1D4,THRA,THRB
	VEGF signaling pathway	predicted (union)	0.047	AKT2,BAD,CASP9,CDC42,KDR,KRAS,MAPK1,MAPK11,MAPK13,MAPK14,MAPK3,MAPKAPK2,MAPKAPK3,NFATC2,NOS3,NRAS,PIK3CA,PIK3CB,PIK3CG,PIK3R1,PLA2G4A,PLA2G4D,PLA2G4E,PLCG1,PPP3CC,PPP3R1,PPP3R2,PRKCA,PTGS2,PXN,RAC1,SRC,VEGFA
**hsa-miR-222-3p**	MicroRNAs in cancer	predicted (union)	9.58e-4	ABL1,ATM,BCL2L11,BCL2L2,BMF,BMI1,BMPR2,BRCA1,CASP3,CCND1,CCND2,CD44,CDK6,CDKN1B,COMMD3,BMI1,CRKL,CYP24A1,DDIT4,DICER1,DNMT3A,DNMT3B,E2F2,E2F3,EGFR,ERBB2,ERBB3,FOXP1,FZD3,GLS,HDAC4,HOXD10,IGF2BP1,IRS1,ITGB3,MAPK1,MCL1,MDM4,MMP16,NOTCH2,NOTCH3,PDGFA,PDGFRB,PIK3CA,PLCG1,PRKCB,PTEN,RASSF1,RDX,RECK,RHOA,RPTOR,SERPINB5,SIRT1,SLC45A3,SOCS1,SOS2,SOX4,STAT3,STMN1,TGFB2,THBS1,TIMP3,TNN,TNR,TP63,TRIM71,UBE2I,ZFPM2
	Retrograde endocannabinoid signaling	predicted (union)	9.58e-4	ADCY1,ADCY2,ADCY9,CACNA1C,CNR1,FAAH,GABRA1,GABRA3,GABRB1,GABRB3,GABRG1,GABRG3,GABRR1,GABRR2,GNAI2,GNAI3,GNAO1,GNAQ,GNB1,GNB3,GNB4,GNB5,GNG11,GNG12,GNG2,GNG7,GRIA2,GRIA4,GRM1,ITPR1,ITPR2,KCNJ5,KCNJ6,MAPK1,MAPK10,MAPK13,MAPK14,MAPK8,MAPK9,NAPEPLD,PLCB1,PLCB2,PRKACB,PRKCB,PRKX,SLC17A6,SLC17A8
	Wnt signaling pathway	predicted (union)	9.58e-4	AXIN2,BAMBI,BTRC,CCND1,CCND2,CHD8,CSNK1A1,CSNK1A1L,CSNK2A1,CTBP2,CTNNB1,CTNNBIP1,CXXC4,DAAM1,DAAM2,DKK2,DVL2,FRAT1,FRAT2,FZD1,FZD3,FZD4,FZD5,FZD8,GSK3B,LRP6,MAP3K7,MAPK10,MAPK8,MAPK9,NFATC2,NFATC3,NFATC4,NKD1,NLK,PLCB1,PLCB2,PPP3CB,PPP3CC,PPP3R1,PPP3R2,PRICKLE2,PRKACB,PRKCB,PRKX,PSEN1,RBX1,RHOA,SENP2,SFRP1,SFRP2,SKP1,TBL1X,TBL1XR1,TCF7L2,VANGL1,VANGL2,WIF1,WNT1,WNT10B,WNT2,WNT2B,WNT7A,WNT8B,WNT9B
	Proteoglycans in cancer	predicted (union)	0.008	AKT2,ANK2,ARHGEF12,CASP3,CAV2,CBL,CBLB,CCND1,CD44,COL21A1,CTNNB1,CTTN,EGFR,ELK1,ERBB2,ERBB3,ERBB4,ESR1,FASLG,FGF10,FGF12,FGF14,FGF19,FGF2,FGF5,FGF9,FN1,FRS2,FZD1,FZD3,FZD4,FZD5,FZD8,GAB1,HBEGF,HIF1A,HOXD10,HPSE,IGF1,IQGAP1,ITGA2,ITGB3,ITPR1,ITPR2,KDR,MAPK1,MAPK13,MAPK14,MRAS,MSN,PAK1,PDPK1,PIK3CA,PIK3CG,PIK3R1,PLAUR,PLCE1,PLCG1,PPP1R12A,PPP1R12B,PRKACB,PRKCB,PRKX,PTK2,PTPN11,PXN,RDX,RHOA,RPS6KB1,SDC1,SDC2,SMAD2,SOS2,STAT3,TGFB2,THBS1,TIAM1,TIMP3,TLR2,TNF,VAV2,VTN,WNT1,WNT10B,WNT2,WNT2B,WNT7A,WNT8B,WNT9B
	MicroRNAs in cancer	experimental (any)	0.009	BCL2L11, BMF,CDK6,CDKN1B,DICER1,EZR,MDM2,MYC,PTEN,RECK,STMN1,TIMP3,TP53,ZEB2
	ErbB signaling pathway	predicted (union)	0.014	ABL1,AKT2,CBL,CBLB,CDKN1B,CRKL,EGF,EGFR,ELK1,ERBB2,ERBB3,ERBB4,GAB1,GSK3B,HBEGF,MAP2K4,MAPK1,MAPK10,MAPK8,MAPK9,NCK1,NCK2,NRG1,NRG3,NRG4,PAK1,PAK2,PAK3,PIK3CA,PIK3CG,PIK3R1,PLCG1,PRKCB,PTK2,RPS6KB1,SHC3,SOS2,STAT5A,STAT5B,TGFA
	Morphine addiction	predicted (union)	0.014	ADCY1,ADCY2,ADCY9,DRD1,GABBR2,GABRA1,GABRA3,GABRB1,GABRB3,GABRG1,GABRG3,GABRR1,GABRR2,GNAI2,GNAI3,GNAO1,GNB1,GNB3,GNB4,GNB5,GNG11,GNG12,GNG2,GNG7,GRK3,GRK5,KCNJ5,KCNJ6,OPRM1,PDE10A,PDE11A,PDE1C,PDE3A,PDE4A,PDE4B,PDE4D,PDE7A,PDE7B,PRKACB,PRKCB,PRKX
	Glutamatergic synapse	predicted (union)	0.016	ADCY1,ADCY2,ADCY9,CACNA1C,DLGAP1,GLS,GLUL,GNAI2,GNAI3,GNAO1,GNAQ,GNB1,GNB3,GNB4,GNB5,GNG11,GNG12,GNG2,GNG7,GRIA2,GRIA4,GRIK3,GRIN2A,GRIN2B,GRIN3A,GRK3,GRM1,GRM2,GRM4,HOMER1,ITPR1,ITPR2,MAPK1,PLA2G4D,PLCB1,PLCB2,PPP3CB,PPP3CC,PPP3R1,PPP3R2,PRKACB,PRKCB,PRKX,SLC17A6,SLC17A8,SLC1A1,SLC1A2,SLC38A1,TRPC1
	Axon guidance	predicted (union)	0.029	ABL1, ABLIM1,ARHGEF12,CFL2,CXCL12,DPYSL2,DPYSL5,EFNA1,EFNA2,EFNB2,EPHA4,EPHA5,EPHA7,EPHA8,GNAI2,GNAI3,GSK3B,MAPK1,NCK1,NCK2,NFATC2,NFATC3,NFATC4,NRP1,PAK1,PAK2,PAK3,PLXNA2,PLXNA3,PLXNB2,PLXNC1,PPP3CB,PPP3CC,PPP3R1,PPP3R2,PTK2,RHOA,ROBO2,SEMA3A,SEMA3C,SEMA4B,SEMA4C,SEMA4F,SEMA4G,SEMA5A,SEMA6A,SEMA6D,SLIT1,SLIT2,SRGAP1,UNC5B,UNC5C,UNC5D

	Cell cycle	experimental (any)	0.045	CDC27, CDK6,CDKN1B,CDKN1C,MCM3,MCM7,MDM2,MYC,TP53,YWHAG
	DNA replication	experimental (any)	0.045	MCM3, MCM7,POLD2,POLE,RFC1
	Endometrial cancer	experimental (any)	0.045	AKT3, FOXO3,ILK,MYC,PTEN,TP53
	FoxO signaling pathway	experimental (any)	0.045	AKT3, BCL2L11,CDKN1B,FOXO1,FOXO3,IRS4,MDM2,PTEN,SOD2,TNFSF10
	Tight junction	experimental (any)	0.045	ACTB, ACTG1,AKT3,CLDN23,EPB41L2,GNAI2,GNAI3,PPP2R1A,PPP2R2A,PTEN
**hsa-miR-130a-3p**	TGF-beta signaling pathway	experimental (any)	1.17e-4	ACVR1, BMPR2,LEFTY1,MAPK1,MYC,SMAD4,SMAD5,TGFB1,TGFBR1,TGFBR2,TNF,ZFYVE9
	Cytokine-cytokine receptor interaction	experimental (strong)	0.014	CSF1, IL18,PDGFRA,TGFB1,TGFBR2,TNF
	HTLV-I infection	experimental (strong)	0.014	MYC, PDGFRA,SMAD4,TGFB1,TGFBR2,TNF,XIAP
	Rheumatoid arthritis	experimental (strong)	0.014	CSF1, IL18,TGFB1,TNF
	TGF-beta signaling pathway	experimental (strong)	0.014	MYC, SMAD4,TGFB1,TGFBR2,TNF
	Collecting duct acid secretion	experimental (any)	0.014	ATP6V0D1, ATP6V0E1,ATP6V1B2,ATP6V1C1,SLC12A7
	Adherens junction	experimental (any)	0.017	CSNK2A1, MAPK1,MET,SMAD4,TCF7L2,TGFBR1,TGFBR2,WASL
	Endocytosis	experimental (any)	0.017	DNM2,HSPA8,LDLR,MET,PDGFRA,RAB11FIP1,RAB5A,RAB5B,TGFB1,TGFBR1,TGFBR2,VPS37A,VPS37B,ZFYVE9
	FoxO signaling pathway	predicted (union)	0.021	AKT2,ATG12,ATM,BCL2L11,BNIP3,CCNB1,CCND1,CCND2,CCNG2,CDK2,CDKN1A,CDKN1B,CDKN2B,CHUK,CREBBP,EGFR,EP300,FASLG,FBXO32,FOXO3,G6PC,GABARAPL1,GADD45A,GADD45B,GADD45G,GRM1,HOMER1,IGF1,INSR,IRS1,KLF2,MAP2K1,MAP2K2,MAPK1,MAPK10,MAPK11,MAPK13,MAPK14,MAPK8,MAPK9,NLK,NRAS,PDPK1,PIK3CA,PIK3CB,PIK3R1,PIK3R2,PIK3R3,PRKAA1,PRKAA2,PRKAB1,PRKAG2,PRMT1,PTEN,RAF1,RAG1,S1PR1,SETD7,SIRT1,SMAD2,SOD2,SOS2,STAT3,STK11,STK4,TGFB1,TGFB2,TGFBR1,TGFBR2,TNFSF10,USP7
	Hippo signaling pathway	predicted (union)	0.021	AJUBA,AMOT,AXIN2,BBC3,BIRC5,BMP2,BMP6,BMP7,BMP8A,BMP8B,BMPR1A,BMPR1B,BMPR2,BTRC,CCN2,CCND1,CCND2,CCND3,CDH1,CRB1,CSNK1D,CTNNA3,DLG1,DLG2,DLG3,FBXW11,FGF1,FRMD1,FRMD6,FZD3,FZD4,FZD5,FZD6,FZD8,GDF5,GDF6,GDF7,GSK3B,LATS1,LEF1,LIMD1,LLGL2,MOB1A,MOB1B,MPP5,NF2,NKD1,PARD6B,PARD6G,PATJ,PPP1CB,PPP2CA,PPP2R1A,PPP2R1B,PPP2R2A,PPP2R2C,RASSF6,SAV1,SERPINE1,SMAD2,SMAD7,STK3,TCF7,TCF7L1,TCF7L2,TGFB1,TGFB2,TGFBR1,TGFBR2,TP73,WNT1,WNT10A,WNT10B,WNT2B,WNT3A,WNT5B,WNT7B,WNT8B,WNT9A,WNT9B,WTIP,WWC1,YWHAB,YWHAG,YWHAH
	Pancreatic cancer	predicted (union)	0.021	AKT2,ARHGEF6,CASP9,CCND1,CDC42,CDK4,CDK6,CHUK,E2F1,E2F2,E2F3,EGFR,JAK1,MAP2K1,MAPK1,MAPK10,MAPK8,MAPK9,PIK3CA,PIK3CB,PIK3R1,PIK3R2,PIK3R3,PLD1,RAC1,RAC2,RAD51,RAF1,RALA,RALBP1,RB1,SMAD2,STAT1,STAT3,TGFA,TGFB1,TGFB2,TGFBR1,TGFBR2,TP53
	Pathways in cancer	predicted (union)	0.021	ABL1,AKT2,APPL1,AR,ARNT,AXIN2,BCL2,BID,BIRC3,BIRC5,BMP2,CASP9,CBLB,CCDC6,CCND1,CDC42,CDH1,CDK2,CDK4,CDK6,CDKN1A,CDKN1B,CDKN2B,CHUK,CKS1B,CKS2,COL4A1,COL4A2,COL4A3,COL4A4,CREBBP,CRKL,CSF2RA,CTBP2,CTNNA3,CYCS,DCC,E2F1,E2F2,E2F3,EGFR,EGLN1,EGLN3,ELOC,EP300,EPAS1,FASLG,FGF1,FGF10,FGF11,FGF12,FGF14,FGF19,FGF2,FGF4,FGF5,FGF9,FGFR1,FN1,FOS,FZD3,FZD4,FZD5,FZD6,FZD8,GLI3,GSK3B,HDAC2,HHIP,HIF1A,HSP90AA1,HSP90B1,IGF1,ITGA2,ITGA3,JAK1,KIT,LAMA4,LAMB2,LAMC1,LAMC2,LAMC3,LEF1,MAP2K1,MAP2K2,MAPK1,MAPK10,MAPK8,MAPK9,MET,MITF,MMP2,MSH2,MSH3,NFKBIA,NKX3-1,NRAS,PAX8,PDGFB,PDGFRA,PIAS2,PIK3CA,PIK3CB,PIK3R1,PIK3R2,PIK3R3,PLCG1,PLD1,PML,PPARD,PPARG,PTCH2,PTEN,PTGS2,RAC1,RAC2,RAD51,RAF1,RALA,RALBP1,RARB,RB1,RET,RHOA,RUNX1,RUNX1T1,RXRA,RXRG,SHH,SLC2A1,SMAD2,SOS2,STAT1,STAT3,STK4,SUFU,TCF7,TCF7L1,TCF7L2,TGFA,TGFB1,TGFB2,TGFBR1,TGFBR2,TP53,TPM3,TRAF1,TRAF2,TRAF3,TRAF6,VHL,WNT1,WNT10A,WNT10B,WNT2B,WNT3A,WNT5B,WNT7B,WNT8B,WNT9A,WNT9B,XIAP,ZBTB16
	Prostate cancer	predicted (union)	0.021	AKT2,AR,BCL2,CASP9,CCND1,CDK2,CDKN1A,CDKN1B,CHUK,CREB1,CREB3L2,CREB3L3,CREB5,CREBBP,E2F1,E2F2,E2F3,EGFR,EP300,FGFR1,GSK3B,HSP90AA1,HSP90B1,IGF1,LEF1,MAP2K1,MAP2K2,MAPK1,NFKBIA,NKX3-1,NRAS,PDGFB,PDGFC,PDGFD,PDGFRA,PDPK1,PIK3CA,PIK3CB,PIK3R1,PIK3R2,PIK3R3,PTEN,RAF1,RB1,SOS2,TCF7,TCF7L1,TCF7L2,TGFA,TP53
	Small cell lung cancer	predicted (union)	0.021	AKT2,APAF1,BCL2,BIRC3,CASP9,CCND1,CDK2,CDK4,CDK6,CDKN1B,CDKN2B,CHUK,CKS1B,CKS2,COL4A1,COL4A2,COL4A3,COL4A4,CYCS,E2F1,E2F2,E2F3,FHIT,FN1,ITGA2,ITGA3,LAMA4,LAMB2,LAMC1,LAMC2,LAMC3,NFKBIA,PIAS2,PIK3CA,PIK3CB,PIK3R1,PIK3R2,PIK3R3,PTEN,PTGS2,RARB,RB1,RXRA,RXRG,TP53,TRAF1,TRAF2,TRAF3,TRAF6,XIAP
	TGF-beta signaling pathway	predicted (union)	0.021	ACVR1,ACVR1B,ACVR1C,ACVR2A,ACVR2B,BAMBI,BMP2,BMP6,BMP7,BMP8A,BMP8B,BMPR1A,BMPR1B,BMPR2,CDKN2B,CREBBP,E2F5,EP300,GDF5,GDF6,GDF7,ID4,INHBA,INHBB,INHBC,LEFTY1,LTBP1,MAPK1,NODAL,NOG,PPP2CA,PPP2R1A,PPP2R1B,RBL1,RHOA,ROCK1,RPS6KB1,SKP1,SMAD2,SMAD5,SMAD7,SMURF1,SMURF2,SP1,TGFB1,TGFB2,TGFBR1,TGFBR2,TNF,ZFYVE16
	Colorectal cancer	experimental (strong)	0.021	MYC, SMAD4,TGFB1,TGFBR2
	Osteoclast differentiation	experimental (strong)	0.021	CSF1, PPARG,TGFB1,TGFBR2,TNF
	Rheumatoid arthritis	experimental (any)	0.024	ATP6V0D1, ATP6V0E1,ATP6V1B2,ATP6V1C1,CSF1,IL18,TGFB1,TNF

Adherens junction	predicted (union)	0.025	ACP1,ACTN2,BAIAP2,CDC42,CDH1,CREBBP,CSNK2A1,CTNNA3,EGFR,EP300,FARP2,FER,FGFR1,FYN,INSR,IQGAP1,LEF1,LMO7,MAPK1,MET,NECTIN1,NECTIN2,NECTIN3,NLK,PTPN1,PTPRB,PTPRJ,PTPRM,RAC1,RAC2,RHOA,SMAD2,SRC,SSX2IP,TCF7,TCF7L1,TCF7L2,TGFBR1,TGFBR2,VCL,WASF3,WASL,YES1
Axon guidance	predicted (union)	0.025	ABL1,ABLIM1,ARHGEF12,CDC42,CFL2,CXCL12,DCC,DPYSL2,DPYSL5,EFNA2,EFNA4,EFNA5,EFNB1,EFNB2,EPHA3,EPHA4,EPHA5,EPHA6,EPHA7,EPHA8,FYN,GSK3B,LRRC4,LRRC4C,MAPK1,MET,NCK1,NFATC2,NFATC4,NRAS,NRP1,NTN1,NTN4,NTNG1,NTNG2,PAK3,PAK4,PAK6,PLXNA1,PLXNA2,PLXNA3,PLXNB1,PLXNC1,PPP3CB,RAC1,RAC2,RASA1,RHOA,ROBO1,ROBO2,ROCK1,ROCK2,SEMA3A,SEMA3C,SEMA3G,SEMA4B,SEMA4F,SEMA4G,SEMA5A,SEMA5B,SEMA6D,SEMA7A,SLIT1,SLIT2,SLIT3,SRGAP1,SRGAP3,UNC5C,UNC5D
Colorectal cancer	predicted (union)	0.025	AKT2,APPL1,AXIN2,BCL2,BIRC5,CASP9,CCND1,CYCS,DCC,FOS,GSK3B,LEF1,MAP2K1,MAPK1,MAPK10,MAPK8,MAPK9,MSH2,MSH3,PIK3CA,PIK3CB,PIK3R1,PIK3R2,PIK3R3,RAC1,RAC2,RAF1,RHOA,SMAD2,TCF7,TCF7L1,TCF7L2,TGFB1,TGFB2,TGFBR1,TGFBR2,TP53
Glioma	predicted (union)	0.025	AKT2,CALM1,CALM2,CALML3,CAMK2A,CAMK2B,CAMK2D,CAMK2G,CCND1,CDK4,CDK6,CDKN1A,E2F1,E2F2,E2F3,EGFR,IGF1,MAP2K1,MAP2K2,MAPK1,NRAS,PDGFB,PDGFRA,PIK3CA,PIK3CB,PIK3R1,PIK3R2,PIK3R3,PLCG1,PTEN,RAF1,RB1,SHC3,SHC4,SOS2,TGFA,TP53
Neurotrophin signaling pathway	predicted (union)	0.025	ABL1,AKT2,BCL2,BDNF,BEX3,CALM1,CALM2,CALML3,CAMK2A,CAMK2B,CAMK2D,CAMK2G,CAMK4,CDC42,CRKL,FASLG,FOXO3,FRS2,GAB1,GSK3B,IRAK1,IRAK2,IRAK3,IRAK4,IRS1,KIDINS220,MAP2K1,MAP2K2,MAP3K1,MAP3K3,MAP3K5,MAPK1,MAPK10,MAPK11,MAPK13,MAPK14,MAPK8,MAPK9,NFKBIA,NRAS,NTRK2,NTRK3,PDPK1,PIK3CA,PIK3CB,PIK3R1,PIK3R2,PIK3R3,PLCG1,PSEN1,PTPN11,RAC1,RAF1,RAP1A,RHOA,RPS6KA2,RPS6KA3,RPS6KA6,SH2B3,SHC3,SHC4,SORT1,SOS2,TP53,TP73,TRAF6,ZNF274
Prolactin signaling pathway	predicted (union)	0.025	AKT2,CCND1,CCND2,ELF5,ESR1,ESR2,FOS,FOXO3,GSK3B,IRF1,JAK2,LHCGR,MAP2K1,MAP2K2,MAPK1,MAPK10,MAPK11,MAPK13,MAPK14,MAPK8,MAPK9,NRAS,PIK3CA,PIK3CB,PIK3R1,PIK3R2,PIK3R3,RAF1,SHC3,SHC4,SLC2A2,SOCS2,SOCS4,SOCS5,SOCS6,SOS2,SRC,STAT1,STAT3,TH,TNFRSF11A
mTOR signaling pathway	predicted (union)	0.025	AKT2,CAB39,CAB39L,EIF4B,EIF4E,EIF4E2,EIF4EBP1,HIF1A,IGF1,IRS1,MAPK1,MLST8,PDPK1,PIK3CA,PIK3CB,PIK3R1,PIK3R2,PIK3R3,PRKAA1,PRKAA2,PTEN,RICTOR,RPS6,RPS6KA2,RPS6KA3,RPS6KA6,RPS6KB1,RRAGC,RRAGD,STK11,STRADA,TNF,TSC1,TSC2,ULK2,ULK3
Malaria	experimental (strong)	0.025	IL18, TGFB1,TNF
Pathways in cancer	experimental (strong)	0.025	MYC, PDGFRA,PPARG,PTEN,SMAD4,TGFB1,TGFBR2,XIAP
Colorectal cancer	experimental (any)	0.025	MAPK1, MYC,SMAD4,TCF7L2,TGFB1,TGFBR1,TGFBR2
Cytokine-cytokine receptor interaction	experimental (any)	0.025	ACVR1,BMPR2,CCR6,CSF1,IFNLR1,IL18,IL23R,MET,PDGFRA,TGFB1,TGFBR1,TGFBR2,TNF,TNFRSF10B
MAPK signaling pathway	experimental (strong)	0.026	MAP3K12, MYC,PDGFRA,TGFB1,TGFBR2,TNF
FoxO signaling pathway	experimental (any)	0.026	CCND2, G6PC,MAPK1,PRKAA1,PTEN,RAG1,SMAD4,TGFB1,TGFBR1,TGFBR2
Glycosaminoglycan biosynthesis - chondroitin sulfate / dermatan sulfate	predicted (union)	0.028	B3GAT3,B4GALT7,CHST11,CHST12,CHST13,CHST14,CHST15,CHST3,CHSY1,CHSY3,CSGALNACT1,CSGALNACT2,DSE,UST,XYLT1,XYLT2
Chronic myeloid leukemia	experimental (strong)	0.029	MYC, SMAD4,TGFB1,TGFBR2
Hepatitis B	experimental (strong)	0.033	MYC, PTEN,SMAD4,TGFB1,TNF
Estrogen signaling pathway	predicted (union)	0.034	ADCY1,ADCY2,ADCY4,ADCY5,ADCY9,AKT2,ATF2,CALM1,CALM2,CALML3,CREB1,CREB3L2,CREB3L3,CREB5,EGFR,ESR1,ESR2,FKBP4,FKBP5,FOS,GABBR1,GABBR2,GNAO1,GNAQ,GPER1,GRM1,HSP90AA1,HSP90B1,ITPR1,ITPR2,KCNJ3,KCNJ5,KCNJ6,MAP2K1,MAP2K2,MAPK1,MMP2,NRAS,OPRM1,PIK3CA,PIK3CB,PIK3R1,PIK3R2,PIK3R3,PLCB1,PLCB4,PRKACB,PRKX,RAF1,SHC3,SHC4,SOS2,SP1,SRC
MicroRNAs in cancer	predicted (union)	0.034	ABCC1,ABL1,ATM,BAK1,BCL2,BCL2L11,BCL2L2,BMF,BMPR2,CCND1,CCND2,CD44,CDK6,CDKN1A,CDKN1B,CREBBP,CRKL,DICER1,DNMT1,DNMT3A,E2F1,E2F2,E2F3,EGFR,EP300,ERBB3,EZR,FOXP1,FSCN1,FZD3,GLS,HDAC4,HMGA2,HNRNPK,IGF2BP1,IRS1,ITGA5,ITGB3,KIF23,MAP2K1,MAP2K2,MAPK1,MCL1,MDM4,MET,MMP16,NOTCH1,NOTCH2,NRAS,PAK4,PDGFB,PDGFRA,PIK3CA,PIK3R2,PLCG1,PRKCE,PTEN,PTGS2,RAF1,RDX,RHOA,ROCK1,SHC4,SIRT1,SLC45A3,SLC7A1,SOS2,SOX4,STAT3,TGFB2,TNN,TNR,TP53,TP63,TRIM71,UBE2I,WNT3A,ZEB1,ZFPM2
Proteoglycans in cancer	predicted (union)	0.034	AKT2,ANK2,ARHGEF12,CAMK2A,CAMK2B,CAMK2D,CAMK2G,CAV2,CBLB,CCND1,CD44,CDC42,CDKN1A,CTSL,DDX5,EGFR,EIF4B,ELK1,ERBB3,ERBB4,ESR1,EZR,FASLG,FGF1,FGF10,FGF11,FGF12,FGF14,FGF19,FGF2,FGF4,FGF5,FGF9,FGFR1,FLNC,FN1,FRS2,FZD3,FZD4,FZD5,FZD6,FZD8,GAB1,HIF1A,HPSE,HPSE2,IGF1,IQGAP1,ITGA2,ITGA5,ITGB3,ITPR1,ITPR2,KDR,LUM,MAP2K1,MAP2K2,MAPK1,MAPK11,MAPK13,MAPK14,MET,MMP2,MRAS,NRAS,NUDT16L1,PDPK1,PIK3CA,PIK3CB,PIK3R1,PIK3R2,PIK3R3,PLCE1,PLCG1,PPP1CB,PPP1R12A,PPP1R12B,PRKACB,PRKX,PTPN11,RAC1,RAF1,RDX,RHOA,ROCK1,ROCK2,RPS6,RPS6KB1,SDC1,SDC2,SDC4,SMAD2,SOS2,SRC,STAT3,TGFB1,TGFB2,TNF,TP53,TWIST1,VAV2,WNT1,WNT10A,WNT10B,WNT2B,WNT3A,WNT5B,WNT7B,WNT8B,WNT9A,WNT9B
SNARE interactions in vesicular transport	predicted (union)	0.034	BET1L,BNIP1,GOSR1,SNAP23,SNAP25,SNAP29,SNAP47,STX10,STX11,STX16,STX18,STX1A,STX2,STX3,STX4,STX6,STX7,VAMP1,VAMP3,VAMP4,VAMP5,VTI1A,VTI1B,YKT6

	Bladder cancer	experimental (any)	0.035	CDK4, DAPK1,MAPK1,MYC,RPS6KA5
	Chronic myeloid leukemia	experimental (any)	0.035	CDK4, MAPK1,MYC,SMAD4,TGFB1,TGFBR1,TGFBR2
	Hepatitis B	experimental (any)	0.035	CCNA2, CDK4,MAPK1,MAVS,MYC,PTEN,SMAD4,TGFB1,TGFBR1,TNF
	Hippo signaling pathway	experimental (any)	0.035	BMPR2, CCND2,FZD6,MYC,NF2,SMAD4,TCF7L2,TGFB1,TGFBR1,TGFBR2,WNT10A
	MicroRNAs in cancer	experimental (any)	0.035	BMPR2, CCND2,DICER1,MAPK1,MET,MYC,PDGFRA,PTEN,RPS6KA5,SOX4,TRIM71
	Melanoma	predicted (union)	0.036	AKT2,CCND1,CDH1,CDK4,CDK6,CDKN1A,E2F1,E2F2,E2F3,EGFR,FGF1,FGF10,FGF11,FGF12,FGF14,FGF19,FGF2,FGF4,FGF5,FGF9,FGFR1,IGF1,MAP2K1,MAP2K2,MAPK1,MET,MITF,NRAS,PDGFB,PDGFC,PDGFD,PDGFRA,PIK3CA,PIK3CB,PIK3R1,PIK3R2,PIK3R3,PTEN,RAF1,RB1,TP53
	Endocytosis	predicted (union)	0.039	AGAP1,AGAP3,ARAP2,ARFGAP1,ARRB1,ASAP1,ASAP2,CAV2,CBLB,CDC42,CHMP1B,CHMP3,CHMP4B,CHMP4C,CLTC,CXCR2,DNAJC6,DNM3,EEA1,EGFR,EHD2,EHD3,EHD4,EPN1,EPN2,EPS15,ERBB3,ERBB4,FLT1,GIT2,GRK1,GRK2,GRK3,GRK5,GRK7,HLA-B,HLA-C,HLA-E,IL2RA,IL2RB,IQSEC2,IQSEC3,ITCH,KDR,KIT,LDLR,MET,MVB12B,PARD6B,PARD6G,PDCD6IP,PDGFRA,PIP5K1C,PLD1,PML,PSD,PSD3,PSD4,RAB11A,RAB11FIP1,RAB11FIP2,RAB11FIP5,RAB22A,RAB31,RAB4A,RAB5A,RAB5B,RBSN,RET,RHOA,RNF103-CHMP3,RNF41,SH3GLB1,SH3GLB2,SH3KBP1,SMAD2,SMAD7,SMAP1,SMURF1,SMURF2,SNF8,SRC,STAM2,STAMBP,TGFB1,TGFB2,TGFBR1,TGFBR2,TRAF6,TSG101,USP8,VPS37A,VPS37B,VPS37C,VPS4A,VPS4B,VTA1,WWP1,ZFYVE16
	Endocytosis	experimental (strong)	0.040	PDGFRA, RAB5A,TGFB1,TGFBR2
	Inflammatory bowel disease (IBD)	experimental (strong)	0.040	IL18, TGFB1,TNF
	Adipocytokine signaling pathway	experimental (strong)	0.041	PPARA, PPARGC1A,TNF
	Tuberculosis	experimental (strong)	0.041	IL18, RAB5A,TGFB1,TNF
	Pathways in cancer	experimental (any)	0.045	CDK4,DAPK1,EGLN3,FZD6,MAPK1,MET,MYC,PDGFRA,PPARG,PTEN,SMAD4,TCF7L2,TGFB1,TGFBR1,TGFBR2,WNT10A,XIAP
	HTLV-I infection	experimental (any)	0.049	CCND2, CDK4,CHEK2,FZD6,MYC,PDGFRA,RAN,SMAD4,TGFB1,TGFBR1,TGFBR2,TNF,WNT10A,XIAP
	Thyroid cancer	experimental (any)	0.049	MAPK1, MYC,PPARG,TCF7L2
**hsa-miR-143-3p**	Glioma	experimental (any)	2.52e-8	AKT1, AKT2,BRAF,CALM3,HRAS,IGF1R,KRAS,MAPK1,MDM2,PDGFB,PDGFRA,PIK3R1
	Focal adhesion	experimental (any)	1.09e-7	AKT1,AKT2,BCL2,BRAF,COL1A1,COL3A1,COL5A1,COL5A2,HRAS,IGF1R,ITGB1,ITGB4,MAPK1,PDGFB,PDGFRA,PIK3R1,XIAP
	Melanoma	experimental (any)	1.09e-7	AKT1, AKT2,BRAF,HRAS,IGF1R,KRAS,MAPK1,MDM2,PDGFB,PDGFRA,PIK3R1
	MicroRNAs in cancer	experimental (any)	1.09e-7	BCL2,CD44,CDC25B,DNMT3A,ERBB3,FSCN1,HRAS,KRAS,MAPK1,MAPK7,MDM2,MMP9,PDGFB,PDGFRA,PTGS2,TRIM71
	PI3K-Akt signaling pathway	experimental (any)	1.09e-7	AKT1,AKT2,BCL2,COL1A1,COL3A1,COL5A1,COL5A2,HRAS,IGF1R,ITGB1,ITGB4,KRAS,MAPK1,MDM2,PDGFB,PDGFRA,PIK3R1,PPP2R2A,PPP2R5E,TLR2,YWHAB
	Prostate cancer	experimental (any)	1.09e-7	AKT1, AKT2,BCL2,BRAF,HRAS,IGF1R,KRAS,MAPK1,MDM2,PDGFB,PDGFRA,PIK3R1
	Proteoglycans in cancer	experimental (any)	4.86e-7	AKT1,AKT2,BRAF,CD44,ERBB3,HRAS,IGF1R,ITGB1,KRAS,MAPK1,MDM2,MMP2,MMP9,PIK3R1,SDC1,TLR2,TNF
	Hepatitis B	experimental (any)	1.51e-6	AKT1, AKT2,BCL2,HRAS,KRAS,MAPK1,MMP9,NFATC1,PIK3R1,SMAD3,TLR2,TNF,YWHAB
	Pathways in cancer	experimental (any)	4.74e-6	AKT1,AKT2,BCL2,BRAF,HRAS,IGF1R,ITGB1,KRAS,MAPK1,MDM2,MMP2,MMP9,NFKB2,PDGFB,PDGFRA,PIK3R1,PTGS2,SMAD3,XIAP
	Proteoglycans in cancer	experimental (strong)	6.03e-6	AKT1, AKT2,BRAF,CD44,HRAS,IGF1R,ITGB1,KRAS,MDM2,MMP2,MMP9,SDC1,TLR2,TNF
	Toxoplasmosis	experimental (any)	7.61e-6	AKT1, AKT2,BCL2,IL10RB,ITGB1,MAP3K7,MAPK1,PIK3R1,TLR2,TNF,XIAP
	Bladder cancer	experimental (any)	1.42e-5	BRAF, HRAS,KRAS,MAPK1,MDM2,MMP2,MMP9
	Chronic myeloid leukemia	experimental (any)	1.42e-5	AKT1, AKT2,BRAF,HRAS,KRAS,MAPK1,MDM2,PIK3R1,SMAD3
	Estrogen signaling pathway	experimental (any)	1.42e-5	AKT1, AKT2,CALM3,HRAS,KCNJ3,KRAS,MAPK1,MMP2,MMP9,PIK3R1
	Non-small cell lung cancer	experimental (any)	1.42e-5	AKT1, AKT2,BRAF,FHIT,HRAS,KRAS,MAPK1,PIK3R1
	FoxO signaling pathway	experimental (any)	2.10e-5	AKT1, AKT2,BRAF,GABARAPL1,HRAS,IGF1R,KRAS,MAPK1,MDM2,PIK3R1,SMAD3
	Colorectal cancer	experimental (any)	3.18e-5	AKT1, AKT2,BCL2,BRAF,KRAS,MAPK1,PIK3R1,SMAD3
	Hepatitis C	experimental (any)	4.45e-5	AKT1, AKT2,BRAF,HRAS,KRAS,MAPK1,OAS3,PIK3R1,PPP2R2A,TNF
	Rap1 signaling pathway	experimental (any)	4.45e-5	AKT1, AKT2,BRAF,CALM3,CTNND1,HRAS,IGF1R,ITGB1,KRAS,MAPK1,PDGFB,PDGFRA,PIK3R1

Renal cell carcinoma	experimental (any)	4.45e-5	AKT1, AKT2,BRAF,HRAS,KRAS,MAPK1,PDGFB,PIK3R1
Neurotrophin signaling pathway	experimental (any)	5.84e-5	AKT1, AKT2,BCL2,BRAF,CALM3,HRAS,KRAS,MAPK1,MAPK7,PIK3R1
Endometrial cancer	experimental (any)	6.41e-5	AKT1, AKT2,BRAF,HRAS,KRAS,MAPK1,PIK3R1
T cell receptor signaling pathway	experimental (any)	9.47e-5	AKT1, AKT2,HRAS,KRAS,MAP3K7,MAPK1,NFATC1,PIK3R1,TNF
Chagas disease (American trypanosomiasis)	experimental (any)	9.92e-5	AKT1, AKT2,MAPK1,PIK3R1,PPP2R2A,SERPINE1,SMAD3,TLR2,TNF
ECM-receptor interaction	experimental (any)	9.92e-5	CD44, COL1A1,COL3A1,COL5A1,COL5A2,ITGB1,ITGB4,SDC1
HTLV-I infection	experimental (any)	9.92e-5	AKT1, AKT2,HRAS,KRAS,NFATC1,NFKB2,NFYB,PDGFB,PDGFRA,PIK3R1,SMAD3,TERT,TNF,XIAP
MAPK signaling pathway	experimental (any)	9.92e-5	AKT1,AKT2,BRAF,CDC25B,HRAS,KRAS,MAP3K7,MAPK1,MAPK7,NFATC1,NFKB2,PDGFB,PDGFRA,TNF
Progesterone-mediated oocyte maturation	experimental (any)	9.92e-5	AKT1, AKT2,BRAF,CDC25B,IGF1R,KRAS,MAPK1,PIK3R1
Acute myeloid leukemia	experimental (any)	9.98e-5	AKT1, AKT2,BRAF,HRAS,KRAS,MAPK1,PIK3R1
VEGF signaling pathway	experimental (any)	9.98e-5	AKT1, AKT2,HRAS,KRAS,MAPK1,PIK3R1,PTGS2
TNF signaling pathway	experimental (any)	1.05e-4	AKT1, AKT2,MAP3K7,MAPK1,MMP14,MMP9,PIK3R1,PTGS2,TNF
Small cell lung cancer	experimental (any)	1.38e-4	AKT1, AKT2,BCL2,FHIT,ITGB1,PIK3R1,PTGS2,XIAP
Gap junction	experimental (any)	1.46e-4	GJD2, HRAS,KRAS,MAPK1,MAPK7,PDGFB,PDGFRA,TUBB2A
ErbB signaling pathway	experimental (any)	1.84e-4	AKT1, AKT2,BRAF,ERBB3,HRAS,KRAS,MAPK1,PIK3R1
Fc epsilon RI signaling pathway	experimental (any)	1.86e-4	AKT1, AKT2,HRAS,KRAS,MAPK1,PIK3R1,TNF
Pancreatic cancer	experimental (any)	2.21e-4	AKT1, AKT2,BRAF,KRAS,MAPK1,PIK3R1,SMAD3
B cell receptor signaling pathway	experimental (any)	2.62e-4	AKT1, AKT2,HRAS,KRAS,MAPK1,NFATC1,PIK3R1
Osteoclast differentiation	experimental (any)	3.11e-4	AKT1, AKT2,LILRB1,MAP3K7,MAPK1,NFATC1,NFKB2,PIK3R1,TNF
Epstein-Barr virus infection	experimental (any)	3.51e-4	AKT1, AKT2,BCL2,CD44,IL10RB,MAP3K7,MDM2,NFKB2,PIK3R1,POLR3A,YWHAB
Bladder cancer	experimental (strong)	3.65e-4	BRAF, HRAS,KRAS,MDM2,MMP2,MMP9
Glioma	experimental (strong)	3.65e-4	AKT1, AKT2,BRAF,HRAS,IGF1R,KRAS,MDM2
Melanoma	experimental (strong)	3.65e-4	AKT1, AKT2,BRAF,HRAS,IGF1R,KRAS,MDM2
Prostate cancer	experimental (strong)	3.65e-4	AKT1, AKT2,BCL2,BRAF,HRAS,IGF1R,KRAS,MDM2
Cholinergic synapse	experimental (any)	3.73e-4	AKT1, AKT2,BCL2,HRAS,KCNJ3,KRAS,MAPK1,PIK3R1
HIF-1 signaling pathway	experimental (any)	4.20e-4	AKT1, AKT2,BCL2,HK2,IGF1R,MAPK1,PIK3R1,SERPINE1
Regulation of actin cytoskeleton	experimental (any)	5.25e-4	BRAF, HRAS,ITGB1,ITGB4,KRAS,LIMK1,MAPK1,NCKAP1,PDGFB,PDGFRA,PIK3R1
Insulin signaling pathway	experimental (any)	5.73e-4	AKT1, AKT2,BRAF,CALM3,HK2,HRAS,KRAS,MAPK1,PIK3R1
Long-term depression	experimental (any)	6.23e-4	BRAF, HRAS,IGF1R,KRAS,MAPK1,NOS1
mTOR signaling pathway	experimental (any)	6.23e-4	AKT1, AKT2,BRAF,MAPK1,PIK3R1,TNF
Thyroid hormone signaling pathway	experimental (any)	6.89e-4	AKT1, AKT2,HRAS,KRAS,MAPK1,MDM2,PIK3R1,PLCD3
Hepatitis B	experimental (strong)	7.11e-4	AKT1, AKT2,BCL2,HRAS,KRAS,MMP9,NFATC1,TLR2,TNF
MicroRNAs in cancer	experimental (strong)	7.11e-4	BCL2, CD44,DNMT3A,FSCN1,HRAS,KRAS,MAPK7,MDM2,MMP9,PTGS2

Pathways in cancer	experimental (strong)	7.11e-4	AKT1, AKT2,BCL2,BRAF,HRAS,IGF1R,ITGB1,KRAS,MDM2,MMP2,MMP9,NFKB2,PTGS2
Non-small cell lung cancer	experimental (strong)	8.09e-4	AKT1, AKT2,BRAF,FHIT,HRAS,KRAS
Focal adhesion	experimental (strong)	8.32e-4	AKT1, AKT2,BCL2,BRAF,COL3A1,HRAS,IGF1R,ITGB1,ITGB4
GnRH signaling pathway	experimental (any)	8.58e-4	CALM3, HRAS,KRAS,MAPK1,MAPK7,MMP14,MMP2
Leishmaniasis	experimental (any)	9.36e-4	ITGB1, MAP3K7,MAPK1,PTGS2,TLR2,TNF
FoxO signaling pathway	experimental (strong)	9.45e-4	AKT1, AKT2,BRAF,GABARAPL1,HRAS,IGF1R,KRAS,MDM2
PI3K-Akt signaling pathway	experimental (strong)	9.45e-4	AKT1, AKT2,BCL2,COL3A1,HRAS,IGF1R,ITGB1,ITGB4,KRAS,MDM2,TLR2
Estrogen signaling pathway	experimental (strong)	0.001	AKT1, AKT2,HRAS,KRAS,MMP2,MMP9
Prolactin signaling pathway	experimental (any)	0.001	AKT1, AKT2,HRAS,KRAS,MAPK1,PIK3R1
Serotonergic synapse	experimental (any)	0.001	BRAF, CYP2C9,HRAS,KCNJ3,KRAS,MAPK1,PTGS2
Toll-like receptor signaling pathway	experimental (any)	0.001	AKT1, AKT2,MAP3K7,MAPK1,PIK3R1,TLR2,TNF
Tuberculosis	experimental (any)	0.001	AKT1, AKT2,BCL2,CALM3,IL10RB,MAPK1,NFYB,TLR2,TNF
Chronic myeloid leukemia	experimental (strong)	0.002	AKT1, AKT2,BRAF,HRAS,KRAS,MDM2
ECM-receptor interaction	experimental (strong)	0.002	CD44, COL3A1,ITGB1,ITGB4,SDC1
Endometrial cancer	experimental (strong)	0.002	AKT1, AKT2,BRAF,HRAS,KRAS
MAPK signaling pathway	experimental (strong)	0.002	AKT1, AKT2,BRAF,HRAS,KRAS,MAPK7,NFATC1,NFKB2,TNF
Neurotrophin signaling pathway	experimental (strong)	0.002	AKT1, AKT2,BCL2,BRAF,HRAS,KRAS,MAPK7
Serotonergic synapse	experimental (strong)	0.002	BRAF, CYP2C9,HRAS,KRAS,PTGS2
Small cell lung cancer	experimental (strong)	0.002	AKT1, AKT2,BCL2,FHIT,ITGB1,PTGS2
VEGF signaling pathway	experimental (strong)	0.002	AKT1, AKT2,HRAS,KRAS,PTGS2
Adrenergic signaling in cardiomyocytes	experimental (any)	0.002	AKT1, AKT2,BCL2,CALM3,MAPK1,PIK3R1,PPP2R2A,PPP2R5E
Amoebiasis	experimental (any)	0.002	COL1A1, COL3A1,COL5A1,COL5A2,PIK3R1,TLR2,TNF
Natural killer cell mediated cytotoxicity	experimental (any)	0.002	BRAF, HRAS,KRAS,MAPK1,NFATC1,PIK3R1,TNF
Thyroid cancer	experimental (any)	0.002	BRAF, HRAS,KRAS,MAPK1
Acute myeloid leukemia	experimental (strong)	0.003	AKT1, AKT2,BRAF,HRAS,KRAS
B cell receptor signaling pathway	experimental (strong)	0.003	AKT1, AKT2,HRAS,KRAS,NFATC1
Cholinergic synapse	experimental (strong)	0.003	AKT1, AKT2,BCL2,HRAS,KRAS
Fc epsilon RI signaling pathway	experimental (strong)	0.003	AKT1, AKT2,HRAS,KRAS,TNF
GnRH signaling pathway	experimental (strong)	0.003	HRAS, KRAS,MAPK7,MMP14,MMP2
HIF-1 signaling pathway	experimental (strong)	0.003	AKT1, AKT2,BCL2,HK2,IGF1R,SERPINE1
Hepatitis C	experimental (strong)	0.003	AKT1, AKT2,BRAF,HRAS,KRAS,TNF
Insulin signaling pathway	experimental (strong)	0.003	AKT1, AKT2,BRAF,HK2,HRAS,KRAS
Long-term depression	experimental (strong)	0.003	BRAF, HRAS,IGF1R,KRAS

Progesterone-mediated oocyte maturation	experimental (strong)	0.003	AKT1, AKT2,BRAF,IGF1R,KRAS
T cell receptor signaling pathway	experimental (strong)	0.003	AKT1, AKT2,HRAS,KRAS,NFATC1,TNF
Apoptosis	experimental (any)	0.003	AKT1, AKT2,BCL2,PIK3R1,TNF,XIAP
NF-kappa B signaling pathway	experimental (any)	0.003	BCL2, MAP3K7,NFKB2,PTGS2,TNF,XIAP
Ras signaling pathway	experimental (any)	0.003	AKT1, AKT2,CALM3,HRAS,IGF1R,KRAS,MAPK1,PDGFB,PDGFRA,PIK3R1
Colorectal cancer	experimental (strong)	0.004	AKT1, AKT2,BCL2,BRAF,KRAS
Natural killer cell mediated cytotoxicity	experimental (strong)	0.004	BRAF, HRAS,KRAS,NFATC1,TNF
Toxoplasmosis	experimental (strong)	0.004	AKT1, AKT2,BCL2,ITGB1,TLR2,TNF
Carbohydrate digestion and absorption	experimental (any)	0.004	AKT1, AKT2,HK2,PIK3R1
Measles	experimental (any)	0.004	AKT1, AKT2,BBC3,MAP3K7,OAS3,PIK3R1,TLR2
Carbohydrate digestion and absorption	experimental (strong)	0.005	AKT1, AKT2,HK2
Rap1 signaling pathway	experimental (strong)	0.005	AKT1, AKT2,BRAF,HRAS,IGF1R,ITGB1,KRAS
Renal cell carcinoma	experimental (strong)	0.005	AKT1, AKT2,BRAF,HRAS,KRAS
TNF signaling pathway	experimental (strong)	0.006	AKT1, AKT2,MMP14,MMP9,PTGS2,TNF
Long-term potentiation	experimental (any)	0.006	BRAF, CALM3,HRAS,KRAS,MAPK1
Epstein-Barr virus infection	experimental (strong)	0.008	AKT1, AKT2,BCL2,CD44,MDM2,NFKB2
ErbB signaling pathway	experimental (strong)	0.008	AKT1, AKT2,BRAF,HRAS,KRAS
Adherens junction	experimental (any)	0.008	CTNND1, IGF1R,MAP3K7,MAPK1,SMAD3
Chemokine signaling pathway	experimental (any)	0.008	AKT1, AKT2,BRAF,CCR6,HRAS,KRAS,MAPK1,PIK3R1
Regulation of actin cytoskeleton	experimental (strong)	0.009	BRAF, HRAS,ITGB1,ITGB4,KRAS,LIMK1
mTOR signaling pathway	experimental (strong)	0.009	AKT1, AKT2,BRAF,TNF
Type II diabetes mellitus	experimental (any)	0.009	HK2, MAPK1,PIK3R1,TNF
Leishmaniasis	experimental (strong)	0.010	ITGB1, PTGS2,TLR2,TNF
Thyroid hormone signaling pathway	experimental (strong)	0.010	AKT1, AKT2,HRAS,KRAS,MDM2
Chagas disease (American trypanosomiasis)	experimental (strong)	0.011	AKT1, AKT2,SERPINE1,TLR2,TNF
Thyroid cancer	experimental (strong)	0.011	BRAF, HRAS,KRAS
Endocytosis	experimental (any)	0.016	AGAP1, ERBB3,HRAS,IGF1R,MDM2,PDGFRA,RAB22A,SMAD3
Fc gamma R-mediated phagocytosis	experimental (any)	0.016	AKT1, AKT2,LIMK1,MAPK1,PIK3R1
NOD-like receptor signaling pathway	experimental (any)	0.016	CASP5, MAP3K7,MAPK1,TNF
HTLV-I infection	experimental (strong)	0.017	AKT1, AKT2,HRAS,KRAS,NFATC1,NFKB2,TNF
Tuberculosis	experimental (strong)	0.017	AKT1, AKT2,BCL2,TLR2,TNF

Apoptosis	experimental (strong)	0.018	AKT1, AKT2,BCL2,TNF
Malaria	experimental (strong)	0.018	SDC1, TLR2,TNF
Osteoclast differentiation	experimental (strong)	0.018	AKT1, AKT2,NFATC1,NFKB2,TNF
Viral carcinogenesis	experimental (any)	0.018	HIST1H2BG, HRAS,KRAS,MAPK1,MDM2,NFKB2,PIK3R1,YWHAB
Legionellosis	experimental (strong)	0.019	NFKB2, TLR2,TNF
Dopaminergic synapse	experimental (any)	0.019	AKT1, AKT2,CALM3,KCNJ3,PPP2R2A,PPP2R5E
Inflammatory bowel disease (IBD)	experimental (any)	0.019	NFATC1, SMAD3,TLR2,TNF
Prolactin signaling pathway	experimental (strong)	0.021	AKT1, AKT2,HRAS,KRAS
Pancreatic cancer	experimental (strong)	0.023	AKT1, AKT2,BRAF,KRAS
NF-kappa B signaling pathway	experimental (strong)	0.024	BCL2, NFKB2,PTGS2,TNF
Chemokine signaling pathway	experimental (strong)	0.026	AKT1, AKT2,BRAF,HRAS,KRAS
Long-term potentiation	experimental (strong)	0.026	BRAF, HRAS,KRAS
Hypertrophic cardiomyopathy (HCM)	experimental (strong)	0.027	ITGB1, ITGB4,TNF
Tight junction	experimental (strong)	0.027	AKT1, AKT2,HRAS,KRAS
Axon guidance	experimental (strong)	0.028	HRAS, ITGB1,KRAS,LIMK1
Dilated cardiomyopathy	experimental (strong)	0.028	ITGB1, ITGB4,TNF
Arachidonic acid metabolism	experimental (strong)	0.029	CYP2C9, PTGS2
Oocyte meiosis	experimental (any)	0.029	CALM3, IGF1R,MAPK1,PPP2R5E,YWHAB
Aldosterone-regulated sodium reabsorption	experimental (any)	0.031	KRAS, MAPK1,PIK3R1
Leukocyte transendothelial migration	experimental (any)	0.032	CTNND1, ITGB1,MMP2,MMP9,PIK3R1
Pertussis	experimental (any)	0.034	CALM3, ITGB1,MAPK1,TNF
Malaria	experimental (any)	0.037	SDC1, TLR2,TNF
Inflammatory bowel disease (IBD)	experimental (strong)	0.039	NFATC1, TLR2,TNF
Toll-like receptor signaling pathway	experimental (strong)	0.039	AKT1, AKT2,TLR2,TNF
Chemical carcinogenesis	experimental (strong)	0.041	CYP2C9, PTGS2
Adipocytokine signaling pathway	experimental (strong)	0.042	AKT1, AKT2,TNF
Gap junction	experimental (strong)	0.042	HRAS, KRAS,MAPK7
Protein digestion and absorption	experimental (any)	0.042	COL1A1, COL3A1,COL5A1,COL5A2
Influenza A	experimental (any)	0.044	AKT1, AKT2,MAPK1,OAS3,PIK3R1,TNF
Sphingolipid metabolism	experimental (any)	0.044	CERS4, SGPL1,SPTLC2
Axon guidance	experimental (any)	0.048	HRAS, ITGB1,KRAS,LIMK1,MAPK1
Calcium signaling pathway	experimental (any)	0.048	CALM3, ERBB3,NOS1,ORAI2,PDGFRA,PLCD3

	Hippo signaling pathway	experimental (any)	0.048	BBC3, CCN2,PPP2R2A,SERPINE1,SMAD3,YWHAB
	Ovarian steroidogenesis	experimental (any)	0.048	IGF1R, PTGS2,STAR
	Retinol metabolism	experimental (any)	0.048	ADH5, CYP2C9,LRAT
	Tight junction	experimental (any)	0.048	AKT1, AKT2,HRAS,KRAS,PPP2R2A
	Ovarian steroidogenesis	experimental (strong)	0.049	IGF1R, PTGS2
**hsa-miR-127-3p**	Amoebiasis	experimental (any)	0.05	PIK3CG, SERPINB9
	Chemokine signaling pathway	experimental (any)	0.05	GRK2, PIK3CG
	FoxO signaling pathway	experimental (any)	0.05	BCL6, PIK3CG
	Non-alcoholic fatty liver disease (NAFLD)	experimental (any)	0.05	PIK3CG, XBP1
	Protein processing in endoplasmic reticulum	experimental (any)	0.05	SEC31A, XBP1
**hsa-miR-221-3p**	MicroRNAs in cancer	experimental (any)	1.32e-7	BCL2L11,BMF,CASP3,CDC25C,CDK6,CDKN1B,CYP1B1,DDIT4,DICER1,E2F3,FSCN1,MDM2,PDGFA,PTEN,RECK,RHOA,SOCS1,STMN1,TIMP3,ZEB2
	Hepatitis B	experimental (any)	5.71e-6	AKT3,APAF1,CASP3,CDK6,CDKN1B,E2F3,FOS,LAMTOR5,MAPK10,PIK3R1,PTEN,RB1,STAT5A,TBK1,TICAM1,YWHAB
	Pathways in cancer	experimental (any)	8.85e-5	AKT3,ARNT,CASP3,CDK6,CDKN1B,CTNNB1,DVL2,E2F3,ETS1,FOS,KIT,MAPK10,MDM2,MMP2,PDGFA,PIK3R1,PTEN,RAD51,RB1,RHOA,RUNX1,STAT5A,TRAF4
	Prolactin signaling pathway	experimental (any)	7.63e-4	AKT3, ESR1,FOS,FOXO3,MAPK10,PIK3R1,SOCS1,SOCS3,STAT5A
	Epstein-Barr virus infection	experimental (any)	7.70e-4	AKT3,CDKN1B,EIF2AK1,GTF2E1,HSPA1B,ICAM1,LYN,MAPK10,MDM2,PIK3R1,PSMD4,RB1,TBK1,YWHAB,YWHAE
	Chronic myeloid leukemia	experimental (any)	8.83e-4	AKT3, CDK6,CDKN1B,E2F3,MDM2,PIK3R1,RB1,RUNX1,STAT5A
	Influenza A	experimental (any)	8.83e-4	ACTB, ACTG1,AKT3,EIF2AK1,HSPA1B,ICAM1,KPNA2,MAPK10,PIK3R1,SOCS3,TBK1,TICAM1,TNFSF10
	Proteoglycans in cancer	experimental (any)	8.83e-4	ACTB,ACTG1,AKT3,CASP3,CTNNB1,ERBB4,ESR1,FLNA,MDM2,MMP2,PAK1,PIK3R1,PXN,RHOA,TIAM1,TIMP3
	ErbB signaling pathway	predicted (union)	0.001	ABL1,ABL2,AKT2,ARAF,CAMK2D,CBL,CBLB,CDKN1B,CRKL,EGF,EGFR,ELK1,ERBB2,ERBB3,ERBB4,GAB1,GSK3B,HBEGF,MAP2K4,MAPK1,MAPK10,MAPK8,MAPK9,NCK1,NCK2,NRG1,NRG2,NRG3,NRG4,PAK1,PAK2,PAK3,PIK3CA,PIK3CG,PIK3R1,PIK3R3,PLCG1,PRKCA,PRKCB,PTK2,RPS6KB1,SHC3,SOS2,SRC,STAT5B,TGFA
	MicroRNAs in cancer	predicted (union)	0.001	ABL1,ATM,BCL2L11,BCL2L2,BMF,BMI1,BMPR2,BRCA1,CASP3,CCND1,CCND2,CD44,CDK6,CDKN1B,COMMD3-BMI1,CRKL,CYP24A1,DDIT4,DICER1,DNMT3A,DNMT3B,E2F2,E2F3,EGFR,ERBB2,ERBB3,FOXP1,FZD3,GLS,HDAC4,HMOX1,HOXD10,IGF2BP1,IRS1,ITGB3,MAPK1,MDM4,MMP16,NOTCH2,NOTCH3,PDGFA,PDGFRB,PIK3CA,PLCG1,PRKCA,PRKCB,PTEN,RASSF1,RDX,RECK,RHOA,ROCK1,RPTOR,SERPINB5,SIRT1,SLC45A3,SOCS1,SOS2,SOX4,STAT3,STMN1,TGFB2,THBS1,TIMP3,TNN,TNR,TP63,TPM1,TRIM71,UBE2I,ZEB1,ZFPM2
	Cell cycle	experimental (any)	0.002	CDC25C, CDC27,CDK6,CDKN1B,CDKN1C,E2F3,MDM2,RB1,WEE1,YWHAB,YWHAE
	Glioma	experimental (any)	0.002	AKT3, CDK6,E2F3,MDM2,PDGFA,PIK3R1,PTEN,RB1
	Legionellosis	experimental (any)	0.002	APAF1, BNIP3,CASP3,EEF1A1,HSPA1B,NAIP,RAB1A
	Melanoma	experimental (any)	0.002	AKT3, CDK6,E2F3,MDM2,PDGFA,PIK3R1,PTEN,RB1
	Prostate cancer	experimental (any)	0.002	AKT3, CDKN1B,CTNNB1,E2F3,MDM2,PDGFA,PIK3R1,PTEN,RB1
	Small cell lung cancer	experimental (any)	0.002	AKT3, APAF1,CDK6,CDKN1B,E2F3,PIK3R1,PTEN,RB1,TRAF4
	Viral carcinogenesis	experimental (any)	0.002	CASP3, CDK6,CDKN1B,GTF2E1,LYN,MDM2,PIK3R1,PKM,PXN,RB1,RHOA,STAT5A,YWHAB,YWHAE
	Proteoglycans in cancer	predicted (union)	0.003	ACTB,AKT2,ANK2,ARAF,ARHGEF12,CAMK2D,CASP3,CAV2,CBL,CBLB,CCND1,CD44,COL21A1,CTNNB1,CTTN,EGFR,ELK1,ERBB2,ERBB3,ERBB4,ESR1,FASLG,FGF10,FGF12,FGF14,FGF19,FGF2,FGF5,FGF9,FN1,FRS2,FZD1,FZD3,FZD4,FZD5,FZD8,GAB1,HBEGF,HIF1A,HOXD10,HPSE,HPSE2,IGF1,IQGAP1,ITGA2,ITGB3,ITPR1,ITPR2,KDR,MAPK1,MAPK13,MAPK14,MRAS,MSN,PAK1,PDPK1,PIK3CA,PIK3CG,PIK3R1,PIK3R3,PLAUR,PLCE1,PLCG1,PPP1R12A,PPP1R12B,PRKACB,PRKCA,PRKCB,PRKX,PTCH1,PTK2,PTPN11,PXN,RDX,RHOA,ROCK1,RPS6KB1,SDC1,SDC2,SMAD2,SOS2,SRC,STAT3,TGFB2,THBS1,TIAM1,TIMP3,TLR2,TNF,VAV2,VTN,WNT1,WNT10B,WNT2,WNT2B,WNT8B,WNT9B
	Retrograde endocannabinoid signaling	predicted (union)	0.003	ADCY1,ADCY2,ADCY9,CACNA1C,CNR1,FAAH,GABRA1,GABRA3,GABRA4,GABRB1,GABRG1,GABRG3,GABRR1,GABRR2,GNAI2,GNAI3,GNAO1,GNAQ,GNB1,GNB3,GNB4,GNB5,GNG11,GNG12,GNG2,GNG7,GNGT2,GRIA2,GRIA4,GRM1,ITPR1,ITPR2,KCNJ5,KCNJ6,MAPK1,MAPK10,MAPK13,MAPK14,MAPK8,MAPK9,NAPEPLD,PLCB1,PRKACB,PRKCA,PRKCB,PRKX,SLC17A6,SLC17A8

Morphine addiction	predicted (union)	0.004	ADCY1,ADCY2,ADCY9,DRD1,GABBR1,GABBR2,GABRA1,GABRA3,GABRA4,GABRB1,GABRG1,GABRG3,GABRR1,GABRR2,GNAI2,GNAI3,GNAO1,GNAS,GNB1,GNB3,GNB4,GNB5,GNG11,GNG12,GNG2,GNG7,GNGT2,GRK3,GRK5,KCNJ5,KCNJ6,OPRM1,PDE10A,PDE11A,PDE1C,PDE3A,PDE4A,PDE4B,PDE4D,PDE7A,PDE7B,PRKACB,PRKCA,PRKCB,PRKX
Wnt signaling pathway	predicted (union)	0.004	AXIN2,BAMBI,BTRC,CAMK2D,CCND1,CCND2,CHD8,CSNK1A1,CSNK1A1L,CSNK2A1,CTBP2,CTNNB1,CTNNBIP1,CXXC4,DAAM1,DAAM2,DKK2,DVL2,FBXW11,FRAT1,FRAT2,FZD1,FZD3,FZD4,FZD5,FZD8,GSK3B,LRP6,MAP3K7,MAPK10,MAPK8,MAPK9,NFATC2,NFATC3,NFATC4,NKD1,NLK,PLCB1,PPP3CB,PPP3CC,PPP3R1,PPP3R2,PRICKLE2,PRKACB,PRKCA,PRKCB,PRKX,PSEN1,RBX1,RHOA,SENP2,SFRP1,SFRP2,SKP1,TBL1X,TBL1XR1,TCF7L2,VANGL1,VANGL2,WIF1,WNT1,WNT10B,WNT2,WNT2B,WNT8B,WNT9B
Colorectal cancer	experimental (any)	0.004	AKT3, CASP3,CTNNB1,FOS,MAPK10,PIK3R1,RHOA
Focal adhesion	experimental (any)	0.004	ACTB, ACTG1,AKT3,CTNNB1,FLNA,MAPK10,PAK1,PDGFA,PIK3R1,PTEN,PXN,RHOA,ZYX
FoxO signaling pathway	experimental (any)	0.004	AKT3, BCL2L11,BNIP3,CDKN1B,FOXO3,MAPK10,MDM2,PIK3R1,PTEN,TNFSF10
Leukocyte transendothelial migration	experimental (any)	0.004	ACTB, ACTG1,CTNNB1,CXCL12,ICAM1,MMP2,PIK3R1,PXN,RHOA
Measles	experimental (any)	0.004	AKT3, BBC3,CDK6,CDKN1B,EIF2AK1,HSPA1B,PIK3R1,STAT5A,TBK1,TNFSF10
MicroRNAs in cancer	experimental (strong)	0.005	BCL2L11, BMF,CDKN1B,DDIT4,DICER1,MDM2,PTEN,RECK,SOCS1,STMN1,TIMP3,ZEB2
Pancreatic cancer	experimental (any)	0.006	AKT3, CDK6,E2F3,MAPK10,PIK3R1,RAD51,RB1
Pathogenic Escherichia coli infection	experimental (any)	0.007	ACTB, ACTG1,CTNNB1,NCL,RHOA,TUBA1C
MicroRNAs in cancer	predicted (intersection)	0.008	BCL2L11, BMF,BMI1,BRCA1,CCND2,CDK6,CDKN1B,COMMD3-BMI1,CRKL,DICER1,DNMT3B,E2F3,EGFR,ERBB3,FOXP1,FZD3,GLS,IRS1,ITGB3,MAPK1,MDM4,PIK3CA,PLCG1,RASSF1,RDX,RECK,RPTOR,SERPINB5,SIRT1,SLC45A3,STAT3,STMN1,THBS1,TIMP3,TNR,TRIM71,ZFPM2
Osteoclast differentiation	predicted (intersection)	0.008	CAMK4,CREB1,CSF1,FCGR2A,FOS,FOSL2,GAB2,IFNAR2,ITGB3,LILRB4,MAP2K6,MAPK1,MAPK10,MAPK9,MITF,NCF1,NFATC2,PIK3CA,PIK3CG,PIK3R1,PPP3CB,PPP3CC,PPP3R1,SIRPA,SOCS3,SQSTM1,STAT2,SYK,TEC,TGFBR2,TNF,TRAF2,TRAF6
Prolactin signaling pathway	experimental (strong)	0.008	ESR1, FOS,FOXO3,PIK3R1,SOCS1,SOCS3,STAT5A
Non-small cell lung cancer	experimental (any)	0.010	AKT3, CDK6,E2F3,FOXO3,PIK3R1,RB1
Proteoglycans in cancer	predicted (intersection)	0.013	CAV2,CBL,CBLB,EGFR,ERBB3,ERBB4,ESR1,FASLG,FGF10,FGF12,FGF2,FGF5,FGF9,FZD1,FZD3,FZD8,GAB1,HIF1A,HPSE,IGF1,IQGAP1,ITGA2,ITGB3,ITPR2,KDR,MAPK1,MRAS,MSN,PAK1,PIK3CA,PIK3CG,PIK3R1,PLAUR,PLCE1,PLCG1,PPP1R12B,PTK2,PTPN11,RDX,SDC1,SDC2,STAT3,THBS1,TIAM1,TIMP3,TNF,WNT2B,WNT9B
FoxO signaling pathway	predicted (union)	0.016	AKT2,ARAF,ATG12,ATM,BCL2L11,BCL6,BNIP3,CCNB1,CCND1,CCND2,CCNG2,CDK2,CDKN1B,CDKN2B,EGF,EGFR,FASLG,FBXO32,FOXO1,FOXO3,FOXO4,G6PC,GRM1,HOMER1,IGF1,IL7R,INSR,IRS1,IRS4,MAPK1,MAPK10,MAPK13,MAPK14,MAPK8,MAPK9,NLK,PDPK1,PIK3CA,PIK3CG,PIK3R1,PIK3R3,PRKAA2,PRKAB2,PRKAG2,PTEN,RAG1,S1PR1,SETD7,SIRT1,SLC2A4,SMAD2,SOD2,SOS2,STAT3,STK4,TGFB2,TGFBR1,TGFBR2
GABAergic synapse	predicted (union)	0.016	ABAT,ADCY1,ADCY2,ADCY9,CACNA1C,GABBR1,GABBR2,GABRA1,GABRA3,GABRA4,GABRB1,GABRG1,GABRG3,GABRR1,GABRR2,GAD2,GLS,GLUL,GNAI2,GNAI3,GNAO1,GNB1,GNB3,GNB4,GNB5,GNG11,GNG12,GNG2,GNG7,GNGT2,HAP1,KCNJ6,PLCL1,PRKACB,PRKCA,PRKCB,PRKX,SLC38A1,SLC6A1,SLC6A11,SRC
Glutamatergic synapse	predicted (union)	0.016	ADCY1,ADCY2,ADCY9,CACNA1C,DLGAP1,GLS,GLUL,GNAI2,GNAI3,GNAO1,GNAQ,GNAS,GNB1,GNB3,GNB4,GNB5,GNG11,GNG12,GNG2,GNG7,GNGT2,GRIA2,GRIA4,GRIK3,GRIN2A,GRIN2B,GRIN3A,GRK3,GRM1,GRM2,GRM4,HOMER1,ITPR1,ITPR2,MAPK1,PLA2G4A,PLA2G4D,PLCB1,PPP3CB,PPP3CC,PPP3R1,PPP3R2,PRKACB,PRKCA,PRKCB,PRKX,SLC17A6,SLC17A8,SLC1A1,SLC1A2,SLC38A1,TRPC1
Hepatitis B	experimental (strong)	0.016	APAF1, CDKN1B,FOS,PIK3R1,PTEN,RB1,STAT5A,TBK1,TICAM1
Pathways in cancer	experimental (strong)	0.016	ARNT, CDKN1B,DVL2,ETS1,FOS,KIT,MDM2,MMP2,PIK3R1,PTEN,RAD51,RB1,RUNX1,STAT5A
ErbB signaling pathway	predicted (intersection)	0.018	CBL,CBLB,CDKN1B,CRKL,EGFR,ERBB3,ERBB4,GAB1,GSK3B,MAPK1,MAPK10,MAPK9,NRG1,NRG4,PAK1,PAK2,PAK3,PIK3CA,PIK3CG,PIK3R1,PLCG1,PTK2,SHC3
Ras signaling pathway	predicted (intersection)	0.018	CALM1,CSF1,EFNA1,EGFR,EXOC2,FASLG,FGF10,FGF12,FGF2,FGF5,FGF9,GAB1,GAB2,GNB3,GNG11,GNG12,GRIN2A,GRIN2B,IGF1,KDR,KIT,KSR1,MAPK1,MAPK10,MAPK9,MRAS,PAK1,PAK2,PAK3,PDGFD,PIK3CA,PIK3CG,PIK3R1,PLAAT3,PLCE1,PLCG1,PTPN11,RALA,RAPGEF5,RASA4,RASA4B,RASGRF2,RASGRP1,RASSF1,RGL1,SHC3,TEK,TIAM1
Dopaminergic synapse	predicted (union)	0.019	AKT2,ATF2,ATF4,CACNA1C,CALM1,CAMK2D,CREB1,CREB5,DRD1,FOS,GNAI2,GNAI3,GNAL,GNAO1,GNAQ,GNAS,GNB1,GNB3,GNB4,GNB5,GNG11,GNG12,GNG2,GNG7,GNGT2,GRIA2,GRIA4,GRIN2A,GRIN2B,GSK3B,ITPR1,ITPR2,KCNJ5,KCNJ6,KIF5A,KIF5C,MAPK10,MAPK13,MAPK14,MAPK8,MAPK9,PLCB1,PPP2R1A,PPP2R1B,PPP2R2A,PPP2R2B,PPP2R5C,PPP2R5D,PPP2R5E,PPP3CB,PPP3CC,PRKACB,PRKCA,PRKCB,PRKX,SCN1A,SLC18A1,SLC6A3
Type II diabetes mellitus	experimental (any)	0.019	MAPK10, PIK3R1,PKM,SOCS1,SOCS3
FoxO signaling pathway	experimental (strong)	0.022	BCL2L11, BNIP3,CDKN1B,FOXO3,MDM2,PIK3R1,PTEN,TNFSF10
ErbB signaling pathway	experimental (any)	0.022	AKT3, CDKN1B,ERBB4,MAPK10,PAK1,PIK3R1,STAT5A

Hepatitis C	experimental (any)	0.022	AKT3, EIF2AK1,MAPK10,PIK3R1,PPP2R2A,SOCS3,TBK1,TICAM1
Thyroid hormone signaling pathway	experimental (any)	0.022	ACTB, ACTG1,AKT3,ATP2A2,CTNNB1,ESR1,MDM2,PIK3R1
p53 signaling pathway	experimental (any)	0.022	APAF1, BBC3,CASP3,CDK6,MDM2,PTEN
Adherens junction	experimental (any)	0.023	ACTB, ACTG1,CTNNB1,PTPRF,RHOA,SSX2IP
HTLV-I infection	experimental (any)	0.023	AKT3, CDC27,CTNNB1,DVL2,E2F3,ETS1,FOS,ICAM1,MYBL1,PDGFA,PIK3R1,RB1,STAT5A
Non-alcoholic fatty liver disease (NAFLD)	experimental (any)	0.023	AKT3, BCL2L11,CASP3,MAPK10,NDUFB5,NDUFS1,PIK3R1,SOCS3,UQCR10
Viral myocarditis	experimental (any)	0.023	ACTB, ACTG1,CASP3,EIF4G3,ICAM1
Bacterial invasion of epithelial cells	experimental (any)	0.025	ACTB, ACTG1,CTNNB1,PIK3R1,PXN,RHOA
Endometrial cancer	experimental (any)	0.026	AKT3, CTNNB1,FOXO3,PIK3R1,PTEN
Axon guidance	predicted (union)	0.028	ABL1,ABLIM1,ARHGEF12,CFL2,CXCL12,DPYSL2,DPYSL5,EFNA1,EFNA2,EFNB2,EPHA4,EPHA5,EPHA7,EPHA8,GNAI2,GNAI3,GSK3B,MAPK1,NCK1,NCK2,NFATC2,NFATC3,NFATC4,NRP1,PAK1,PAK2,PAK3,PLXNA2,PLXNA3,PLXNB2,PLXNC1,PPP3CB,PPP3CC,PPP3R1,PPP3R2,PTK2,RHOA,ROBO2,ROCK1,SEMA3A,SEMA3C,SEMA3D,SEMA4B,SEMA4C,SEMA4F,SEMA4G,SEMA5A,SEMA6A,SEMA6D,SLIT1,SLIT2,SRGAP1,SRGAP3,UNC5B,UNC5C,UNC5D
Axon guidance	predicted (intersection)	0.034	ABLIM1,CXCL12,EFNA1,EFNB2,EPHA5,GNAI2,GNAI3,GSK3B,MAPK1,NFATC2,NFATC3,NFATC4,PAK1,PAK2,PAK3,PLXNC1,PPP3CB,PPP3CC,PPP3R1,PTK2,ROBO2,SEMA3C,SEMA5A,SEMA6D,SLIT1,SRGAP1,UNC5B,UNC5C,UNC5D
Pathways in cancer	predicted (intersection)	0.034	AR,ARNT,AXIN2,CBL,CBLB,CDH1,CDK6,CDKN1B,CDKN2B,CKS1B,COL4A1,COL4A4,CRKL,CTBP2,CTNNA3,CYCS,DVL2,E2F3,EGFR,FASLG,FGF10,FGF12,FGF2,FGF5,FGF9,FOS,FOXO1,FZD1,FZD3,FZD8,GLI2,GSK3B,HHIP,HIF1A,IGF1,ITGA2,ITGA3,KIT,LAMA4,MAPK1,MAPK10,MAPK9,MITF,MMP1,MSH3,PAX8,PIK3CA,PIK3CG,PIK3R1,PLCG1,PTK2,RAD51,RALA,RASSF1,RUNX1T1,STAT3,TFG,TGFBR2,TRAF2,TRAF6,WNT2B,WNT9B
Rap1 signaling pathway	predicted (intersection)	0.034	ADCY9,ARAP2,CALM1,CDH1,CNR1,CRKL,CSF1,EFNA1,EGFR,FGF10,FGF12,FGF2,FGF5,FGF9,GNAI2,GNAI3,GNAQ,GRIN2A,GRIN2B,IGF1,ITGB3,KDR,KIT,KRIT1,LPAR3,MAGI1,MAGI2,MAP2K6,MAPK1,MRAS,PDGFD,PIK3CA,PIK3CG,PIK3R1,PLCE1,PLCG1,PRKD1,RALA,RAPGEF5,TEK,THBS1,TIAM1,TLN1
T cell receptor signaling pathway	predicted (intersection)	0.034	BCL10,CBL,CBLB,CD28,CD4,FOS,GRAP2,GSK3B,MAPK1,MAPK9,NFATC2,NFATC3,PAK1,PAK2,PAK3,PIK3CA,PIK3CG,PIK3R1,PLCG1,PPP3CB,PPP3CC,PPP3R1,RASGRP1,TEC,TNF
Focal adhesion	predicted (union)	0.036	ACTB,AKT2,ARHGAP5,CAV2,CCND1,CCND2,COL27A1,COL4A1,COL4A3,COL4A4,COL4A5,COL4A6,CRKL,CTNNB1,EGF,EGFR,ELK1,ERBB2,FLT1,FN1,GSK3B,IBSP,IGF1,ITGA1,ITGA10,ITGA11,ITGA2,ITGA3,ITGA4,ITGA9,ITGB3,ITGB8,KDR,LAMA4,LAMB3,LAMC1,MAPK1,MAPK10,MAPK8,MAPK9,MYLK3,PAK1,PAK2,PAK3,PARVA,PARVB,PARVG,PDGFA,PDGFD,PDGFRB,PDPK1,PIK3CA,PIK3CG,PIK3R1,PIK3R3,PPP1R12A,PPP1R12B,PRKCA,PRKCB,PTEN,PTK2,PXN,RAP1A,RAP1B,RAPGEF1,RASGRF1,RELN,RHOA,ROCK1,SHC3,SOS2,SRC,THBS1,THBS2,TLN1,TLN2,TNN,TNR,VAV2,VAV3,VCL,VTN,XIAP,ZYX
Acute myeloid leukemia	experimental (any)	0.037	AKT3, KIT,PIK3R1,RUNX1,STAT5A
Hippo signaling pathway	experimental (any)	0.037	ACTB, ACTG1,AMOT,BBC3,CTNNB1,DVL2,PPP2R2A,YWHAB,YWHAE
Ras signaling pathway	experimental (any)	0.037	AKT3, ETS1,KIT,MAPK10,PAK1,PDGFA,PIK3R1,RAB5C,RHOA,TBK1,TIAM1
TNF signaling pathway	experimental (any)	0.037	AKT3, CASP3,FOS,ICAM1,MAPK10,PIK3R1,SOCS3
Tuberculosis	experimental (any)	0.037	AKT3, APAF1,CASP3,CORO1A,MAPK10,NFYA,NFYC,RAB5C,RHOA
Salmonella infection	experimental (any)	0.038	ACTB, ACTG1,DYNC1H1,FLNA,FOS,MAPK10
FoxO signaling pathway	predicted (intersection)	0.040	ATG12,BCL2L11,CCND2,CDKN1B,CDKN2B,EGFR,FASLG,FBXO32,FOXO1,FOXO3,G6PC,IGF1,IRS1,MAPK1,MAPK10,MAPK9,NLK,PIK3CA,PIK3CG,PIK3R1,PRKAA2,PRKAB2,RAG1,S1PR1,SETD7,SIRT1,SOD2,STAT3,TGFBR2
Bladder cancer	experimental (any)	0.040	E2F3, MDM2,MMP2,RB1
PI3K-Akt signaling pathway	experimental (any)	0.040	AKT3,BCL2L11,CDK6,CDKN1B,DDIT4,FOXO3,KIT,MDM2,PDGFA,PIK3R1,PPP2R2A,PTEN,YWHAB,YWHAE
Chronic myeloid leukemia	experimental (strong)	0.041	CDKN1B, MDM2,PIK3R1,RB1,RUNX1,STAT5A
Legionellosis	experimental (strong)	0.041	APAF1, BNIP3,NAIP,RAB1A
Epithelial cell signaling in Helicobacter pylori infection	experimental (any)	0.046	ATP6V1E1, CASP3,LYN,MAPK10,PAK1

	mRNA surveillance pathway	experimental (any)	0.046	ACIN1, CPSF6,CSTF2T,PELO,PPP2R2A,RNPS1
**hsa-miR-12136**	Adherens junction	predicted (union)	0.005	ACP1,ACTB,ACTN1,ACTN2,ACTN4,AFDN,BAIAP2,CDC42,CDH1,CREBBP,CSNK2A1,CTNNA1,CTNNA2,CTNNA3,CTNNB1,EGFR,EP300,ERBB2,FER,FGFR1,FYN,INSR,IQGAP1,LEF1,LMO7,MAP3K7,MAPK1,MET,NECTIN1,NECTIN3,NECTIN4,NLK,PTPN1,PTPRB,PTPRJ,PTPRM,RAC1,RHOA,SMAD2,SMAD3,SNAI2,SORBS1,SRC,SSX2IP,TCF7,TCF7L1,TCF7L2,TGFBR1,TGFBR2,TJP1,VCL,WAS,WASF1,WASF2,WASF3,WASL,YES1
	MicroRNAs in cancer	predicted (union)	0.005	ABCB1,ABCC1,ABL1,ATM,BAK1,BCL2,BCL2L11,BCL2L2,BMF,BMI1,BMPR2,CASP3,CCND1,CCND2,CCNE2,CCNG1,CD44,CDC25C,CDK6,CDKN1A,CDKN1B,COMMD3-BMI1,CREBBP,CRK,CRKL,CYP24A1,DICER1,DNMT3A,DNMT3B,E2F2,E2F3,EFNA3,EGFR,EP300,ERBB2,ERBB3,FGFR3,FOXP1,FZD3,GLS,HDAC1,HDAC4,HMGA2,HNRNPK,HOXD10,IGF2BP1,IRS1,IRS2,ITGB3,KIF23,KRAS,MAP2K1,MAP2K2,MAPK1,MAPK7,MCL1,MDM4,MET,MMP16,NFKB1,NOTCH1,NOTCH2,NOTCH3,NRAS,PAK4,PDCD4,PDGFA,PDGFRA,PDGFRB,PIK3CA,PIM1,PLAU,PLCG1,PRKCA,PRKCE,PTEN,PTGS2,RDX,RECK,RHOA,ROCK1,RPTOR,SERPINB5,SHC1,SHC4,SIRT1,SLC45A3,SLC7A1,SOS2,SOX4,SPRY2,ST14,STAT3,STMN1,TGFB2,THBS1,TIMP3,TNN,TNR,TP53,TP63,TRIM71,UBE2I,VEGFA,VIM,WNT3,ZEB1
	Hippo signaling pathway	predicted (union)	0.010	ACTB,AFP,AJUBA,AMOT,AREG,AXIN2,BIRC5,BMP2,BMP5,BMP6,BMP7,BMP8A,BMP8B,BMPR1A,BMPR1B,BMPR2,BTRC,CCN2,CCND1,CCND2,CDH1,CRB1,CSNK1D,CSNK1E,CTNNA1,CTNNA2,CTNNA3,CTNNB1,DLG1,DLG2,DLG3,DLG4,DVL2,DVL3,FBXW11,FGF1,FRMD6,FZD1,FZD10,FZD2,FZD3,FZD4,FZD5,FZD6,FZD7,FZD8,GDF6,GDF7,GLI2,GSK3B,LATS1,LATS2,LEF1,LIMD1,LLGL1,LLGL2,MOB1A,MOB1B,MPP5,NF2,NKD1,PARD6B,PARD6G,PATJ,PPP1CA,PPP1CB,PPP1CC,PPP2CA,PPP2CB,PPP2R1B,PPP2R2A,PPP2R2B,PPP2R2C,PRKCI,RASSF6,SAV1,SERPINE1,SMAD1,SMAD2,SMAD3,SMAD7,SNAI2,SOX2,STK3,TCF7,TCF7L1,TCF7L2,TEAD2,TGFB2,TGFBR1,TGFBR2,TP53BP2,TP73,WNT10B,WNT11,WNT2B,WNT3,WNT5B,WNT8B,WNT9B,WTIP,WWC1,WWTR1,YAP1,YWHAB,YWHAE,YWHAG,YWHAH,YWHAZ
	Hippo signaling pathway	predicted (intersection)	0.016	ACTB,AFP,AJUBA,AMOT,AREG,AXIN2,BMP2,BMP5,BMP8B,BMPR1A,BMPR2,BTRC,CCND2,CDH1,CRB1,CSNK1E,CTNNA1,CTNNA2,CTNNA3,CTNNB1,DLG1,DLG2,DLG4,DVL2,FGF1,FRMD6,FZD1,FZD10,FZD3,FZD4,FZD6,FZD7,FZD8,GDF6,GDF7,GSK3B,LATS1,LATS2,LEF1,LIMD1,MOB1A,MOB1B,MPP5,NKD1,PARD6B,PPP1CB,PPP1CC,PPP2CA,PPP2R1B,PPP2R2A,PPP2R2B,PPP2R2C,PRKCI,RASSF6,SAV1,SMAD1,SMAD2,SMAD3,SOX2,TCF7,TCF7L2,TEAD2,TGFB2,TGFBR1,TGFBR2,TP53BP2,WTIP,WWC1,WWTR1,YAP1,YWHAB,YWHAG,YWHAH,YWHAZ
	Ubiquitin mediated proteolysis	predicted (union)	0.019	AIRE,ANAPC10,ANAPC13,ANAPC5,ANAPC7,BIRC3,BIRC6,BTRC,CBL,CBLB,CDC16,CDC23,CDC27,CDC34,COP1,CUL3,CUL4A,CUL4B,CUL5,DDB1,DET1,ELOB,ELOC,FBXO4,FBXW11,FBXW7,FBXW8,HERC2,HERC3,HERC4,ITCH,KLHL9,MAP3K1,MGRN1,MID1,NEDD4,PIAS1,PIAS2,PIAS3,PIAS4,PML,PPIL2,PRKN,PRPF19,RBX1,RCHY1,RHOBTB1,RHOBTB2,RNF7,SAE1,SKP1,SKP2,SMURF1,SMURF2,TRAF6,TRIM32,TRIM37,TRIP12,UBA2,UBA3,UBA6,UBE2A,UBE2B,UBE2C,UBE2D1,UBE2D2,UBE2D3,UBE2D4,UBE2E1,UBE2E2,UBE2E3,UBE2F,UBE2G1,UBE2G2,UBE2H,UBE2I,UBE2J1,UBE2K,UBE2L3,UBE2N,UBE2O,UBE2Q1,UBE2Q2,UBE2QL1,UBE2R2,UBE2W,UBE2Z,UBE3A,UBE3B,UBE3C,UBE4A,UBE4B,UBOX5,UBR5,VHL,WWP1,WWP2,XIAP
	Protein processing in endoplasmic reticulum	predicted (union)	0.021	AMFR,ATF6,ATF6B,BAG2,BAK1,BAX,BCL2,CALR,CANX,CAPN1,CAPN2,CKAP4,CRYAA,DAD1,DERL1,DERL2,DERL3,DNAJA2,DNAJB1,DNAJB12,DNAJC10,DNAJC3,DNAJC5,DNAJC5G,EDEM1,EDEM3,EIF2AK1,EIF2AK2,EIF2AK3,EIF2S1,ERLEC1,ERN1,ERO1A,ERO1B,ERP29,GANAB,HSP90AA1,HSP90B1,HSPA1B,HSPA4L,HSPA5,HSPA6,HSPH1,LMAN1,LMAN2,MAN1A1,MAN1A2,MAN1C1,MAP2K7,MAPK8,MAPK9,MARCH6,MBTPS1,MBTPS2,NFE2L2,NPLOC4,OS9,P4HB,PDIA3,PDIA4,PDIA6,PLAA,PPP1R15A,PRKN,RAD23B,RBX1,RNF185,RPN1,RPN2,SAR1A,SAR1B,SEC23B,SEC24A,SEC24B,SEC24C,SEC31A,SEC31B,SEC61A1,SEC61G,SEC62,SEC63,SEL1L,SELENOS,SKP1,SSR1,SSR3,STT3A,STT3B,SVIP,TRAM1,TUSC3,UBE2D1,UBE2D2,UBE2D3,UBE2D4,UBE2E1,UBE2E2,UBE2E3,UBE2G1,UBE2G2,UBE2J1,UBE4B,UBQLN1,UBQLN2,UBQLN3,UBQLN4,UFD1,UGGT1,UGGT2,VCP,XBP1,YOD1
	TGF-beta signaling pathway	predicted (intersection)	0.028	ACVR1,ACVR1B,ACVR1C,ACVR2A,ACVR2B,BAMBI,BMP2,BMP5,BMP8B,BMPR1A,BMPR2,CDKN2B,E2F4,EP300,FST,GDF6,GDF7,ID3,ID4,INHBA,INHBC,MAPK1,NOG,PPP2CA,PPP2R1B,RBL1,RHOA,RPS6KB1,SKP1,SMAD1,SMAD2,SMAD3,SMAD5,SMAD6,SMURF1,SMURF2,SP1,TFDP1,TGFB2,TGFBR1,TGFBR2,THBS1,ZFYVE16
	Bacterial invasion of epithelial cells	predicted (union)	0.035	ACTB,ARHGAP10,ARHGEF26,ARPC1B,ARPC2,ARPC3,ARPC4,ARPC5,CAV2,CAV3,CBL,CBLB,CD2AP,CDC42,CDH1,CLTB,CLTC,CRK,CRKL,CTNNA1,CTNNA2,CTNNA3,CTNNB1,CTTN,DNM3,DOCK1,FN1,GAB1,ITGB1,MET,PIK3CA,PIK3CB,PIK3CG,PIK3R1,PIK3R3,PTK2,PXN,RAC1,RHOA,RHOG,SEPTIN11,SEPTIN12,SEPTIN3,SEPTIN6,SEPTIN8,SHC1,SHC3,SHC4,SRC,VCL,WAS,WASF1,WASF2,WASL
	Endometrial cancer	predicted (intersection)	0.039	AKT2,ARAF,AXIN2,CASP9,CDH1,CTNNA1,CTNNA2,CTNNA3,CTNNB1,EGF,ELK1,ERBB2,FOXO3,GSK3B,KRAS,LEF1,MAP2K2,MAPK1,NRAS,PDPK1,PIK3CA,PIK3CG,PIK3R1,PIK3R3,PTEN,SOS2,TCF7,TCF7L2
	Adherens junction	predicted (intersection)	0.040	ACTB,ACTN2,AFDN,BAIAP2,CDH1,CTNNA1,CTNNA2,CTNNA3,CTNNB1,EP300,ERBB2,FER,INSR,IQGAP1,LEF1,LMO7,MAP3K7,MAPK1,MET,NECTIN1,NECTIN3,NLK,PTPN1,PTPRB,PTPRJ,RAC1,RHOA,SMAD2,SMAD3,TCF7,TCF7L2,TGFBR1,TGFBR2,TJP1,VCL,WASF1,WASL,YES1
	FoxO signaling pathway	predicted (intersection)	0.040	AKT2,ARAF,ATG12,ATM,BCL2L11,BNIP3,CCNB1,CCND2,CDK2,CDKN2B,CSNK1E,EGF,EP300,FBXO32,FOXO3,G6PC,G6PC3,GADD45G,GRM1,HOMER1,IGF1,INSR,IRS1,IRS4,KLF2,KRAS,MAP2K2,MAPK1,MAPK14,MAPK8,MAPK9,NLK,NRAS,PDPK1,PIK3CA,PIK3CG,PIK3R1,PIK3R3,PLK2,PLK4,PRKAA1,PRKAA2,PRKAB1,PRKAB2,PTEN,RAG1,S1PR1,SETD7,SGK1,SIRT1,SKP2,SMAD2,SMAD3,SOD2,SOS2,STAT3,STK4,TGFB2,TGFBR1,TGFBR2
	Renal cell carcinoma	predicted (intersection)	0.040	AKT2,ARAF,ARNT,CRK,EGLN1,EGLN3,ELOC,EP300,EPAS1,FLCN,GAB1,HIF1A,KRAS,MAP2K2,MAPK1,MET,NRAS,PAK1,PAK2,PAK3,PAK6,PIK3CA,PIK3CG,PIK3R1,PIK3R3,PTPN11,RAC1,RAP1A,RAPGEF1,SOS2,TGFA,TGFB2,VHL
**hsa-miR-22-3p**	MicroRNAs in cancer	experimental (any)	8.08e-5	CDK6, CDKN1A,DDIT4,E2F2,ERBB2,ERBB3,FOXP1,GRB2,HDAC4,PTEN,SIRT1
	Pathways in cancer	experimental (any)	8.08e-5	AKT1,CDK6,CDKN1A,CSF1R,CYCS,E2F2,ERBB2,GRB2,HIF1A,MAX,MECOM,PTEN,SLC2A1,TCF7,WNT1

Endocytosis	predicted (intersection)	2.54e-4	ACAP3,AGAP1,AGAP2,AGAP3,AP2A2,ARAP3,ARFGAP2,ARRB1,ASAP1,CAV3,CBL,CSF1R,CXCR2,DNM3,EGF,EGFR,EHD1,EHD3,EPN2,ERBB3,ERBB4,FGFR2,FGFR3,GIT1,GRK1,HLA-E,HSPA1B,IL2RB,LDLRAP1,MVB12B,PARD6G,PIP5K1A,PIP5K1C,PML,PRKCI,PSD2,PSD3,PSD4,RAB11FIP3,RAB11FIP5,RAB22A,RAB5B,RAB5C,RET,RHOA,RUFY1,SH3GLB1,SMAD3,SMAD7,SMAP1,SMURF1,SRC,STAM2,TFRC,TGFBR1,TRAF6,USP8,VPS37C,ZFYVE16
Hepatitis B	experimental (any)	4.05e-4	AKT1, CCNA2,CDK6,CDKN1A,CYCS,E2F2,GRB2,PTEN,YWHAZ
Prostate cancer	experimental (any)	8.24e-4	AKT1, CDKN1A,E2F2,ERBB2,GRB2,PTEN,TCF7
Cell cycle	experimental (any)	8.75e-4	BUB1B, CCNA2,CDK6,CDKN1A,E2F2,PLK1,RBL1,YWHAZ
Estrogen signaling pathway	experimental (any)	9.68e-4	AKT1, ESR1,FKBP5,GRB2,HSPA1B,PRKACA,SP1
Glioma	experimental (any)	9.68e-4	AKT1, CDK6,CDKN1A,E2F2,GRB2,PTEN
Chronic myeloid leukemia	experimental (any)	0.001	AKT1, CDK6,CDKN1A,E2F2,GRB2,MECOM
Epstein-Barr virus infection	experimental (any)	0.001	AKT1, CCNA2,CDKN1A,CSNK2A1,HDAC4,HSPA1B,NUP214,PRKACA,YWHAZ
HIF-1 signaling pathway	experimental (any)	0.001	AKT1, CDKN1A,ERBB2,HIF1A,PDHA1,SLC2A1,TFRC
Proteoglycans in cancer	experimental (any)	0.001	AKT1, CDKN1A,ERBB2,ERBB3,ESR1,GRB2,HIF1A,PRKACA,TIAM1,WNT1
Alcoholism	experimental (any)	0.002	BDNF, GRB2,H3F3B,HDAC4,HDAC6,MAOA,NTRK2,PRKACA
Endometrial cancer	experimental (any)	0.002	AKT1, ERBB2,GRB2,PTEN,TCF7
Non-small cell lung cancer	experimental (any)	0.002	AKT1, CDK6,E2F2,ERBB2,GRB2
Small cell lung cancer	experimental (any)	0.002	AKT1, CDK6,CYCS,E2F2,MAX,PTEN
Viral carcinogenesis	experimental (any)	0.002	CCNA2, CDK6,CDKN1A,GRB2,HDAC4,HDAC6,PRKACA,RBL1,YWHAZ
Neurotrophin signaling pathway	predicted (union)	0.003	ABL1,AKT2,BCL2,BDNF,CALM1,CALM3,CAMK2B,CAMK2D,CAMK2G,CAMK4,CDC42,CRK,FASLG,FOXO3,GSK3B,IRAK1,IRAK2,IRAK3,IRAK4,IRS1,KRAS,MAP2K1,MAP2K2,MAP3K1,MAP3K3,MAP3K5,MAPK1,MAPK10,MAPK11,MAPK13,MAPK14,MAPK8,MAPK9,MAPKAPK2,NFKBIA,NGFR,NRAS,NTRK2,NTRK3,PDPK1,PIK3CA,PIK3CG,PIK3R1,PIK3R3,PIK3R5,PLCG1,PRDM4,PSEN1,PTPN11,RAC1,RAP1A,RAP1B,RAPGEF1,RELA,RHOA,RPS6KA1,RPS6KA2,RPS6KA3,RPS6KA6,SH2B1,SH2B3,SHC1,SHC2,SHC3,SORT1,TP53,TP73,TRAF6
Wnt signaling pathway	predicted (union)	0.003	BAMBI,BTRC,CAMK2B,CAMK2D,CAMK2G,CCND1,CHD8,CREBBP,CSNK1A1,CSNK1E,CSNK2A1,CTBP1,CUL1,CXXC4,DKK2,DVL3,EP300,FOSL1,FRAT1,FRAT2,FZD3,FZD4,FZD5,FZD6,FZD8,FZD9,GPC4,GSK3B,LEF1,LRP6,MAP3K7,MAPK10,MAPK8,MAPK9,NFATC2,NFATC3,NFATC4,NKD1,NKD2,NLK,PLCB3,PPP3CB,PPP3R1,PPP3R2,PRICKLE2,PRKACA,PRKCA,PSEN1,RAC1,RBX1,RHOA,ROCK2,RUVBL1,SFRP1,SFRP2,SKP1,SMAD3,SOX17,TBL1X,TBL1XR1,TBL1Y,TCF7,TCF7L1,TCF7L2,TP53,VANGL1,VANGL2,WNT1,WNT11,WNT2B,WNT3,WNT3A,WNT4,WNT7B,WNT8A,WNT8B,WNT9A,WNT9B
Melanoma	experimental (any)	0.005	AKT1,CDK6,CDKN1A,E2F2,PTEN
Thyroid hormone signaling pathway	experimental (any)	0.006	AKT1, ESR1,HIF1A,NCOA1,PRKACA,SLC2A1
Hippo signaling pathway	experimental (any)	0.007	BMP6, BMP7,BMPR1B,TCF7,WNT1,WWC1,YWHAZ
TGF-beta signaling pathway	experimental (any)	0.008	BMP6, BMP7,BMPR1B,RBL1,SP1
ErbB signaling pathway	predicted (union)	0.010	ABL1,ABL2,AKT2,ARAF,BTC,CAMK2B,CAMK2D,CAMK2G,CBL,CBLB,CDKN1A,CRK,EGF,EGFR,EIF4EBP1,ERBB3,ERBB4,EREG,GSK3B,KRAS,MAP2K1,MAP2K2,MAP2K4,MAPK1,MAPK10,MAPK8,MAPK9,NCK1,NCK2,NRAS,NRG1,NRG2,NRG4,PAK1,PAK4,PAK6,PIK3CA,PIK3CG,PIK3R1,PIK3R3,PIK3R5,PLCG1,PRKCA,RPS6KB2,SHC1,SHC2,SHC3,SRC,STAT5A
FoxO signaling pathway	experimental (any)	0.010	AKT1, CDKN1A,GRB2,PLK1,PTEN,SIRT1
Adherens junction	predicted (union)	0.011	AFDN,BAIAP2,CDC42,CDH1,CREBBP,CSNK2A1,EGFR,EP300,FARP2,FER,FGFR1,INSR,LEF1,MAP3K7,MAPK1,MET,NECTIN1,NECTIN3,NECTIN4,NLK,PARD3,PTPN1,PTPRJ,RAC1,RHOA,SMAD2,SMAD3,SNAI1,SORBS1,SRC,SSX2IP,TCF7,TCF7L1,TCF7L2,TGFBR1,TGFBR2,TJP1,VCL,WASF1,WASF2,WASF3,WASL
Axon guidance	predicted (union)	0.011	ABL1,ABLIM1,ABLIM3,ARHGEF12,CDC42,CFL2,CXCL12,DCC,DPYSL2,DPYSL5,EFNA5,EFNB2,EPHA3,EPHA4,EPHA5,EPHB2,FES,GNAI3,GSK3B,KRAS,LIMK2,MAPK1,MET,NCK1,NCK2,NFATC2,NFATC3,NFATC4,NRAS,NRP1,NTN1,NTNG1,NTNG2,PAK1,PAK4,PAK6,PLXNA1,PLXNA2,PLXNA3,PLXNB1,PLXNB2,PPP3CB,PPP3R1,PPP3R2,RAC1,RASA1,RGS3,RHOA,RND1,ROCK1,ROCK2,SEMA3A,SEMA3D,SEMA4D,SEMA4F,SEMA5A,SEMA6B,SEMA6D,SEMA7A,SLIT1,SLIT2,SRGAP1,SRGAP3,UNC5B,UNC5C,UNC5D
Colorectal cancer	predicted (union)	0.011	AKT2,APPL1,ARAF,BCL2,BIRC5,CASP9,CCND1,CYCS,DCC,FOS,GSK3B,KRAS,LEF1,MAP2K1,MAPK1,MAPK10,MAPK8,MAPK9,MLH1,MSH2,MSH3,MSH6,PIK3CA,PIK3CG,PIK3R1,PIK3R3,PIK3R5,RAC1,RHOA,SMAD2,SMAD3,TCF7,TCF7L1,TCF7L2,TGFBR1,TGFBR2,TP53

Hippo signaling pathway	predicted (union)	0.011	AMOT,BIRC5,BMP2,BMP7,BMP8A,BMPR1A,BMPR1B,BMPR2,BTRC,CCND1,CDH1,CRB1,CRB2,CSNK1D,CSNK1E,DLG1,DLG2,DLG3,DVL3,FRMD6,FZD3,FZD4,FZD5,FZD6,FZD8,FZD9,GDF6,GDF7,GLI2,GSK3B,ID1,LEF1,LIMD1,LLGL2,MOB1A,MOB1B,MPP5,NF2,NKD1,PARD3,PARD6G,PPP1CA,PPP1CC,PPP2CA,PPP2R1A,PPP2R1B,PPP2R2B,PPP2R2C,PRKCI,SERPINE1,SMAD1,SMAD2,SMAD3,SMAD7,SOX2,TCF7,TCF7L1,TCF7L2,TGFBR1,TGFBR2,TP53BP2,TP73,WNT1,WNT11,WNT2B,WNT3,WNT3A,WNT4,WNT7B,WNT8A,WNT8B,WNT9A,WNT9B,WTIP,WWC1,WWTR1,YAP1,YWHAB,YWHAG,YWHAZ
MAPK signaling pathway	predicted (union)	0.011	AKT2,ARRB1,ARRB2,ATF2,BDNF,CACNA1B,CACNA1C,CACNA1G,CACNA1I,CACNA1S,CACNA2D1,CACNA2D4,CACNB1,CACNB3,CACNB4,CACNG3,CACNG6,CACNG8,CDC25B,CDC42,CRK,DUSP10,DUSP16,DUSP2,DUSP3,DUSP4,DUSP5,DUSP6,EGF,EGFR,ELK4,FAS,FASLG,FGF10,FGF12,FGF2,FGF23,FGFR1,FGFR2,FGFR3,FLNC,FOS,GADD45G,GNA12,GNG12,HSPA1B,IL1R1,JMJD7-PLA2G4B,KRAS,MAP2K1,MAP2K2,MAP2K4,MAP2K6,MAP3K1,MAP3K11,MAP3K12,MAP3K13,MAP3K20,MAP3K3,MAP3K5,MAP3K6,MAP3K7,MAP4K2,MAP4K4,MAPK1,MAPK10,MAPK11,MAPK13,MAPK14,MAPK8,MAPK8IP3,MAPK9,MAPKAPK2,MAPKAPK5,MAPT,MAX,MECOM,MEF2C,MRAS,NF1,NFATC3,NLK,NRAS,NTRK2,PAK1,PDGFB,PDGFRB,PLA2G4A,PLA2G4F,PPM1A,PPM1B,PPP3CB,PPP3R1,PPP3R2,PPP5C,PRKACA,PRKCA,RAC1,RAP1A,RAP1B,RAPGEF2,RASA1,RASA2,RASGRF1,RASGRF2,RASGRP2,RELA,RPS6KA1,RPS6KA2,RPS6KA3,RPS6KA6,SRF,STK4,STMN1,TAB1,TAOK1,TGFBR1,TGFBR2,TNFRSF1A,TP53,TRAF2,TRAF6
MicroRNAs in cancer	predicted (union)	0.011	ABL1,BAK1,BCL2,BCL2L2,BMF,BMPR2,CCND1,CCNG1,CDC25B,CDK6,CDKN1A,CREBBP,CRK,DDIT4,DICER1,DNMT3A,E2F2,EGFR,EP300,ERBB3,EZR,FGFR3,FOXP1,FZD3,GLS,HDAC1,HDAC4,HMGA2,HMOX1,HNRNPK,IGF2BP1,IRS1,IRS2,ITGA5,ITGB3,KRAS,MAP2K1,MAP2K2,MAPK1,MCL1,MET,MMP16,NOTCH2,NOTCH4,NRAS,PAK4,PDGFB,PDGFRB,PIK3CA,PIM1,PLCG1,PRKCA,PRKCE,PTEN,RHOA,ROCK1,RPTOR,SHC1,SIRT1,SLC45A3,SLC7A1,SOCS1,SOX4,STAT3,STMN1,TIMP3,TNN,TNR,TP53,TP63,TPM1,TRIM71,UBE2I,WNT3,WNT3A,ZEB1,ZFPM2
Morphine addiction	predicted (union)	0.011	ADCY1,ADCY2,ADCY5,ADCY9,ADORA1,ARRB1,ARRB2,CACNA1B,GABBR1,GABBR2,GABRA1,GABRA4,GABRB1,GABRB3,GABRD,GABRG1,GABRR1,GABRR2,GNAI3,GNAO1,GNAS,GNB4,GNB5,GNG11,GNG12,GNG4,GNG5,GNG7,GRK5,KCNJ3,KCNJ5,KCNJ6,KCNJ9,OPRM1,PDE10A,PDE11A,PDE1A,PDE1B,PDE2A,PDE3A,PDE3B,PDE4A,PDE4B,PDE4D,PDE7A,PDE7B,PRKACA,PRKCA,SLC32A1
ErbB signaling pathway	experimental (any)	0.011	AKT1, CDKN1A,ERBB2,ERBB3,GRB2
Endocytosis	predicted (union)	0.012	ACAP3,ADRB2,AGAP1,AGAP2,AGAP3,AP2A2,AP2M1,ARAP3,ARFGAP1,ARFGAP2,ARRB1,ARRB2,ASAP1,ASAP2,CAV3,CBL,CBLB,CDC42,CHMP3,CLTB,CSF1R,CXCR1,CXCR2,DNM3,EGF,EGFR,EHD1,EHD3,EHD4,EPN2,EPN3,EPS15,ERBB3,ERBB4,FGFR2,FGFR3,GIT1,GRK1,GRK5,HLA-E,HSPA1B,IL2RB,ITCH,KDR,LDLRAP1,MET,MVB12B,NEDD4,PARD3,PARD6G,PIP5K1A,PIP5K1C,PIP5KL1,PLD1,PML,PRKCI,PSD2,PSD3,PSD4,RAB11A,RAB11B,RAB11FIP1,RAB11FIP3,RAB11FIP5,RAB22A,RAB31,RAB5B,RAB5C,RBSN,RET,RHOA,RNF103-CHMP3,RNF41,RUFY1,SH3GLB1,SH3GLB2,SH3KBP1,SMAD2,SMAD3,SMAD6,SMAD7,SMAP1,SMURF1,SRC,STAM2,TFRC,TGFBR1,TGFBR2,TRAF6,USP8,VPS37B,VPS37C,VPS45,VPS4A,VTA1,ZFYVE16
mTOR signaling pathway	experimental (any)	0.014	AKT1, DDIT4,HIF1A,PTEN
Hippo signaling pathway	predicted (intersection)	0.017	AMOT,BMP2,BMP8A,BMPR1A,BMPR2,BTRC,CDH1,CRB2,CSNK1D,CSNK1E,DLG3,DVL3,FRMD6,FZD5,FZD6,FZD8,GDF6,GDF7,LEF1,LIMD1,MOB1A,MOB1B,NF2,NKD1,PARD6G,PPP2R1B,PRKCI,SERPINE1,SMAD3,SMAD7,TCF7,TCF7L2,TGFBR1,TP73,WNT1,WNT3,WNT4,WNT8B,WNT9A,WNT9B,WWC1,YAP1,YWHAG,YWHAZ
Long-term potentiation	predicted (union)	0.017	ADCY1,ARAF,CACNA1C,CALM1,CALM3,CAMK2B,CAMK2D,CAMK2G,CAMK4,CREBBP,EP300,GNAQ,GRIA1,GRIN1,GRIN2A,GRIN2B,GRIN2D,GRM1,KRAS,MAP2K1,MAP2K2,MAPK1,NRAS,PLCB3,PPP1CA,PPP1CC,PPP1R1A,PPP3CB,PPP3R1,PPP3R2,PRKACA,PRKCA,RAP1A,RAP1B,RAPGEF3,RPS6KA1,RPS6KA2,RPS6KA3,RPS6KA6
Prolactin signaling pathway	predicted (union)	0.017	AKT2,CCND1,CISH,ELF5,ESR1,ESR2,FOS,FOXO3,GALT,GCK,GSK3B,KRAS,MAP2K1,MAP2K2,MAPK1,MAPK10,MAPK11,MAPK13,MAPK14,MAPK8,MAPK9,NRAS,PIK3CA,PIK3CG,PIK3R1,PIK3R3,PIK3R5,RELA,SHC1,SHC2,SHC3,SOCS1,SOCS2,SOCS6,SRC,STAT1,STAT3,STAT5A,TNFRSF11A
Prostate cancer	predicted (union)	0.018	AKT2,AR,ARAF,BCL2,CASP9,CCND1,CDKN1A,CREB1,CREB3,CREB3L2,CREB3L3,CREB5,CREBBP,E2F2,EGF,EGFR,EP300,FGFR1,FGFR2,FOXO1,GSK3B,HSP90B1,IGF1,KRAS,LEF1,MAP2K1,MAP2K2,MAPK1,NFKBIA,NRAS,PDGFB,PDGFC,PDGFD,PDGFRB,PDPK1,PIK3CA,PIK3CG,PIK3R1,PIK3R3,PIK3R5,PTEN,RELA,TCF7,TCF7L1,TCF7L2,TP53
Endocytosis	experimental (any)	0.018	CSF1R, CXCR2,ERBB3,HSPA1B,RAB5B,RBSN,TFRC
MAPK signaling pathway	experimental (any)	0.019	AKT1, BDNF,GRB2,HSPA1B,MAX,MECOM,NTRK2,PRKACA
Thyroid cancer	predicted (union)	0.020	CCND1,CDH1,KRAS,LEF1,MAP2K1,MAP2K2,MAPK1,NRAS,PAX8,PPARG,RET,RXRA,TCF7,TCF7L1,TCF7L2,TFG,TP53,TPM3,TPR
mTOR signaling pathway	predicted (union)	0.020	AKT2,CAB39,CAB39L,DDIT4,EIF4E,EIF4E2,EIF4EBP1,IGF1,IRS1,MAPK1,MLST8,PDPK1,PIK3CA,PIK3CG,PIK3R1,PIK3R3,PIK3R5,PRKAA2,PRKCA,PTEN,RHEB,RPS6KA1,RPS6KA2,RPS6KA3,RPS6KA6,RPS6KB2,RPTOR,RRAGC,RRAGD,STRADA,TSC1,ULK1,ULK2,ULK3
Pancreatic cancer	experimental (any)	0.020	AKT1, CDK6,E2F2,ERBB2
Renal cell carcinoma	experimental (any)	0.020	AKT1, GRB2,HIF1A,SLC2A1
p53 signaling pathway	experimental (any)	0.020	CDK6, CDKN1A,CYCS,PTEN
Adherens junction	experimental (any)	0.021	CSNK2A1, ERBB2,SNAI1,TCF7
Chronic myeloid leukemia	predicted (union)	0.026	ABL1,AKT2,ARAF,BCL2L1,BCR,CBL,CBLB,CCND1,CDK6,CDKN1A,CRK,CTBP1,E2F2,HDAC1,HDAC2,KRAS,MAP2K1,MAP2K2,MAPK1,MECOM,NFKBIA,NRAS,PIK3CA,PIK3CG,PIK3R1,PIK3R3,PIK3R5,PTPN11,RELA,RUNX1,SHC1,SHC2,SHC3,SMAD3,STAT5A,TGFBR1,TGFBR2,TP53

Circadian entrainment	predicted (union)	0.026	ADCY1,ADCY2,ADCY5,ADCY9,ADCYAP1R1,CACNA1C,CACNA1G,CACNA1I,CALM1,CALM3,CAMK2B,CAMK2D,CAMK2G,CREB1,FOS,GNAI3,GNAO1,GNAQ,GNAS,GNB4,GNB5,GNG11,GNG12,GNG4,GNG5,GNG7,GRIA1,GRIN1,GRIN2A,GRIN2B,GRIN2D,GUCY1A1,GUCY1A2,GUCY1B1,KCNJ3,KCNJ5,KCNJ6,KCNJ9,MAPK1,NOS1,NOS1AP,PER1,PER2,PER3,PLCB3,PRKACA,PRKCA,PRKG1,PRKG2,RASD1,RYR1
Estrogen signaling pathway	predicted (union)	0.026	ADCY1,ADCY2,ADCY5,ADCY9,AKT2,ATF2,CALM1,CALM3,CREB1,CREB3,CREB3L2,CREB3L3,CREB5,EGFR,ESR1,ESR2,FKBP4,FKBP5,FOS,GABBR1,GABBR2,GNAI3,GNAO1,GNAQ,GNAS,GPER1,GRM1,HSP90B1,HSPA1B,KCNJ3,KCNJ5,KCNJ6,KCNJ9,KRAS,MAP2K1,MAP2K2,MAPK1,NRAS,OPRM1,PIK3CA,PIK3CG,PIK3R1,PIK3R3,PIK3R5,PLCB3,PRKACA,SHC1,SHC2,SHC3,SP1,SRC
Insulin signaling pathway	predicted (union)	0.026	ACACB,AKT2,ARAF,CALM1,CALM3,CBL,CBLB,CRK,EIF4E,EIF4E2,EIF4EBP1,FASN,FOXO1,GCK,GSK3B,GYS2,HKDC1,INPP5K,INSR,IRS1,IRS2,IRS4,KRAS,MAP2K1,MAP2K2,MAPK1,MAPK10,MAPK8,MAPK9,NRAS,PCK1,PDE3A,PDE3B,PDPK1,PHKA2,PHKG2,PIK3CA,PIK3CG,PIK3R1,PIK3R3,PIK3R5,PKLR,PPARGC1A,PPP1CA,PPP1CC,PPP1R3B,PPP1R3D,PPP1R3E,PRKAA2,PRKAB1,PRKAB2,PRKACA,PRKAR1A,PRKAR2A,PRKCI,PTPN1,PYGB,RAPGEF1,RHEB,RHOQ,RPS6KB2,RPTOR,SHC1,SHC2,SHC3,SOCS1,SOCS2,SORBS1,TSC1
Adrenergic signaling in cardiomyocytes	predicted (union)	0.027	ADCY1,ADCY2,ADCY5,ADCY9,ADRA1A,ADRA1B,ADRA1D,ADRB2,AKT2,ATF2,ATP1A3,ATP1B4,ATP2B1,ATP2B4,BCL2,CACNA1C,CACNA1S,CACNA2D1,CACNA2D4,CACNB1,CACNB3,CACNB4,CACNG3,CACNG6,CACNG8,CALM1,CALM3,CAMK2B,CAMK2D,CAMK2G,CREB1,CREB3,CREB3L2,CREB3L3,CREB5,CREM,GNAI3,GNAQ,GNAS,KCNE1,MAPK1,MAPK11,MAPK13,MAPK14,MYL3,PIK3CA,PIK3CG,PIK3R1,PIK3R3,PIK3R5,PLCB3,PLN,PPP1CA,PPP1CC,PPP1R1A,PPP2CA,PPP2R1A,PPP2R1B,PPP2R2B,PPP2R2C,PPP2R5A,PPP2R5C,PRKACA,PRKCA,RAPGEF3,SCN1B,SCN4B,SLC8A1,SLC9A1,TNNT2,TPM1,TPM3
Apoptosis	experimental (any)	0.027	AKT1, CYCS,PRKACA,TNFRSF10D
Bladder cancer	experimental (any)	0.027	CDKN1A, E2F2,ERBB2
Cocaine addiction	experimental (any)	0.027	BDNF, MAOA,PRKACA
Measles	experimental (any)	0.027	AKT1, CDK6,CSNK2A1,HSPA1B,TNFRSF10D
Progesterone-mediated oocyte maturation	experimental (any)	0.027	AKT1, CCNA2,PLK1,PRKACA
Transcriptional misregulation in cancer	experimental (any)	0.027	CCNT2, CDKN1A,CSF1R,H3F3B,MAX,SP1
Vasopressin-regulated water reabsorption	experimental (any)	0.027	PRKACA, RAB5B,STX4
Neurotrophin signaling pathway	predicted (intersection)	0.029	AKT2,BDNF,CALM1,CALM3,CAMK2B,CAMK2D,CAMK2G,CAMK4,FOXO3,IRAK2,MAP2K2,MAP3K3,MAPK1,MAPK11,MAPK13,MAPK14,MAPKAPK2,NGFR,NRAS,NTRK2,NTRK3,PDPK1,PLCG1,PRDM4,PSEN1,PTPN11,RAPGEF1,RELA,RHOA,RPS6KA2,SHC1,SHC3,TP53,TP73,TRAF6
TGF-beta signaling pathway	predicted (intersection)	0.029	ACVR1B,ACVR2B,BAMBI,BMP2,BMP8A,BMPR1A,BMPR2,CHRD,EP300,GDF6,GDF7,INHBB,MAPK1,PPP2R1B,RBL1,RBX1,RHOA,RPS6KB2,SKP1,SMAD3,SMAD5,SMAD7,SMURF1,SP1,TGFBR1,ZFYVE16
FoxO signaling pathway	predicted (union)	0.032	AGAP2,AKT2,ARAF,BCL6,BNIP3,CCNB1,CCND1,CCNG2,CDKN1A,CREBBP,CSNK1E,EGF,EGFR,EP300,FASLG,FOXO1,FOXO3,GABARAPL1,GADD45G,GRM1,HOMER1,HOMER3,IGF1,INSR,IRS1,IRS2,IRS4,KLF2,KRAS,MAP2K1,MAP2K2,MAPK1,MAPK10,MAPK11,MAPK13,MAPK14,MAPK8,MAPK9,NLK,NRAS,PCK1,PDPK1,PIK3CA,PIK3CG,PIK3R1,PIK3R3,PIK3R5,PLK2,PRKAA2,PRKAB1,PRKAB2,PTEN,RBL2,S1PR1,SETD7,SIRT1,SMAD2,SMAD3,SOD2,STAT3,STK4,TGFBR1,TGFBR2,USP7
Rap1 signaling pathway	predicted (intersection)	0.033	ADCY1,ADCY2,AFDN,AKT2,ANGPT4,ARAP3,CALM1,CALM3,CDH1,CSF1R,DOCK4,EFNA5,EGF,EGFR,FGF2,FGFR1,FGFR2,FGFR3,GNAI3,GRIN2A,GRIN2B,INSR,ITGAL,ITGB3,LAT,MAGI1,MAGI2,MAGI3,MAP2K2,MAPK1,MAPK11,MAPK13,MAPK14,MRAS,NGFR,NRAS,P2RY1,PARD6G,PDGFC,PFN2,PLCE1,PLCG1,PRKCA,PRKCI,RAPGEF1,RAPGEF3,RGS14,RHOA,SIPA1,SRC,TIAM1,TLN2,VASP
mTOR signaling pathway	predicted (intersection)	0.033	AKT2,CAB39,CAB39L,DDIT4,EIF4E2,EIF4EBP1,MAPK1,MLST8,PDPK1,PRKAA2,PRKCA,PTEN,RPS6KA2,RPS6KB2,RPTOR,RRAGC,RRAGD,TSC1,ULK2,ULK3
Thyroid cancer	predicted (intersection)	0.034	CDH1, LEF1,MAP2K2,MAPK1,NRAS,PAX8,RET,RXRA,TCF7,TCF7L2,TP53,TPM3
Prolactin signaling pathway	predicted (intersection)	0.035	AKT2,CISH,ELF5,ESR1,ESR2,FOS,FOXO3,GCK,MAP2K2,MAPK1,MAPK11,MAPK13,MAPK14,NRAS,RELA,SHC1,SHC3,SOCS2,SRC,STAT1,STAT5A,TNFRSF11A
Wnt signaling pathway	predicted (intersection)	0.036	BAMBI,BTRC,CAMK2B,CAMK2D,CAMK2G,CSNK1A1,CSNK1E,DVL3,EP300,FOSL1,FRAT2,FZD5,FZD6,FZD8,LEF1,MAP3K7,NFATC2,NFATC3,NFATC4,NKD1,PRKCA,PSEN1,RBX1,RHOA,ROCK2,SFRP2,SKP1,SMAD3,TCF7,TCF7L2,TP53,VANGL2,WNT1,WNT3,WNT4,WNT8B,WNT9A,WNT9B
Ribosome	experimental (any)	0.037	RPL24, RPL35A,RPS2,RPS4X,RPSA
Pathways in cancer	predicted (union)	0.040	ABL1,AKT2,APPL1,AR,ARAF,ARNT2,BCL2,BCL2L1,BCR,BIRC5,BMP2,CASP9,CBL,CBLB,CCND1,CDC42,CDH1,CDK6,CDKN1A,CEBPA,COL4A1,COL4A4,COL4A6,CREBBP,CRK,CSF1R,CTBP1,CYCS,DCC,DVL3,E2F2,EGF,EGFR,ELOB,EP300,EPAS1,FAS,FASLG,FGF10,FGF12,FGF2,FGF23,FGFR1,FGFR2,FGFR3,FLT3,FOS,FOXO1,FZD3,FZD4,FZD5,FZD6,FZD8,FZD9,GLI2,GLI3,GSK3B,HDAC1,HDAC2,HHIP,HSP90B1,IGF1,ITGA3,KRAS,LAMA4,LAMA5,LAMB3,LAMC1,LAMC3,LEF1,MAP2K1,MAP2K2,MAPK1,MAPK10,MAPK8,MAPK9,MAX,MECOM,MET,MITF,MLH1,MMP1,MSH2,MSH3,MSH6,NFKBIA,NRAS,PAX8,PDGFB,PDGFRB,PIAS2,PIK3CA,PIK3CG,PIK3R1,PIK3R3,PIK3R5,PLCG1,PLD1,PML,PPARG,PRKCA,PTCH1,PTEN,RAC1,RAD51,RALBP1,RBX1,RELA,RET,RHOA,RUNX1,RXRA,SHH,SLC2A1,SMAD2,SMAD3,STAT1,STAT3,STAT5A,STK4,SUFU,TCF7,TCF7L1,TCF7L2,TFG,TGFBR1,TGFBR2,TP53,TPM3,TPR,TRAF1,TRAF2,TRAF3,TRAF6,WNT1,WNT11,WNT2B,WNT3,WNT3A,WNT4,WNT7B,WNT8A,WNT8B,WNT9A,WNT9B,XIAP,ZBTB16

	HTLV-I infection	experimental (any)	0.041	AKT1, BUB1B,CDKN1A,E2F2,PRKACA,SLC2A1,WNT1
	PI3K-Akt signaling pathway	experimental (any)	0.041	AKT1, CDK6,CDKN1A,CSF1R,DDIT4,GRB2,PTEN,YWHAZ
	Wnt signaling pathway	experimental (any)	0.041	CSNK2A1, FRAT2,PRKACA,TCF7,WNT1
	Amphetamine addiction	predicted (union)	0.042	ADCY5,ARC,ATF2,CACNA1C,CALM1,CALM3,CAMK2B,CAMK2D,CAMK2G,CAMK4,CREB1,CREB3,CREB3L2,CREB3L3,CREB5,FOS,GNAS,GRIA1,GRIN1,GRIN2A,GRIN2B,GRIN2D,GRIN3A,HDAC1,MAOA,PDYN,PPP1CA,PPP1CC,PPP3CB,PPP3R1,PPP3R2,PRKACA,PRKCA,SIRT1,SLC18A2,SLC6A3,STX1A
	Amyotrophic lateral sclerosis (ALS)	predicted (union)	0.042	ALS2,BCL2,BCL2L1,CASP9,CCS,CYCS,DERL1,GRIA1,GRIN1,GRIN2A,GRIN2B,GRIN2D,MAP2K6,MAP3K5,MAPK11,MAPK13,MAPK14,NEFM,NOS1,PPP3CB,PPP3R1,PPP3R2,RAC1,SLC1A2,TNFRSF1A,TNFRSF1B,TOMM40,TOMM40L,TP53
	Long-term depression	predicted (union)	0.042	ARAF,CRHR1,GNA11,GNA12,GNAI3,GNAO1,GNAQ,GNAS,GRIA1,GRM1,GUCY1A1,GUCY1A2,GUCY1B1,IGF1,JMJD7-PLA2G4B,KRAS,LYN,MAP2K1,MAP2K2,MAPK1,NOS1,NRAS,PLA2G4A,PLA2G4F,PLCB3,PPP2CA,PPP2R1A,PPP2R1B,PRKCA,PRKG1,PRKG2,RYR1
	TGF-beta signaling pathway	predicted (union)	0.042	ACVR1B,ACVR1C,ACVR2B,BAMBI,BMP2,BMP7,BMP8A,BMPR1A,BMPR1B,BMPR2,CHRD,CREBBP,CUL1,EP300,GDF6,GDF7,ID1,ID4,INHBB,INHBE,LEFTY2,MAPK1,PPP2CA,PPP2R1A,PPP2R1B,RBL1,RBX1,RHOA,ROCK1,RPS6KB2,SKP1,SMAD1,SMAD2,SMAD3,SMAD5,SMAD6,SMAD7,SMURF1,SP1,TFDP1,TGFBR1,TGFBR2,ZFYVE16

Supplementary Table S4. Pathway analysis using EVmiRNAs downstream of up and down regulated miRNAs in nicotine-treated DPSCs vs control.

**Upregulated miRNAs were highlighted in red**				**ND: No data**
**Downregulated miRNAs were highlighted in blue**				
**http://bioinfo.life.hust.edu.cn/EVmiRNA#!/**				
**miRNA**	**KEGG**	**KEGG_dscp**	**Gene**	**P-value**
hsa-miR-4497	No know pathways			
hsa-miR-7977	kegg	kegg_dscp	gene	pvalue
	ko05218	Melanoma	IGF1R,IGF1,TP53,CDKN1A,ARAF,MDM2,KRAS,FGFR1	5.54E-55
	ko05216	Thyroid cancer	TP53,KRAS,TPM3,NTRK1,PAX8	1.20E-45
	ko05214	Glioma	IGF1R,IGF1,TP53,CDKN1A,ARAF,MDM2,KRAS	2.44E-60
	ko03450	Non-homologous end-joining	POLL,XRCC5,DCLRE1C,NHEJ1	3.59E-32
	ko05340	Primary immunodeficiency	IL7R,AIRE,CIITA,TNFRSF13C,CD4,CD3D,DCLRE1C	1.75E-52
	ko00531	Glycosaminoglycan degradation	HPSE,GNS,ARSB,SGSH	1.36E-22
	ko04115	p53 signaling pathway	IGF1,TP53,CDKN1A,CASP8,IGFBP3,CDK2,PPM1D,TP73,DDB2,MDM2,MDM4,APAF1	7.54E-76
	ko05014	Amyotrophic lateral sclerosis (ALS)	NOS1,TNFRSF1B,GRIN2B,CASP12,SLC1A2,TP53,APAF1,BCL2	1.12E-62
	ko05160	Hepatitis C	RIPK1,STAT3,STAT2,CDKN1A,EIF2AK2,SCARB1,IFNAR2,ARAF,OCLN,TP53,MAVS,RNASEL,KRAS	5.15E-99
hsa-miR-3178	No know pathways			
hsa-miR-1260b	ko05215	Prostate cancer	ERBB2,TCF7,GRB2,CDKN1B,CDKN1A,GSK3B,CTNNB1,KLK3,BCL2,PDPK1,MDM2,EP300,FGFR1	4.82E-82
	ko05213	Endometrial cancer	ERBB2,TCF7,GRB2,GSK3B,CTNNB1,ELK1,PDPK1	1.13E-55
	ko05220	Chronic myeloid leukemia	TGFBR1,SMAD4,CDKN1B,CDKN1A,GAB2,GRB2,CRK,MDM2,PTPN11	6.76E-71
	ko04520	Adherens junction	ERBB2,SRC,TGFBR1,SMAD4,TCF7,CTNNB1,PTPRF,CTNND1,PTPN1,EP300,PTPRJ,FGFR1	1.10E-72
hsa-miR-10400-5p	No know pathways			
hsa-let-7e-5p	ko04064	NF-kappa B signaling pathway	BCL2L1,UBE2I,PTGS2,ERC1,XIAP,TNFRSF1A,MAP3K7,TNFAIP3,PLCG2,PLCG1,VCAM1,TLR4,TRIM25,LYN	6.05E-121
	ko03040	Spliceosome	NCBP1,SF3B4,SF3B2,PRPF8,DHX15,RBMX,SNRNP200,RBM8A,PRPF31,HNRNPU,EFTUD2,HNRNPM,SF3A2,SF3A3,HNRNPC,CDC5L,WBP11,SNRPC,SNRPA,CWC15,PRPF38B,DDX46,EIF4A3	2.64E-151
	ko04664	Fc epsilon RI signaling pathway	RAC1,NRAS,IL13,GAB2,PLCG2,PLCG1,MAP2K7,MAP2K4,LYN	1.20E-69
	ko04666	Fc gamma R-mediated phagocytosis	MARCKSL1,RAC1,LIMK2,GAB2,ARF6,ARPC4,PLCG2,PLCG1,MARCKS,CRK,LYN,PAK1	4.82E-81
	ko05320	Autoimmune thyroid disease	IL10,CD80,CD28,PRF1,CD86	6.82E-45
	ko04380	Osteoclast differentiation	TGFBR2,ITGB3,SOCS1,STAT2,SOCS3,TNFRSF1A,MAP2K7,RAC1,GAB2,MAP3K7,CREB1,PLCG2,FOSL1,IL1A,TYK2,TGFBR1	1.31E-133
	ko05219	Bladder cancer	TP53,CCND1,CDKN1A,NRAS,RB1,CDK4,MMP9,MYC,MDM2	4.33E-50
	ko05218	Melanoma	IGF1R,IGF1,TP53,CCND1,CDKN1A,NRAS,RB1,PTEN,CDK4,CDK6,MDM2,FGFR1	3.65E-52
	ko05217	Basal cell carcinoma	FZD2,FZD3,TP53,WNT1,TCF7L1,TCF7L2,FZD4	1.45E-67
	ko05216	Thyroid cancer	TP53,CCND1,TFG,NRAS,TCF7L1,TCF7L2,MYC	1.37E-45
	ko05215	Prostate cancer	IGF1R,IGF1,TP53,CREB1,CCND1,CDKN1A,NRAS,CDK2,RB1,TCF7L1,PTEN,FOXO1,TCF7L2,MDM2,FGFR1	6.74E-95
	ko05214	Glioma	IGF1R,IGF1,TP53,PTEN,CCND1,CDKN1A,NRAS,RB1,PLCG2,PLCG1,CDK4,CDK6,MDM2	1.88E-53
	ko05213	Endometrial cancer	TP53,CCND1,NRAS,TCF7L1,TCF7L2,PTEN,MLH1,MYC	2.46E-63
	ko05212	Pancreatic cancer	BCL2L1,TGFBR2,TGFBR1,STAT3,RAC1,CCND1,RB1,CDK4,CDK6,TP53	2.14E-82
	ko05211	Renal cell carcinoma	FLCN,RAC1,NRAS,RAP1A,ARNT2,CRK,PAK1,CUL2,PAK3	1.82E-81
	ko05210	Colorectal cancer	TGFBR2,TGFBR1,TP53,CASP3,RAC1,CCND1,TCF7L1,MSH6,MLH1,TCF7L2,MYC	1.95E-73
	ko00601	Glycosphingolipid biosynthesis - lacto and neolacto series	B4GALT1,B3GNT5,B4GALT3,B3GNT1,GCNT2	6.82E-45
	ko04672	Intestinal immune network for IgA production	ICOS,CD28,IL10,CD80,CCR10,CD86,ICOSLG	3.10E-62
	ko05330	Allograft rejection	IL10,CD80,CD28,PRF1,CD86	6.17E-39
	ko05332	Graft-versus-host disease	CD80,CD28,IL1A,PRF1,CD86	1.01E-30
	ko05200	Pathways in cancer	BCL2L1,PTGS2,PTK2,RB1,CDKN1A,ITGA3,TGFBR1,MSH6,PLCG2,XIAP,RUNX1T1,ITGAV,SKP2,CRK,FZD2,FZD3,STAT3,NRAS,MLH1,CBL,MYC,CUL2,TGFBR2,IGF1R,IGF1,PLCG1,RAC1,MMP9,FOXO1,STK4,LAMC3,MDM2,LAMC1,ARNT2,FGFR1,TP53,CASP3,FN1,CCND1,WNT1,TCF7L1,TCF7L2,PTEN,CDK2,CDK4,CDK6,FZD4,TFG	3.84E-245
	ko05202	Transcriptional misregulation in cancers	BCL2L1,PTK2,CDKN1A,FOXO1,RUNX1T1,NCOR1,SPINT1,BMP2K,CD86,JMJD1C,MYC,SIN3A,TGFBR2,IGF1R,IGF1,NUPR1,HMGA2,MDM2,PBX3,ARNT2,PBX1,TP53,CCND2,ELK4,MMP9	8.67E-179
	ko05203	Viral carcinogenesis	PMAIP1,CDKN1A,SRF,STAT3,CHD4,NRAS,RB1,LYN,YWHAE,DDX3X,RBL2,RAC1,CREB1,SKP2,MDM2,TP53,CASP3,CCND1,CCND2,CDK2,CDK4,CDK6,ATF6B	6.53E-168
	ko05416	Viral myocarditis	CD28,CASP3,RAC1,CCND1,CD80,CD86,PRF1,ABL2	1.47E-61
	ko04310	Wnt signaling pathway	FZD2,FZD3,TP53,NLK,CCND1,CHD8,PSEN1,CCND2,TCF7L1,TCF7L2,MAP3K7,FOSL1,WNT1,RAC1,FZD4,MYC,GPC4,CUL1	9.90E-131
	ko04940	Type I diabetes mellitus	IL1A,CD28,CD86,PRF1,CD80	6.33E-43
	ko00533	Glycosaminoglycan biosynthesis - keratan sulfate	B4GALT1,B4GALT3,B3GNT1	1.47E-27
	ko04120	Ubiquitin mediated proteolysis	UBE2I,UBE4B,ERCC8,UBOX5,SOCS1,UBE2J1,SOCS3,UBE2H,UBE3B,TRIM32,MAP3K1,CBL,HERC1,NEDD4L,XIAP,PIAS1,BIRC6,SKP2,MDM2,CUL1,CUL2	1.57E-160
	ko04510	Focal adhesion	PTK2,ITGA3,ITGB3,RAP1A,XIAP,CRK,PPP1R12A,IGF1R,IGF1,DIAPH1,RAC1,ITGB8,VCL,LAMC3,LAMC1,FN1,CCND1,CCND2,ITGAV,PTEN,FLNA,PAK1,PAK3	3.63E-169

	ko04012	ErbB signaling pathway	PTK2,EIF4EBP1,CDKN1A,NRAS,CBL,PLCG2,PLCG1,MAP2K7,MAP2K4,CRK,MYC,ABL2,PAK1,PAK3	3.50E-86
	ko04010	MAPK signaling pathway	MAP4K3,RASGRP1,MAP2K4,RAP1A,MAP2K7,RAPGEF2,IL1A,CRK,MAP3K2,MAP3K1,NRAS,MAP3K7,CACNB4,NF1,MYC,TGFBR2,TGFBR1,SRF,RAC1,CDC25B,NR4A1,STK4,NLK,FGFR1,TP53,CASP3,TNFRSF1A,ELK4,FLNA,PAK1	2.33E-211
	ko04151	PI3K-Akt signaling pathway	BCL2L1,PTK2,CDKN1A,ITGA3,ITGB3,INSR,CRTC2,TSC1,PTEN,PRLR,NRAS,OSMR,CDK4,MYC,YWHAE,IGF1R,IGF1,CDK6,RBL2,RAC1,ITGB8,CREB1,IL6R,LAMC3,MDM2,LAMC1,FGFR1,TP53,FN1,CCND1,CCND2,ITGAV,F2R,CDK2,GNG5,EIF4EBP1,TLR4,ATF6B,LPAR1	1.72E-243
	ko00563	Glycosylphosphatidylinositol(GPI)-anchor biosynthesis	PIGA,PIGM,PIGN,PIGP,PIGS,PIGT,PIGW	9.80E-42
	ko04370	VEGF signaling pathway	PTGS2,PTK2,RAC1,NRAS,PLCG2,PLCG1	2.53E-59
	ko04110	Cell cycle	CDC25A,TP53,RBL2,CCND1,CDC25B,CDKN1A,CCND2,RB1,CUL1,TTK,MCM6,CDK2,WEE1,CDK4,CDK6,SKP2,MYC,MDM2,YWHAE	5.94E-141
	ko04115	p53 signaling pathway	IGF1,PMAIP1,CASP3,PTEN,CCND1,CDKN1A,CCND2,CCNG1,CDK2,CDK4,RRM2,CDK6,TP53,MDM2,MDM4,STEAP3,APAF1	6.61E-75
	ko03013	RNA transport	NCBP1,EIF1,EIF5B,EIF2S3,EIF2S2,XPO1,EIF3J,XPO5,RBM8A,EIF3D,NDC1,NMD3,NUP214,RANBP2,XPOT,NUP98,NUP160,RANGAP1,RAE1,NUP62,THOC5,UBE2I,TACC3,GEMIN4,EIF4EBP1,NUP205,NUP155,EIF4A3,NUPL1	2.08E-157
	ko05222	Small cell lung cancer	BCL2L1,PTGS2,PTK2,SKP2,TP53,FN1,CCND1,ITGA3,CDK2,RB1,PTEN,XIAP,CDK4,ITGAV,CDK6,LAMC3,MYC,LAMC1,APAF1	9.56E-82
	ko05223	Non-small cell lung cancer	TP53,CCND1,NRAS,RB1,PLCG2,PLCG1,STK4,CDK6,CDK4	1.92E-64
	ko05220	Chronic myeloid leukemia	BCL2L1,TGFBR2,TGFBR1,TP53,CCND1,GAB2,CDKN1A,NRAS,CBL,CDK4,RB1,CDK6,CRK,MDM2,MYC	4.19E-71
	ko05221	Acute myeloid leukemia	STAT3,CCND1,NRAS,TCF7L1,TCF7L2,EIF4EBP1,RUNX1T1,MYC	8.90E-74
	ko05014	Amyotrophic lateral sclerosis (ALS)	BCL2L1,NOS1,TNFRSF1B,CASP3,RAC1,TNFRSF1A,DERL1,TP53,APAF1	6.37E-68
	ko04520	Adherens junction	TGFBR2,IGF1R,SSX2IP,RAC1,PTPRJ,VCL,TGFBR1,TCF7L2,MAP3K7,INSR,PVRL4,PTPN1,TCF7L1,NLK,FGFR1	8.23E-81
	ko05164	Influenza A	XPO1,FDPS,TNFSF10,STAT2,SOCS3,TNFRSF1A,TRIM25,ADAR,NUP98,TBK1,MAP2K7,RAE1,TLR4,MAP2K4,IL1A,TYK2,MAVS,CPSF4,HNRNPUL1	5.47E-149
	ko05166	HTLV-I infection	BCL2L1,MAP3K1,CDKN1A,XIAP,CRTC2,MAP2K4,XPO1,FZD2,FZD3,FZD4,EGR1,NRAS,RB1,POLD1,FOSL1,MYC,TGFBR2,TGFBR1,SRF,CREB1,TP53INP1,TRRAP,NRP1,FDPS,TP53,TNFRSF1A,CCND1,WNT1,CCND2,ADCY9,CDK4,ELK4,VCAM1	6.97E-228
	ko05161	Hepatitis B	CDKN1A,HSPG2,MAP2K4,STAT3,STAT2,TBK1,MAP3K1,NRAS,RB1,MYC,APAF1,TGFBR1,DDX3X,TLR4,CREB1,TP53,CASP3,CCND1,PTEN,CDK2,CDK4,CDK6,MMP9,ATF6B,MAVS	3.86E-130
	ko05162	Measles	CD28,STAT3,STAT2,TBK1,CCND1,ADAR,CCND2,IL13,MAP3K7,TNFAIP3,MSN,CDK2,TNFSF10,CDK4,TP53,TLR4,CDK6,IL1A,TYK2,MAVS,CD46	2.19E-131
	ko05169	Epstein-Barr virus infection	CDKN1A,PSMD2,PLCG2,PLCG1,MAP2K7,MAP2K4,TYK2,PSMC2,XPO1,STAT3,TBK1,MAP3K7,RB1,SPN,NUP214,LYN,YWHAE,HSPB2,SKP2,MDM2,MYC,TP53,IL10,TNFAIP3,VIM,CDK2	6.86E-183
**hsa-let-7a-5p**	ko04062	Chemokine signaling pathway	CCL3,PTK2,HRAS,CCR5,CCR7,CRK,STAT3,STAT2,PREX1,NRAS,NFKB1,LYN,SRC,RAC1,CCR10,GNB1,KRAS,PARD3,ADCY9,GNG5,BRAF,PAK1	1.67E-180
	ko04064	NF-kappa B signaling pathway	BCL2L1,UBE2I,TLR4,PTGS2,ERC1,MAP3K7,TNFAIP3,PLCG2,PLCG1,VCAM1,PIAS4,TRIM25,NFKB1,LYN,BCL2,TNFRSF11A	1.80E-121
	ko04144	Endocytosis	EHD4,EHD1,RAB4A,HRAS,STAMBP,EGFR,TGFBR1,CCR5,CAV1,AP2S1,ADRB1,EEA1,ARF6,RAB11A,TFRC,RABEP1,TGFBR2,IGF1R,NEDD4L,RNF41,PARD3,MDM2,ZFYVE20,VPS36,SRC,DNAJC6,VPS25,CBL,F2R,ADRB2,ADRB3,GIT2	1.32E-165
	ko04141	Protein processing in endoplasmic reticulum	UBE2J1,SEC63,PRKCSH,MAP2K7,FBXO6,NPLOC4,HERPUD1,SYVN1,DNAJB11,UBE2G2,CUL1,ERN1,OS9,EDEM2,EDEM3,DNAJA2,UBE4B,ERO1L,MBTPS2,MBTPS1,EIF2AK1,CALR,EIF2AK4,PREB,ATF4,ATF6B,CKAP4,BCL2	8.74E-175
	ko03040	Spliceosome	NCBP1,SF3B5,SF3B4,SF3B1,SF3B2,PRPF8,THOC2,SNRNP200,PRPF31,HNRNPU,EFTUD2,SF3A1,SF3A2,HNRNPM,TCERG1,WBP11,CHERP,SNRPC,SNRPA,PRPF38B,RBMX,EIF4A3	2.46E-160
	ko00512	Mucin type O-glycan biosynthesis	C1GALT1,B4GALT5	2.39E-24
	ko04664	Fc epsilon RI signaling pathway	IL13,RAC1,NRAS,HRAS,FYN,GAB2,PLCG2,PLCG1,MAP2K7,MAP2K4,LYN,KRAS	5.55E-65
	ko04666	Fc gamma R-mediated phagocytosis	MARCKSL1,WASF2,RAC1,LIMK2,ARPC2,GAB2,ARF6,PLCG2,PLCG1,MARCKS,CRK,LYN,PAK1,GSN	1.57E-79
	ko04662	B cell receptor signaling pathway	FOS,CD81,HRAS,RAC1,NRAS,PLCG2,NFKB1,LYN,PTPN6,KRAS	8.67E-84
	ko05320	Autoimmune thyroid disease	IL10,CD80,CD28,PRF1,CD86	2.40E-46
	ko04380	Osteoclast differentiation	TGFBR2,ITGB3,SOCS1,STAT2,SOCS3,FOS,FYN,MAP2K7,RAC1,GAB2,MAP3K7,PLCG2,FOSL1,TNFRSF11A,JAK1,NFKB1,TYK2,TGFBR1	6.05E-135
	ko04360	Axon guidance	FYN,PTK2,RAC1,EPHA7,NRAS,HRAS,EPHA4,EPHA3,EPHA2,PAK1,LIMK2,DPYSL5,RGS3,ABL1,PAK3,NRP1,KRAS	3.86E-113
	ko05219	Bladder cancer	TP53,E2F2,CCND1,EGFR,HRAS,NRAS,RB1,MDM2,BRAF,CDK4,MYC,CDKN1A,KRAS	2.59E-42
	ko05218	Melanoma	IGF1R,IGF1,TP53,E2F2,CCND1,EGFR,HRAS,NRAS,RB1,CDK4,BRAF,CDK6,MDM2,KRAS,CDKN1A,FGFR1	6.17E-45
	ko05216	Thyroid cancer	TP53,CCND1,HRAS,NRAS,TCF7L2,TPR,MYC,BRAF,KRAS	4.28E-42
	ko05215	Prostate cancer	CDKN1A,EGFR,FOXO1,NRAS,RB1,NFKB1,FGFR1,HRAS,IGF1R,IGF1,MDM2,EP300,KRAS,TP53,E2F2,CCND1,TCF7L2,CASP9,CDK2,BRAF,ATF4,BCL2	1.47E-83
	ko05214	Glioma	IGF1R,IGF1,TP53,E2F2,CCND1,EGFR,HRAS,NRAS,RB1,PLCG2,PLCG1,CDK4,BRAF,CDK6,MDM2,CDKN1A,KRAS	2.06E-46
	ko05213	Endometrial cancer	TP53,AXIN1,CCND1,EGFR,HRAS,NRAS,TCF7L2,CASP9,MLH1,MYC,BRAF,KRAS	6.72E-56
	ko05212	Pancreatic cancer	BCL2L1,TGFBR2,TGFBR1,STAT3,TP53,RAC1,CCND1,E2F2,EGFR,RB1,CASP9,CDK4,BRAF,CDK6,JAK1,NFKB1,KRAS	2.08E-69
	ko05211	Renal cell carcinoma	FLCN,RAC1,HRAS,NRAS,BRAF,SLC2A1,ARNT2,CRK,KRAS,EP300,PAK1,CUL2,PAK3	5.66E-75

ko05210	Colorectal cancer	TGFBR2,TGFBR1,TP53,CASP3,AXIN1,RAC1,CCND1,FOS,TCF7L2,MLH1,CASP9,MSH6,BCL2,MYC,BRAF,KRAS	1.05E-64
ko05131	Shigellosis	SRC,WASF2,RAC1,ARPC2,VCL,RIPK2,NOD1,NFKB1,ABL1,CRK,RHOG	1.44E-96
ko03410	Base excision repair	PCNA,SMUG1,MBD4,POLD3,UNG,XRCC1,POLL	8.74E-72
ko04630	Jak-STAT signaling pathway	BCL2L1,IL13,LIFR,TYK2,STAT3,STAT2,PRLR,IL12RB2,OSMR,JAK1,MYC,IL6R,EP300,PTPN6,SOCS5,SOCS4,SOCS1,SOCS3,CCND1,CCND2,CBL,IL6,IL10,PIAS1,PIAS4	1.71E-155
ko04730	Long-term depression	NOS1,IGF1R,IGF1,HRAS,NRAS,BRAF,ITPR2,ITPR3,LYN,KRAS	3.87E-66
ko04810	Regulation of actin cytoskeleton	PTK2,LIMK2,HRAS,ITGA3,EGFR,MSN,CRK,GSN,SLC9A1,WASF2,ARHGEF7,NRAS,PPP1R12A,KRAS,SRC,ITGB3,DIAPH2,RAC1,ARPC2,VCL,RDX,FGFR1,BDKRB2,FN1,ITGAV,F2R,BRAF,PAK1,PAK3	5.45E-178
ko04210	Apoptosis	BCL2L1,CASP6,TNFSF10,TP53,CASP3,BID,CASP8,CASP9,FADD,IRAK2,NFKB1,APAF1,BCL2	8.31E-97
ko05100	Bacterial invasion of epithelial cells	SRC,PTK2,FN1,WASF2,RAC1,ARPC2,VCL,CBL,CD2AP,CRK,RHOG,CAV1	9.12E-101
ko00601	Glycosphingolipid biosynthesis - lacto and neolacto series	B4GALT1,B3GNT5,B4GALT3,B3GNT1,GCNT2	2.40E-46
ko04660	T cell receptor signaling pathway	ICOS,RASGRP1,FOS,IL10,NRAS,HRAS,FYN,MAP3K7,CBL,PAK1,PLCG1,CD28,MAP2K7,NFKB1,PAK3,CDK4,PTPN6,KRAS	1.74E-112
ko04672	Intestinal immune network for IgA production	ICOS,CD28,IL10,CD80,CCR10,CD86,IL6,ICOSLG	7.12E-62
ko05330	Allograft rejection	IL10,CD80,CD28,PRF1,CD86	3.58E-40
ko04912	GnRH signaling pathway	MMP14,SRC,MAP3K2,MAP3K3,HRAS,MAP3K1,EGFR,ADCY9,ATF4,MAP2K7,MAP2K4,ITPR2,ITPR3,NRAS,KRAS	1.17E-85
ko05332	Graft-versus-host disease	IL6,CD80,CD28,PRF1,CD86	1.15E-31
ko04910	Insulin signaling pathway	SOCS4,SOCS1,EIF4EBP1,SOCS3,HRAS,NRAS,FOXO1,CBL,INSR,FASN,BRAF,CRK,KRAS,TSC1	8.94E-117
ko04350	TGF-beta signaling pathway	TGFBR2,TGFBR1,SP1,ACVR2B,BMPR1B,CHRD,BMPR2,MYC,EP300,CUL1	2.50E-80
ko05200	Pathways in cancer	BCL2L1,PTGS2,PTK2,RB1,HRAS,ITGA3,EGFR,TGFBR1,MSH6,PLCG2,FOXO1,RUNX1T1,SLC2A1,SKP2,ABL1,CRK,FZD2,FZD3,STAT3,AXIN1,BID,RAC1,NRAS,MLH1,CBL,JUP,JAK1,NFKB1,MYC,KRAS,CDKN1A,CUL2,TGFBR2,IGF1R,IGF1,CASP3,FOS,FADD,CASP8,STK4,TPR,LAMC3,MDM2,LAMC1,ARNT2,FGFR1,EP300,TP53,E2F2,FN1,CCND1,MAX,PLCG1,TCF7L2,CASP9,IL6,ITGAV,CDK2,CDK4,BRAF,CDK6,FZD4,BCL2	4.37E-233
ko05202	Transcriptional misregulation in cancers	BCL2L1,PTK2,CDKN1A,MEN1,FOXO1,RUNX1T1,CCR7,NCOR1,SPINT1,MEIS1,CD86,JUP,JMJD1C,NFKB1,MYC,DOT1L,SIN3A,TGFBR2,IGF1R,IGF1,LYL1,SP1,TMPRSS2,HMGA2,MDM2,PBX3,ARNT2,PBX1,TP53,MAX,CCND2,IL6,ELK4,CDK9,EWSR1	1.24E-167
ko05203	Viral carcinogenesis	HDAC3,PMAIP1,HRAS,CCR5,SRF,GSN,STAT3,EGR3,NRAS,RB1,JAK1,NFKB1,LYN,DDB1,CDKN1A,YWHAE,SRC,DDX3X,RBL2,RAC1,SKP2,MDM2,EP300,KRAS,IRF3,TP53,CASP3,CCND1,CCND2,CASP8,CDK2,CDK4,CDK6,ATF4,ATF6B	5.95E-152
ko05142	Chagas disease (American trypanosomiasis)	CCL3,TGFBR1,BDKRB2,TGFBR2,IL10,FADD,CALR,FOS,CASP8,IL6,TLR4,MAP2K4,GNAL,NFKB1	6.21E-123
ko05145	Toxoplasmosis	BCL2L1,SOCS1,CASP3,STAT3,IL10,ALOX5,MAP3K7,CASP8,CASP9,NFKB1,CCR5,TLR4,JAK1,LAMC3,TYK2,LAMC1,BCL2,PPIF	5.20E-132
ko05416	Viral myocarditis	CD28,CASP3,RAC1,CCND1,CD80,BID,FYN,CASP8,CD86,DAG1,CASP9,ABL1,ABL2,PRF1,CAV1	3.59E-47
ko04725	Cholinergic synapse	KCNJ2,FOS,GNB1,HRAS,NRAS,FYN,ADCY9,GNG5,ATF4,ITPR2,ITPR3,CHRNA7,BCL2,KCNQ4,KRAS	9.69E-127
ko04726	Serotonergic synapse	PTGS2,PTGS1,CASP3,SLC6A4,NRAS,ALOX5,GNB1,GNG5,BRAF,ITPR2,ITPR3,APP,HRAS,KRAS	3.19E-115
ko04720	Long-term potentiation	EP300,HRAS,NRAS,BRAF,ATF4,ITPR2,ITPR3,KRAS,PPP1R12A	9.21E-67
ko04722	Neurotrophin signaling pathway	ATF4,TP53,BCL2,PLCG2,MAP3K3,HRAS,MAP3K1,NRAS,FRS2,PRDM4,ABL1,PLCG1,RIPK2,MAP2K7,BRAF,RAC1,NFKB1,IRAK2,CRK,YWHAE,KRAS	1.17E-113
ko04540	Gap junction	SRC,TJP1,MAP3K2,NRAS,HRAS,EGFR,ADCY9,TUBB,ADRB1,ITPR2,ITPR3,LPAR1,KRAS	4.00E-80
ko00533	Glycosaminoglycan biosynthesis - keratan sulfate	B4GALT1,B4GALT3,B3GNT1	2.33E-28
ko04530	Tight junction	SRC,ASH1L,TJP1,EXOC4,JAM3,HRAS,NRAS,VAPA,EPB41,PARD3,CDK4,RAB13,MPDZ,KRAS	1.35E-102
ko04120	Ubiquitin mediated proteolysis	WWP2,UBE2J1,TRIM32,HUWE1,UBOX5,SKP2,UBE3B,UBE3C,MAP3K1,SYVN1,UBE2G2,BIRC6,DDB1,CUL1,CUL2,HERC1,MDM2,UBE2I,UBE4B,SOCS1,SOCS3,CBL,NEDD4L,PIAS1,PIAS4,UBE2W	1.54E-157
ko04510	Focal adhesion	PTK2,HRAS,ITGA3,EGFR,ITGB3,CRK,CAV1,FYN,PPP1R12A,SRC,IGF1R,IGF1,RAC1,VCL,LAMC3,LAMC1,FN1,CCND1,CCND2,ITGAV,BRAF,FLNA,BCL2,PAK1,PAK3	5.78E-173
ko04012	ErbB signaling pathway	SRC,PTK2,EIF4EBP1,NRAS,HRAS,EGFR,CBL,PAK1,PAK3,PLCG2,PLCG1,MAP2K7,BRAF,MAP2K4,ABL1,CRK,MYC,ABL2,CDKN1A,KRAS	2.25E-76
ko04010	MAPK signaling pathway	MAP4K3,RASGRP1,HRAS,EGFR,RAC1,MAP2K7,RAPGEF2,MAP2K4,CRK,MAPKAPK5,DAXX,MAP3K2,MAP3K3,MAP3K1,NRAS,MAP3K7,CACNB4,NFKB1,MYC,FGFR1,TGFBR2,TGFBR1,SRF,FOS,CDC25B,NR4A1,STK4,ATF4,NLK,KRAS,TP53,CASP3,MAX,BRAF,ELK4,FLNA,PAK1	1.45E-208
ko04151	PI3K-Akt signaling pathway	BCL2L1,PTK2,HRAS,ITGA3,EGFR,EPHA2,ITGB3,INSR,TSC1,TEK,BCL2L11,PRLR,NRAS,OSMR,JAK1,NFKB1,MYC,KRAS,CDKN1A,YWHAE,IGF1R,IGF1,CDK6,RBL2,RAC1,GNB1,IL6R,ATF4,LAMC3,MDM2,LAMC1,FGFR1,TP53,FN1,CCND1,CCND2,ITGAV,CASP9,IL6,F2R,CDK2,GNG5,EIF4EBP1,TLR4,CDK4,ATF6B,LPAR1,BCL2	2.90E-240
ko03050	Proteasome	PSMD14,PSME4,PSMD11,PSMB3,PSMA2,PSMA4,PSMB7,PSMC4,PSMB2,PSMC6	7.78E-77
ko00563	Glycosylphosphatidylinositol(GPI)-anchor biosynthesis	PIGA,PIGM,PIGN,PIGP,PIGT,PIGW	1.04E-45
ko04650	Natural killer cell mediated cytotoxicity	TNFSF10,PAK1,CASP3,RAC1,NRAS,HRAS,FYN,BID,PLCG2,PLCG1,PRF1,BRAF,PTPN6,KRAS	2.54E-118

	ko00310	Lysine degradation	ASH1L,AASS,DLST,PLOD1,WHSC1L1,PLOD2,ALDH7A1,NSD1,DOT1L,SETD7	2.59E-97
	ko04970	Salivary secretion	NOS1,SLC9A1,ADRB3,ADCY9,ADRB1,ADRB2,KCNN4,SLC4A2,ITPR2,ITPR3	1.20E-95
	ko04370	VEGF signaling pathway	PTGS2,PTK2,SRC,HRAS,RAC1,NRAS,CASP9,PLCG2,PLCG1,KRAS	2.66E-51
	ko05120	Epithelial cell signaling in Helicobacter pylori infection	SRC,TJP1,CASP3,CSK,JAM3,RAC1,EGFR,ADAM10,PLCG2,PLCG1,NOD1,MAP2K4,NFKB1,LYN,PAK1	9.73E-133
	ko04110	Cell cycle	CDKN1A,PRKDC,ABL1,CDC7,SKP2,SMC3,RB1,WEE1,CUL1,YWHAE,PCNA,RBL2,CDC25B,CDC25A,MCM6,MCM3,MDM2,EP300,MYC,TP53,E2F2,CCND1,CCND2,CDK2,CDK4,CDK6	1.41E-132
	ko04115	p53 signaling pathway	IGF1,PMAIP1,CASP3,CCND1,CDKN1A,CCND2,STEAP3,CASP8,CASP9,CCNG1,CDK2,CDK4,RRM2,CDK6,TP53,MDM2,MDM4,BID,APAF1	1.59E-73
	ko04114	Oocyte meiosis	IGF1R,IGF1,STAG3,SMC3,SGOL1,ADCY9,CDK2,AURKA,SLK,ITPR2,ITPR3,CUL1,YWHAE	5.81E-116
	ko03013	RNA transport	NCBP1,EIF1,EIF2S3,EIF2S2,XPO1,EIF3J,NUP62,EIF3C,EIF3D,NDC1,NUP43,NMD3,NUP214,RANBP2,XPOT,NUPL1,NUP98,NUP160,TPR,THOC2,THOC5,UBE2I,TACC3,GEMIN4,NUP153,EIF4EBP1,NUP155,EIF4A3,RAE1	6.50E-165
	ko05222	Small cell lung cancer	BCL2L1,PTGS2,PTK2,ITGA3,SKP2,RB1,NFKB1,MYC,APAF1,LAMC3,LAMC1,TP53,E2F2,FN1,CCND1,MAX,ITGAV,CASP9,CDK2,CDK4,CDK6,BCL2	1.04E-78
	ko05223	Non-small cell lung cancer	TP53,E2F2,CCND1,NRAS,HRAS,EGFR,RB1,CASP9,PLCG2,PLCG1,STK4,BRAF,CDK6,CDK4,KRAS	1.11E-52
	ko05220	Chronic myeloid leukemia	BCL2L1,TGFBR2,TGFBR1,MYC,TP53,E2F2,CCND1,GAB2,HRAS,NRAS,RB1,CDK4,CBL,CDK6,NFKB1,ABL1,CRK,MDM2,BRAF,CDKN1A,KRAS	8.36E-61
	ko05221	Acute myeloid leukemia	STAT3,CCND1,HRAS,NRAS,TCF7L2,JUP,EIF4EBP1,BRAF,RUNX1T1,NFKB1,MYC,KRAS	9.06E-67
	ko04620	Toll-like receptor signaling pathway	IRF3,CCL3,TLR4,TBK1,FOS,CD80,FADD,MAP3K7,CASP8,CD86,IL6,MAP2K7,RAC1,MAP2K4,NFKB1	2.14E-111
	ko04622	RIG-I-like receptor signaling pathway	IRF3,DDX3X,TBK1,TRIM25,MAP3K1,MAP3K7,CASP8,ATG12,FADD,NFKB1,MAVS,TMEM173	9.63E-81
	ko05014	Amyotrophic lateral sclerosis (ALS)	BCL2L1,DAXX,TNFRSF1B,CASP3,NOS1,BID,RAC1,CASP9,TP53,APAF1,BCL2	1.51E-65
	ko04930	Type II diabetes mellitus	SOCS4,KCNJ11,SOCS1,SOCS3,INSR,ABCC8	1.58E-57
	ko04520	Adherens junction	TGFBR2,TGFBR1,TJP1,FARP2,WASF2,SRC,FYN,RAC1,EGFR,IGF1R,TCF7L2,MAP3K7,INSR,PARD3,NLK,PTPRB,PTPN6,EP300,SSX2IP,VCL,FGFR1	6.17E-71
	ko05164	Influenza A	TNFSF10,MAP2K7,MAP2K4,TYK2,XPO1,STAT2,TBK1,JAK1,NFKB1,IL18,TLR4,NUP98,TRIM25,TMPRSS2,RAE1,EP300,HNRNPUL1,IRF3,SOCS3,EIF2AK1,EIF2AK4,CASP9,IL6,KPNA2,KPNA1,MAVS	5.00E-141
	ko05166	HTLV-I infection	BCL2L1,EGR1,HRAS,MAP2K4,XPO1,FZD2,FZD3,FZD4,MAP3K3,MAP3K1,NRAS,RB1,POLD3,FOSL1,JAK1,NFKB1,MYC,CDKN1A,PCNA,TGFBR2,TGFBR1,SRF,FOS,TRRAP,TP53INP1,VCAM1,EP300,NRP1,KRAS,TP53,E2F2,CCND1,CALR,CCND2,ADCY9,SLC2A1,IL6,CDK4,ELK4,ATF4	1.70E-226
	ko05161	Hepatitis B	HRAS,HSPG2,CASP3,MAP2K4,STAT3,STAT2,TBK1,EGR3,MAP3K1,NRAS,RB1,FADD,NFKB1,MYC,DDB1,CDKN1A,APAF1,PCNA,SRC,TGFBR1,DDX3X,CDK6,FOS,JAK1,EP300,KRAS,IRF3,TP53,E2F2,CCND1,CASP8,CASP9,IL6,CDK2,CDK4,TLR4,ATF4,ATF6B,MAVS,BCL2	4.78E-107
	ko05160	Hepatitis C	IRF3,MAVS,STAT3,STAT2,TBK1,CD81,NRAS,HRAS,EGFR,SOCS3,EIF2AK4,EIF2AK1,PIAS1,TP53,JAK1,NFKB1,TYK2,BRAF,CDKN1A,KRAS	5.31E-102
	ko05162	Measles	CCND2,MSN,TNFSF10,TYK2,STAT3,STAT2,TBK1,FYN,MAP3K7,JAK1,NFKB1,CD28,CDK6,CD46,IRF3,TP53,CCND1,IL13,EIF2AK4,TNFAIP3,IL6,EIF2AK1,CDK2,CDK4,TLR4,MAVS	7.30E-127
	ko05169	Epstein-Barr virus infection	CDKN1A,PLCG2,PLCG1,MAP2K7,MAP2K4,TYK2,NCOR2,XPO1,STAT3,TBK1,MAP3K7,RB1,JAK1,NUP214,NFKB1,LYN,PSMC4,PSMC6,YWHAE,HSPB2,SKP2,MDM2,EP300,MYC,IRF3,TP53,EIF2AK1,PSMD14,PSMD11,EIF2AK4,TNFAIP3,IL10,CDK2,BCL2	5.92E-176
	ko05168	Herpes simplex infection	CD74,TYK2,MED8,DAXX,SKP2,STAT2,TBK1,MAP3K7,JAK1,NFKB1,CUL1,FOS,FADD,EP300,IRF3,TAF5,TP53,CASP3,SOCS3,EIF2AK1,EIF2AK4,CASP8,IL6,CDK2,TAF9B,MAVS	3.05E-160
**hsa-let-7d-5p**	ko04144	Endocytosis	TSG101,EHD4,TGFBR1,CLTA,CCR5,AP2B1,VPS25,EEA1,NEDD4,ARF6,RAB11A,RAB22A,CXCR4,TGFBR2,IGF1R,NEDD4L,RAB5C,RAB5B,LDLR,SNF8,MDM2,ZFYVE20,CBL,ADRB1,ADRB2,ADRB3	2.74E-164
	ko04141	Protein processing in endoplasmic reticulum	UBE2J1,MAP2K7,SVIP,TRAM1,HERPUD1,DNAJB1,DERL1,DNAJB11,CAPN1,ERP29,EDEM2,EDEM3,DNAJA2,UBE4B,ERO1L,DNAJA1,MBTPS2,MBTPS1,PPP1R15A,SSR1,ATF6B,CKAP4,BCL2	2.64E-171
	ko04664	Fc epsilon RI signaling pathway	IL13,RAC1,NRAS,FYN,GAB2,PLCG2,PLCG1,MAP2K7,MAP2K4,LYN,KRAS	2.03E-63
	ko04666	Fc gamma R-mediated phagocytosis	MARCKSL1,WASF1,WASF2,RAC1,LIMK2,GAB2,ARF6,PLCG2,PLCG1,MARCKS,LYN,PAK1	4.73E-79
	ko05320	Autoimmune thyroid disease	IL10,CD80,CD28,PRF1,CD86	5.82E-44
	ko04962	Vasopressin-regulated water reabsorption	VAMP2,RAB5C,RAB5B,ADCY9,NSF,RAB11A	3.12E-56
	ko05219	Bladder cancer	TP53,E2F2,CCND1,CDKN1A,NRAS,CDK4,BRAF,MYC,MDM2,KRAS	1.18E-46
	ko05218	Melanoma	IGF1R,IGF1,TP53,E2F2,CCND1,CDKN1A,NRAS,CDK4,BRAF,CDK6,MDM2,KRAS,FGFR1	9.50E-49
	ko05216	Thyroid cancer	TP53,CCND1,NRAS,BRAF,MYC,KRAS	4.37E-47
	ko05215	Prostate cancer	IGF1R,IGF1,TP53,E2F2,CCND1,CDKN1A,NRAS,BRAF,BCL2,MDM2,KRAS,FGFR1	5.42E-99
	ko05214	Glioma	IGF1R,IGF1,TP53,E2F2,CCND1,CDKN1A,NRAS,PLCG2,PLCG1,CDK4,BRAF,CDK6,MDM2,KRAS	5.06E-50
	ko05212	Pancreatic cancer	BCL2L1,TGFBR2,TGFBR1,TP53,E2F2,RAC1,CCND1,CDK4,BRAF,CDK6,JAK1,RALB,KRAS	1.15E-73
	ko05210	Colorectal cancer	TGFBR2,TGFBR1,TP53,CASP3,RAC1,CCND1,MSH6,BRAF,MYC,BCL2,KRAS	1.13E-71
	ko04730	Long-term depression	NOS1,IGF1R,IGF1,NRAS,BRAF,ITPR3,LYN,KRAS	5.05E-67
	ko04810	Regulation of actin cytoskeleton	ITGA8,LIMK2,ITGA3,MSN,WASF1,WASF2,NRAS,GNG12,ARHGEF1,PPP1R12A,FGFR1,ITGB3,DIAPH1,DIAPH2,RAC1,ITGB8,VCL,RDX,BCAR1,KRAS,ITGAV,BRAF,PAK1,PAK3	4.35E-174

	ko04660	T cell receptor signaling pathway	ICOS,RASGRP1,IL10,NRAS,FYN,CBL,PAK1,PLCG1,CD28,MAP2K7,CDK4,PAK3,PTPN6,KRAS	1.72E-113
	ko04672	Intestinal immune network for IgA production	ICOS,CD28,IL10,CD80,CCR10,IL15,CD86,CXCR4,ICOSLG	4.68E-56
	ko05330	Allograft rejection	IL10,CD80,CD28,PRF1,CD86	3.82E-38
	ko05332	Graft-versus-host disease	CD80,CD28,PRF1,CD86	9.82E-33
	ko05200	Pathways in cancer	BCL2L1,CDKN1A,ITGA3,TGFBR1,HIF1A,PLCG2,XIAP,RUNX1T1,CBL,FZD3,FZD4,NRAS,FADD,MYC,KRAS,TGFBR2,IGF1R,IGF1,CASP3,RAC1,JAK1,STK4,MSH6,SKP2,MDM2,LAMC1,ARNT2,FGFR1,TP53,E2F2,CCND1,MAX,PLCG1,ITGAV,RUNX1,CDK4,BRAF,CDK6,RALB,BCL2	4.59E-250
	ko05203	Viral carcinogenesis	PMAIP1,CDKN1A,CCR5,SRF,SKP2,NRAS,JAK1,LYN,DDB1,YWHAE,DDX3X,RAC1,MRPS18B,HPN,MDM2,KRAS,TP53,CASP3,CCND1,CDK4,CDK6,ATF6B	7.22E-165
	ko05414	Dilated cardiomyopathy (DCM)	ITGA8,EMD,ITGB3,IGF1,DMD,ADCY1,ITGB8,ITGA3,ADCY9,ITGAV,CACNB4,CACNG8,ADRB1,TNNC1	6.61E-115
	ko05416	Viral myocarditis	CD28,DMD,CASP3,RAC1,CCND1,CD80,FYN,CD86,PRF1,ABL2	1.78E-55
	ko04540	Gap junction	TJP1,ADCY1,MAP3K2,NRAS,ADCY9,TUBB,ADRB1,ITPR3,KRAS	2.55E-84
	ko04012	ErbB signaling pathway	AREG,CDKN1A,NRAS,CBL,PAK3,PLCG2,PLCG1,MAP2K7,BRAF,MAP2K4,EIF4EBP1,MYC,ABL2,PAK1,KRAS	7.37E-82
	ko04010	MAPK signaling pathway	MAP4K3,RASGRP1,RASGRP4,MAP2K7,MAP2K4,IL1R1,MAP3K2,MAP3K1,NRAS,GNG12,CACNB4,NF1,MYC,KRAS,TGFBR2,TGFBR1,SRF,RAC1,CDC25B,NR4A1,STK4,NLK,FGFR1,TP53,CASP3,MAX,CACNG8,BRAF,ELK4,PAK1	1.57E-204
	ko04151	PI3K-Akt signaling pathway	BCL2L1,ITGA8,CDKN1A,ITGA3,ITGB3,INSR,MCL1,TSC1,PRLR,NRAS,GNG12,OSMR,JAK1,CDK4,MYC,KRAS,YWHAE,IGF1R,IGF1,CDK6,RAC1,GNB4,GNB1,GNB2,MDM2,LAMC1,ATF6B,FGFR1,ITGB8,TP53,CCND1,CSF1,ITGAV,GNG5,EIF4EBP1,TLR4,LPAR2,VWF,BCL2	4.24E-235
	ko04110	Cell cycle	PCNA,CDC25A,TP53,BUB3,PLK1,CDC25B,CDKN1A,E2F2,MCM3,PRKDC,CCND1,WEE1,CDK4,CDK6,CDC45,SKP2,MYC,SMC3,MDM2,CDC6,YWHAE	6.41E-133
	ko04115	p53 signaling pathway	IGF1,PMAIP1,CASP3,CCND1,CDKN1A,CDK4,RRM2,CDK6,TP53,MDM2,MDM4,STEAP3,APAF1	1.51E-81
	ko05222	Small cell lung cancer	BCL2L1,SKP2,E2F2,CCND1,MAX,ITGA3,ITGAV,XIAP,CDK4,CDK6,TP53,MYC,LAMC1,APAF1,BCL2	5.99E-88
	ko05223	Non-small cell lung cancer	TP53,E2F2,CCND1,NRAS,PLCG2,PLCG1,STK4,BRAF,CDK6,CDK4,KRAS	2.36E-58
	ko05220	Chronic myeloid leukemia	BCL2L1,TGFBR2,TGFBR1,TP53,E2F2,CCND1,GAB2,CDKN1A,NRAS,CBL,RUNX1,CDK4,BRAF,CDK6,MDM2,MYC,KRAS	3.94E-65
	ko05221	Acute myeloid leukemia	CCND1,NRAS,RUNX1,EIF4EBP1,BRAF,RUNX1T1,MYC,KRAS	4.63E-72
	ko05014	Amyotrophic lateral sclerosis (ALS)	BCL2L1,NOS1,TNFRSF1B,CASP3,RAC1,DERL1,TP53,APAF1,BCL2	2.41E-66
	ko04520	Adherens junction	TGFBR2,IGF1R,TJP1,FARP2,WASF1,WASF2,RAC1,VCL,FYN,TGFBR1,PTPRJ,INSR,PVRL2,NLK,PTPN6,FGFR1	1.18E-76
	ko05161	Hepatitis B	CDKN1A,MAP2K4,STAT2,MAP3K1,NRAS,FADD,MYC,DDB1,APAF1,PCNA,TGFBR1,DDX3X,TLR4,JAK1,KRAS,E2F2,TP53,CASP3,CCND1,CDK4,CDK6,ATF6B,BCL2	2.92E-130
**hsa-let-7c-5p**	ko04141	Protein processing in endoplasmic reticulum	UBE2J1,SEC63,PRKCSH,MAP2K7,FBXO6,NPLOC4,XBP1,HERPUD1,DNAJB11,UBE2G2,CUL1,OS9,EDEM2,EDEM3,DNAJA2,UBE4B,ERO1L,MBTPS2,MBTPS1,EIF2AK1,PREB,ATF4,ATF6B,CKAP4,BCL2	2.18E-165
	ko04664	Fc epsilon RI signaling pathway	RAC1,NRAS,IL13,GAB2,PLCG2,MAP2K7,MAP2K4,LYN	9.51E-70
	ko04666	Fc gamma R-mediated phagocytosis	MARCKSL1,WASF2,RAC1,LIMK2,ARPC2,GAB2,ARF6,PLCG2,MARCKS,CRK,LYN,PAK1	2.77E-78
	ko05219	Bladder cancer	TP53,CCND1,CDKN1A,NRAS,RB1,CDK4,MYC,MDM2	6.83E-51
	ko05218	Melanoma	IGF1R,IGF1,TP53,CCND1,CDKN1A,NRAS,RB1,CDK4,CDK6,MDM2,FGFR1	1.03E-52
	ko05216	Thyroid cancer	TP53,CCND1,NRAS,TCF7L2,TPR,MYC	1.08E-46
	ko05215	Prostate cancer	IGF1R,IGF1,TP53,CCND1,CDKN1A,NRAS,RB1,FOXO1,TCF7L2,ATF4,MDM2,BCL2,FGFR1	8.42E-96
	ko05214	Glioma	IGF1R,IGF1,TP53,CCND1,CDKN1A,NRAS,RB1,PLCG2,CDK4,CDK6,MDM2	3.98E-56
	ko05213	Endometrial cancer	TP53,CCND1,NRAS,MLH1,FOXO3,TCF7L2,MYC	9.66E-64
	ko05212	Pancreatic cancer	BCL2L1,TGFBR2,TGFBR1,STAT3,RAC1,CCND1,RB1,CDK4,CDK6,JAK1,TP53	2.26E-77
	ko05210	Colorectal cancer	TGFBR2,TGFBR1,TP53,CASP3,RAC1,CCND1,TCF7L2,MSH6,MLH1,MYC,BCL2	5.39E-71
	ko04672	Intestinal immune network for IgA production	ICOS,CD28,IL10,CD80,CCR10,CD86,IL6,ICOSLG	6.64E-58
	ko05330	Allograft rejection	IL10,CD80,CD28,CD86	1.93E-40
	ko05332	Graft-versus-host disease	IL6,CD80,CD28,CD86	1.75E-32
	ko05200	Pathways in cancer	BCL2L1,PTGS2,PTK2,RB1,CDKN1A,ITGA3,TGFBR1,MSH6,PLCG2,FOXO1,RUNX1T1,SLC2A1,SKP2,CRK,FZD2,FZD3,STAT3,NRAS,MLH1,CBL,JAK1,MYC,CUL2,TGFBR2,IGF1R,IGF1,RAC1,STK4,TPR,FZD4,MDM2,LAMC1,FGFR1,TP53,CASP3,FN1,CCND1,MAX,TCF7L2,IL6,ITGAV,CDK4,CDK6,BCL2	9.81E-240
	ko05202	Transcriptional misregulation in cancers	BCL2L1,PTK2,CDKN1A,MEN1,FOXO1,RUNX1T1,NCOR1,IL6,SPINT1,CD86,MYC,DOT1L,SIN3A,TGFBR2,IGF1R,IGF1,LYL1,CEBPB,HMGA2,MDM2,PBX3,PBX1,TP53,MAX,CCND2,MEF2C,ELK4,EWSR1	1.50E-165
	ko05203	Viral carcinogenesis	PMAIP1,CDKN1A,STAT3,NRAS,RB1,JAK1,LYN,YWHAE,DDX3X,RBL2,RAC1,SKP2,MDM2,SNW1,TP53,CASP3,CCND1,CCND2,CDK4,CDK6,ATF4,ATF6B	6.40E-163
	ko05416	Viral myocarditis	CD28,CASP3,RAC1,CCND1,CD80,CD86,ABL2	4.99E-62
	ko04012	ErbB signaling pathway	PTK2,CDKN1A,NRAS,CBL,PLCG2,MAP2K7,MAP2K4,PAK4,CRK,MYC,ABL2,PAK1,PAK3	2.48E-85
	ko04010	MAPK signaling pathway	MAP4K3,RASGRP1,CACNG8,MAP2K7,RAPGEF2,MAP2K4,CRK,DAXX,MAP3K2,MAP3K1,NRAS,MAP3K7,CACNB4,MYC,TGFBR2,TGFBR1,RAC1,CDC25B,NR4A1,STK4,ATF4,NLK,FGFR1,TP53,CASP3,MAX,MEF2C,ELK4,FLNA,PAK1	6.63E-202
	ko04151	PI3K-Akt signaling pathway	BCL2L1,PTK2,CDKN1A,ITGA3,ITGB3,FOXO3,ITGA10,TSC1,BCL2L11,PRLR,NRAS,OSMR,JAK1,MYC,YWHAE,IGF1R,IGF1,CDK6,RBL2,RAC1,GNB1,IL6R,MDM2,LAMC1,FGFR1,TP53,FN1,CCND1,CCND2,INSR,ITGAV,IL6,GNG5,CDK4,TLR4,ATF4,ATF6B,LPAR1,BCL2	7.09E-232
	ko00563	Glycosylphosphatidylinositol(GPI)-anchor biosynthesis	PIGA,PIGC,PIGM,PIGP,PIGT,PIGW	5.76E-43

	ko04110	Cell cycle	WEE1,CDC25A,TP53,RBL2,CCND1,CDC25B,CDKN1A,CCND2,MCM3,RB1,CUL1,MCM6,MCM4,CDK4,CDK6,SKP2,MYC,PRKDC,SMC3,MDM2,YWHAE	2.03E-131
	ko04115	p53 signaling pathway	IGF1,PMAIP1,CASP3,CCND1,CDKN1A,CCND2,CCNG1,CDK4,RRM2,CDK6,TP53,MDM2,MDM4,STEAP3,APAF1	1.88E-76
	ko03013	RNA transport	EIF1,EIF2S3,EIF2S2,XPO1,NUP62,EIF3A,EIF3C,EIF3D,NDC1,SAP18,NUP214,RANBP2,CLNS1A,XPOT,NUP98,SNUPN,RAE1,NUPL1,TACC3,GEMIN4,NUP155,EIF4A3,TPR	3.73E-162
	ko05222	Small cell lung cancer	BCL2L1,PTGS2,PTK2,SKP2,FN1,CCND1,MAX,ITGA3,RB1,CDK4,ITGAV,CDK6,TP53,MYC,LAMC1,APAF1,BCL2	7.06E-83
	ko05223	Non-small cell lung cancer	TP53,CCND1,NRAS,RB1,FOXO3,PLCG2,STK4,CDK6,CDK4	2.20E-62
	ko05220	Chronic myeloid leukemia	BCL2L1,TGFBR2,TGFBR1,TP53,CCND1,GAB2,CDKN1A,NRAS,CBL,CDK4,RB1,CDK6,CRK,MDM2,MYC	1.16E-68
	ko05014	Amyotrophic lateral sclerosis (ALS)	BCL2L1,DAXX,TNFRSF1B,CASP3,RAC1,NOS1,TP53,APAF1,BCL2	9.78E-66
	ko04520	Adherens junction	TGFBR2,IGF1R,FARP2,WASF2,RAC1,PTPRJ,VCL,TGFBR1,TCF7L2,MAP3K7,INSR,NLK,SSX2IP,PTPN6,FGFR1	5.47E-78
	ko05161	Hepatitis B	CDKN1A,HSPG2,MAP2K4,STAT3,STAT2,TBK1,MAP3K1,NRAS,RB1,JAK1,MYC,APAF1,TGFBR1,DDX3X,TLR4,TP53,CASP3,CCND1,IL6,CDK4,CDK6,ATF4,ATF6B,MAVS,BCL2	6.31E-125
	ko05162	Measles	CD28,STAT3,STAT2,TBK1,MSN,CCND1,IL13,CCND2,MAP3K7,TNFAIP3,IL6,EIF2AK1,TNFSF10,CDK4,CDK6,TLR4,JAK1,TP53,MAVS,CD46	3.15E-128
	ko05169	Epstein-Barr virus infection	CDKN1A,PLCG2,MAP2K7,MAP2K4,NCOR2,XPO1,STAT3,TBK1,MAP3K7,RB1,JAK1,NUP214,MYC,YWHAE,HSPB2,SKP2,MDM2,LYN,SNW1,TP53,EIF2AK1,PSMD11,TNFAIP3,IL10,BCL2	5.47E-177
**hsa-miR-376c-3p**	ko05214	Glioma	TGFA,PDGFRA,CCND1,IGF1R,GRB2,MDM2,KRAS	9.76E-47
	ko05332	Graft-versus-host disease	IL1B,IL2,CD86	1.34E-26
**hsa-miR-377-3p**	ko04961	Endocrine and other factor-regulated calcium reabsorption	AP2M1,AP2S1,CLTA,ADCY6,CLTC	5.11E-40
	ko05219	Bladder cancer	RASSF1,RB1,CCND1,CDK4,MMP9,MYC,MMP2,MDM2,KRAS	1.54E-40
	ko05218	Melanoma	PDGFRA,CCND1,IGF1R,RB1,PTEN,CDK4,CDK6,MDM2,KRAS	1.26E-47
	ko05216	Thyroid cancer	CCND1,MYC,TCF7L2,RET,KRAS	1.53E-41
	ko05215	Prostate cancer	PDGFRA,CCND1,CDKN1B,IGF1R,RB1,GSK3B,IKBKB,PTEN,TCF7L2,MDM2,EP300,KRAS	1.53E-79
	ko05214	Glioma	PDGFRA,CCND1,IGF1R,RB1,PTEN,CDK4,CDK6,MDM2,KRAS	2.21E-50
	ko05213	Endometrial cancer	AXIN1,CCND1,TCF7L2,GSK3B,PTEN,MYC,KRAS	4.21E-53
	ko05212	Pancreatic cancer	BCL2L1,TGFBR2,RAC1,CCND1,RB1,IKBKB,CDK4,CDK6,KRAS	2.22E-67
	ko05210	Colorectal cancer	TGFBR2,AXIN1,RAC1,CCND1,BIRC5,MSH3,TCF7L2,GSK3B,MYC,KRAS	2.51E-60
	ko04350	TGF-beta signaling pathway	DCN,TGFBR2,ACVR2B,ACVR1,NODAL,FST,SP1,PITX2,BMPR2,MYC,EP300	1.14E-59
	ko05200	Pathways in cancer	BCL2L1,PDGFRA,RASSF1,CDKN1B,BIRC5,MSH3,ITGA6,IKBKB,XIAP,CRK,KITLG,EP300,FZD4,FZD6,RB1,RET,MYC,TGFBR2,IGF1R,RAC1,HIF1A,MDM2,LAMC1,KRAS,AXIN1,CCND1,MAX,TCF7L2,GSK3B,PTEN,RUNX1,CDK4,CDK6,MMP9,MMP2	2.17E-190
	ko04711	Circadian rhythm - fly	HLF,GSK3B	3.40E-14
	ko05222	Small cell lung cancer	BCL2L1,CCND1,MAX,RB1,IKBKB,PTEN,XIAP,CDK4,ITGA6,CDK6,MYC,LAMC1	4.61E-76
	ko05220	Chronic myeloid leukemia	BCL2L1,TGFBR2,CCND1,CDKN1B,RB1,IKBKB,RUNX1,CDK4,CDK6,CRK,MDM2,MYC,KRAS	1.08E-59
	ko05161	Hepatitis B	MMP9,STAT6,EP300,CCND1,CDKN1B,BIRC5,MAP3K1,RB1,IKBKB,PTEN,CDK4,CDK6,EGR3,MYC,EGR2,ATF6B,KRAS	1.62E-111
**hsa-miR-222-3p**	ko05216	Thyroid cancer	TP53,TFG,TPM3,CTNNB1,MYC,PAX8	4.57E-41
	ko05213	Endometrial cancer	CTNNB1,TP53,AXIN2,ILK,FOXO3,PTEN,MYC	1.38E-55
	ko04115	p53 signaling pathway	RCHY1,IGF1,TP53,CASP3,CDK1,PTEN,CDK6,BBC3,MDM4,MDM2,APAF1	3.00E-73
**hsa-miR-130a-3p**	ko04144	Endocytosis	TGFB1,PDGFRA,EHD4,TGFBR2,HRAS,EGFR,TGFB2,CLTC,CHMP7,AP2B1,EPS15,ERBB4,NEDD4L,CAV2,ERBB3,TGFBR1,RAB5A,RAB5C,RAB5B,RNF41,LDLR,RHOA,KIT,MET	1.13E-166
	ko04140	Regulation of autophagy	ATG14,ATG3,ATG13,BECN1	1.45E-38
	ko04662	B cell receptor signaling pathway	FOS,HRAS,NFKBIA,NRAS,GSK3B,MAP2K1,GRB2,NFKB1,LYN,RELA	2.97E-78
	ko04380	Osteoclast differentiation	TGFB1,FOSL2,TGFBR1,CTSK,MAP2K1,FOS,NFKBIA,CSF1,CREB1,SQSTM1,IFNAR2,TGFB2,GRB2,CYLD,MAP2K6,JAK1,NFKB1,RELA,MAP3K14,TGFBR2	1.10E-120
	ko04360	Axon guidance	ARHGEF12,ROBO2,DPYSL2,PAK6,EPHB4,EPHA7,ROBO1,RASA1,EPHA4,NRAS,EPHA2,GSK3B,MET,HRAS,ABL1,RHOA,NRP1,PAK3	8.16E-103
	ko05219	Bladder cancer	E2F2,EGFR,HRAS,NRAS,RB1,MAP2K1,CDK4,BRAF,MYC,CDKN1A	2.38E-46
	ko05218	Melanoma	PDGFRA,IGF1,E2F2,MET,EGFR,HRAS,NRAS,RB1,PTEN,MAP2K1,CDK4,BRAF,CDK6,CDKN1A,FGFR1	3.97E-44
	ko05216	Thyroid cancer	NCOA4,HRAS,NRAS,TCF7L2,MAP2K1,CCDC6,BRAF,MYC	6.24E-42
	ko05215	Prostate cancer	PDGFRA,CDKN1B,CDKN1A,NFKBIA,EGFR,MAP2K1,GSK3B,TGFA,NRAS,RB1,NFKB1,RELA,HRAS,IGF1,CREB5,CREB1,EP300,FGFR1,E2F2,TCF7L2,CASP9,PTEN,GRB2,BRAF	1.27E-73
	ko05214	Glioma	TGFA,PDGFRA,IGF1,E2F2,EGFR,HRAS,NRAS,RB1,PTEN,MAP2K1,GRB2,CDK4,BRAF,CDK6,CDKN1A	1.51E-47
	ko05213	Endometrial cancer	AXIN2,NRAS,HRAS,EGFR,ILK,TCF7L2,GSK3B,PTEN,MAP2K1,GRB2,BRAF,MYC,CASP9	3.89E-50
	ko05212	Pancreatic cancer	TGFA,TGFB1,TGFB2,STAT3,E2F2,TGFBR2,SMAD4,EGFR,TGFBR1,RB1,CASP9,MAP2K1,CDK4,BRAF,CDK6,JAK1,NFKB1,RELA	1.33E-62
	ko05211	Renal cell carcinoma	TGFA,TGFB1,TGFB2,MET,TCEB1,NRAS,BRAF,HIF1A,VHL,MAP2K1,GRB2,RAP1A,SLC2A1,PAK6,EP300,HRAS,PAK3	1.77E-61
	ko05210	Colorectal cancer	TGFB1,TGFB2,AXIN2,TGFBR2,SMAD4,FOS,TGFBR1,TCF7L2,CASP9,MAP2K1,BRAF,RHOA,GSK3B,MYC	1.39E-64
	ko04512	ECM-receptor interaction	LAMA4,FN1,ITGB8,HSPG2,SDC4,ITGA5,ITGA6,AGRN,DAG1,LAMC1	4.13E-86
	ko00785	Lipoic acid metabolism	LIPT1	2.97E-09
	ko04730	Long-term depression	IGF1,GRID2,HRAS,NRAS,MAP2K1,GNA13,LYN,ITPR1,BRAF	3.19E-64
	ko04210	Apoptosis	CASP6,NFKBIA,ATM,CASP8,CASP9,XIAP,MAP3K14,CAPN1,NFKB1,RELA,APAF1	1.56E-94

	ko04660	T cell receptor signaling pathway	DLG1,PAK6,ITK,FOS,CDK4,HRAS,NFKBIA,NRAS,GSK3B,CD4,MAP2K1,GRB2,MAP3K14,NFKB1,RELA,RHOA,PAK3	2.78E-106
	ko04916	Melanogenesis	EP300,FZD3,FZD4,FZD6,HRAS,NRAS,TCF7L2,GSK3B,MAP2K1,CREB1,KIT	1.56E-94
	ko04350	TGF-beta signaling pathway	TGFB1,TGFB2,ACVR1,TGFBR2,SMAD4,SP1,TGFBR1,BMPR2,MYC,EP300,ACVR2B,RHOA	1.48E-70
	ko05200	Pathways in cancer	TGFB1,PDGFRA,SMAD4,CDKN1B,TCEB1,NFKBIA,RASSF5,TGFB2,ITGA6,VHL,MAP2K1,RUNX1T1,SLC2A1,ABL1,CASP8,KIT,TGFA,FZD3,STAT3,AXIN2,NCOA4,PTEN,NRAS,RB1,CDKN1A,JAK1,NFKB1,RELA,TRAF4,HRAS,TGFBR2,TGFBR1,IGF1,LAMA4,FOS,EGFR,STK4,HIF1A,FZD4,RHOA,LAMC1,MYC,FGFR1,EP300,FZD6,FN1,CASP9,E2F2,XIAP,TCF7L2,GSK3B,MET,GRB2,CDK4,BRAF,CDK6,CCDC6	2.20E-219
	ko05203	Viral carcinogenesis	EIF2AK2,HDAC7,CDKN1B,HRAS,NFKBIA,IL6ST,DLG1,STAT3,NRAS,RB1,HNRNPK,JAK1,NFKB1,RELA,CDKN1A,CREB5,CREB1,SND1,RHOA,EP300,LYN,UBR4,TBP,CCND3,CCND2,CASP8,CDC20,CDK1,GRB2,CDK4,CDK6	2.21E-146
	ko05020	Prion diseases	C7,MAP2K1,LAMC1,PRNP,C5	3.61E-51
	ko05145	Toxoplasmosis	TGFB1,TGFB2,STAT3,IL10RB,LAMA4,NFKBIA,LDLR,ITGA6,CASP9,XIAP,CASP8,MAP2K6,JAK1,NFKB1,RELA,LAMC1,PPIF	3.95E-124
	ko04130	SNARE interactions in vesicular transport	VAMP1,VAMP3,STX6,STX11,STX16,GOSR1	1.18E-57
	ko04720	Long-term potentiation	ITPR1,HRAS,NRAS,RAP1A,MAP2K1,BRAF,EP300	2.69E-67
	ko04722	Neurotrophin signaling pathway	NGFRAP1,NRAS,HRAS,NFKBIA,PSEN1,RAP1A,GSK3B,MAP2K1,GRB2,BRAF,ABL1,NFKB1,RELA,MAP3K5,RHOA	1.93E-117
	ko04540	Gap junction	GJA1,PDGFRA,EGFR,HRAS,NRAS,MAP2K1,TUBB,CDK1,CSNK1D,GRB2,ITPR1	3.78E-79
	ko05152	Tuberculosis	TGFB1,TGFB2,IL10RB,NFYC,NFYA,CEBPG,JAK1,NFKB1,RELA,APAF1,ARHGEF12,RAB5A,RAB5C,RAB5B,CREB1,RHOA,EP300,RFXAP,IL18,RFX5,CASP8,CASP9	7.98E-160
	ko04510	Focal adhesion	PDGFRA,HRAS,EGFR,ITGA5,ITGA6,XIAP,CAV2,PTEN,IGF1,LAMA4,ITGB8,ILK,RAP1A,RHOA,LAMC1,FN1,CCND3,CCND2,MAP2K1,GSK3B,MET,GRB2,BRAF,PAK6,PAK3	8.43E-159
	ko04012	ErbB signaling pathway	TGFA,ERBB3,PAK6,ERBB4,EGFR,HRAS,NRAS,GSK3B,MAP2K1,GRB2,BRAF,ABL1,MYC,CDKN1A,PAK3	2.95E-81
	ko04013	MAPK signaling pathway - fly	MAP2K1,GRB2,EGFR	3.34E-33
	ko04151	PI3K-Akt signaling pathway	PDGFRA,CDKN1B,HRAS,EGFR,EPHA2,ITGA6,IFNAR2,MAP2K1,CRTC2,GSK3B,TSC1,TEK,BCL2L11,PTEN,NRAS,GNG12,OSMR,JAK1,NFKB1,RELA,MYB,CDKN1A,IGF1,LAMA4,ITGB8,CREB5,CHRM2,CREB1,ITGA5,LAMC1,MYC,FGFR1,FN1,CASP9,CCND3,CCND2,G6PC,CSF1,KIT,MET,GRB2,CDK4,CDK6	7.65E-226
	ko04110	Cell cycle	EP300,TGFB2,ATM,E2F2,TGFB1,SMAD4,CDKN1B,CDKN1A,CCND2,RB1,CDC20,MCM7,CDK1,CDK4,CDK6,ABL1,MYC,GSK3B,ORC6,CCND3	8.14E-134
	ko04115	p53 signaling pathway	CCND2,CDK1,CDKN1A,ATM,CCNG2,CASP8,CASP9,PTEN,ZMAT3,IGF1,CDK4,RRM2,CDK6,MDM4,CCND3,APAF1	1.50E-74
	ko05222	Small cell lung cancer	TRAF4,E2F2,FN1,LAMA4,NFKBIA,RB1,CASP9,PTEN,XIAP,CDK4,ITGA6,CDK6,NFKB1,MYC,LAMC1,RELA,APAF1	4.18E-83
	ko05223	Non-small cell lung cancer	TGFA,E2F2,NRAS,HRAS,RASSF5,RB1,CASP9,MAP2K1,GRB2,STK4,BRAF,CDK6,CDK4,EGFR	2.25E-51
	ko05220	Chronic myeloid leukemia	TGFB1,TGFB2,MYC,E2F2,TGFBR2,SMAD4,CDKN1B,HRAS,NFKBIA,NRAS,TGFBR1,RB1,MAP2K1,GRB2,CDK4,BRAF,CDK6,ABL1,NFKB1,RELA,CDKN1A	1.41E-56
	ko05221	Acute myeloid leukemia	STAT3,HRAS,NRAS,TCF7L2,KIT,MAP2K1,GRB2,RUNX1T1,BRAF,NFKB1,RELA,MYC	2.06E-62
	ko04612	Antigen processing and presentation	RFXAP,NFYC,KLRD1,RFX5,PDIA3,NFYA,IFI30,CD4,B2M,CREB1,CANX	5.84E-58
	ko04622	RIG-I-like receptor signaling pathway	TRIM25,NFKBIA,CASP8,CYLD,NFKB1,RELA,MAVS,RNF125,TBKBP1	3.76E-82
	ko04520	Adherens junction	TGFBR2,TGFBR1,SMAD4,EGFR,TCF7L2,MET,PTPRB,CTNND1,RHOA,EP300,PTPRJ,FGFR1	1.07E-84
	ko05164	Influenza A	NFKBIA,IFNAR2,MAP2K1,MAP2K6,DNAJC3,ADAR,JAK1,NFKB1,RELA,GSK3B,RNASEL,TRIM25,TMPRSS2,EP300,HNRNPUL1,IL18,EIF2AK2,CASP9,TLR7,KPNA1,MAVS,AGFG1	1.58E-137
	ko05166	HTLV-I infection	TGFB1,PDGFRA,TGFBR2,SMAD4,HRAS,ATM,TGFB2,CRTC3,CRTC2,CDC20,CANX,DLG1,FZD3,FZD4,FZD6,RAN,NRAS,RB1,NRP1,JAK1,NFKB1,RELA,MYB,MAP3K14,CDKN1A,XIAP,TGFBR1,NFKBIA,FOS,CREB1,TP53INP1,EP300,MYC,E2F2,TBP,CCND3,CCND2,IL15,SLC2A1,GSK3B,POLE4,CDK4,ELK4	1.16E-200
	ko05161	Hepatitis B	TGFB1,TGFB2,SMAD4,CDKN1B,CDKN1A,NFKBIA,MAP2K1,STAT3,NRAS,RB1,JAK1,NFKB1,RELA,HRAS,APAF1,TGFBR1,HSPG2,FOS,CREB5,CREB1,EP300,MYC,E2F2,CASP8,CASP9,PTEN,GRB2,CDK4,CDK6,MAVS	5.97E-116
	ko05160	Hepatitis C	MAVS,IRF1,NFKBIA,STAT3,NRAS,HRAS,EIF2AK2,EGFR,RNASEL,IFNAR2,LDLR,GRB2,BRAF,JAK1,NFKB1,RELA,GSK3B,CDKN1A	1.46E-98
	ko05162	Measles	EIF2AK2,NFKBIA,CDKN1B,CCND3,CCND2,NFKB1,GSK3B,IFNAR2,RAB9B,CDK4,TLR7,CDK6,JAK1,STAT3,RELA,MAVS,ADAR,TACR1	1.13E-132
**hsa-miR-143-3p**	ko05219	Bladder cancer	TP53,HRAS,NRAS,CDK4,BRAF,MDM2,MMP2,KRAS	1.15E-45
	ko05218	Melanoma	PDGFRA,TP53,HRAS,NRAS,IGF1R,PTEN,CDK4,BRAF,CDK6,MITF,MDM2,KRAS	4.66E-45
	ko05216	Thyroid cancer	TCF7,TP53,TPM3,HRAS,NRAS,CCDC6,BRAF,PAX8,KRAS	1.65E-35
	ko05215	Prostate cancer	PDGFRA,IGF1R,HRAS,NRAS,TCF7,PTEN,FOXO1,BRAF,BCL2,TP53,MDM2,EP300,KRAS	2.04E-84
	ko05214	Glioma	PDGFRA,TP53,HRAS,NRAS,IGF1R,PTEN,CDK4,BRAF,CDK6,MDM2,KRAS	4.49E-50
	ko05213	Endometrial cancer	TCF7,TP53,HRAS,NRAS,PTEN,ELK1,BRAF,KRAS	7.11E-55
	ko04730	Long-term depression	IGF1R,HRAS,NRAS,BRAF,LYN,ITPR1,KRAS	1.59E-61
	ko04540	Gap junction	PDGFRA,HRAS,NRAS,TUBB,CDK1,MAPK7,ITPR1,GJD2,KRAS	3.58E-74
	ko04115	p53 signaling pathway	TP53,IGFBP3,CCND2,CASP8,PTEN,PERP,MDM2,CDK6,CDK4,BBC3,SERPINE1,APAF1,CDK1	2.12E-71
	ko05222	Small cell lung cancer	PTGS2,TP53,FHIT,ITGA6,PTEN,XIAP,CDK4,CDK6,LAMC1,APAF1,BCL2	1.35E-84
	ko05223	Non-small cell lung cancer	TP53,FHIT,HRAS,NRAS,CDK4,BRAF,CDK6,KRAS	7.43E-58
	ko05220	Chronic myeloid leukemia	TP53,HRAS,NRAS,CBL,CDK4,BRAF,CDK6,MDM2,PTPN11,KRAS	1.06E-70

	ko05162	Measles	TLR2,CD28,TP53,IL2RA,IFNG,ADAR,CCND2,MAP3K7,TNFAIP3,IFNAR2,EIF2AK1,CDK4,CDK6,BBC3,EIF2AK2,EIF2AK3,TACR1,CD46	4.78E-115
hsa-miR-127-3p	No know pathways			
hsa-miR-221-3p	ko05219	Bladder cancer	TP53,CCND1,MMP2,RB1,MDM2,RAF1,MMP1	5.16E-47
	ko05218	Melanoma	IGF1,TP53,CCND1,RB1,PTEN,CDK6,MITF,MDM2,RAF1	2.77E-50
	ko05216	Thyroid cancer	TP53,CCND1,TFG,TPM3,TCF7L1,TCF7L2,CTNNB1,PAX8	5.36E-37
	ko05215	Prostate cancer	IGF1,TP53,CCND1,CDKN1B,CREB1,TCF7L1,RB1,CTNNB1,PTEN,TCF7L2,ATF4,MDM2,RAF1	1.20E-82
	ko05214	Glioma	IGF1,TP53,CCND1,RB1,PTEN,CDK6,MDM2,RAF1	2.48E-55
	ko05213	Endometrial cancer	CTNNB1,TP53,AXIN2,CCND1,TCF7L1,TCF7L2,FOXO3,PTEN,RAF1	9.31E-52
	ko05210	Colorectal cancer	TP53,AXIN2,FOS,CCND1,TCF7L1,MSH6,CTNNB1,TCF7L2,RHOA,RAF1,CASP3	7.41E-62
	ko05203	Viral carcinogenesis	CDKN2B,PXN,LYN,DDX3X,TP53,RB1,CCND1,CDKN1B,CCND2,CASP3,CREB1,CDK1,STAT5A,JAK3,CDK6,ATF4,STAT3,MDM2,YWHAE,RHOA,UBR4	1.33E-138
	ko04510	Focal adhesion	ITGA8,ITGB3,IGF1,BCAR1,CCND1,RAF1,CCND2,ITGB8,PTK2,CTNNB1,PTEN,KDR,PAK2,FLNA,ARHGAP5,RHOA,LAMC1,CAV2,PAK1,ZYX,PXN	1.43E-139
	ko04115	p53 signaling pathway	RCHY1,IGF1,TP53,CASP3,CDK1,CCND1,CCND2,PTEN,CDK6,BBC3,MDM4,MDM2,APAF1	6.01E-70
	ko05220	Chronic myeloid leukemia	TP53,CCND1,CDKN1B,PTPN11,CBL,STAT5A,RUNX1,RB1,CDK6,MDM2,RAF1	2.77E-67
	ko05221	Acute myeloid leukemia	STAT3,CCND1,TCF7L1,TCF7L2,KIT,STAT5A,RUNX1,RUNX1T1,RAF1	2.19E-60
	ko04612	Antigen processing and presentation	NFYC,CREB1,TAPBP,CD4,NFYB,NFYA,CANX	6.00E-59
	ko04520	Adherens junction	PTPRJ,TCF7L1,TCF7L2,SSX2IP,CTNNB1,PTPRF,BAIAP2,RHOA,PTPRM,NLK	3.15E-77
hsa-miR-12136	No know pathways			
hsa-miR-22-3p	ko04144	Endocytosis	TGFB1,EHD4,EHD2,EHD1,SRC,NEDD4,IGF1R,CLTC,CAV3,CSF1R,AP2B1,RAB11A,TFRC,RABEP1,KDR,ERBB3,TGFBR1,ERBB4,RAB5B,RUFY1,FGFR2,FGFR3,ZFYVE20,CBL,GIT1,RAB7A,LDLRAP1	6.76E-166
	ko04962	Vasopressin-regulated water reabsorption	ADCY6,GNAS,RAB5B,STX4,CREB1,ADCY9,RAB11A	1.37E-54
	ko04961	Endocrine and other factor-regulated calcium reabsorption	ADCY6,GNAQ,GNAS,ADCY9,RAB11A,CLTC,AP2B1	2.22E-43
	ko05219	Bladder cancer	TP53,E2F2,CDKN1A,NRAS,MYC,RAF1,FGFR3	1.37E-54
	ko05218	Melanoma	IGF1R,TP53,E2F2,CDKN1A,NRAS,PTEN,CDK6,RAF1,FGFR1	1.00E-58
	ko05217	Basal cell carcinoma	TCF7,FZD4,AXIN1,FZD6,WNT3,WNT1,TCF7L2,GSK3B,SHH,TP53,LEF1,WNT4	2.67E-55
	ko05216	Thyroid cancer	TCF7,TP53,TPM3,NRAS,TCF7L2,PAX8,LEF1,MYC	7.78E-43
	ko05215	Prostate cancer	TCF7,TP53,E2F2,FGFR2,CDKN1A,NRAS,IGF1R,TCF7L2,GSK3B,IKBKB,PTEN,CREB1,PDPK1,LEF1,EP300,RAF1,RELA,FGFR1	9.43E-88
	ko05214	Glioma	IGF1R,TP53,E2F2,CDKN1A,NRAS,PTEN,CDK6,RAF1	1.23E-64
	ko05213	Endometrial cancer	TCF7,TP53,AXIN1,NRAS,TCF7L2,GSK3B,FOXO3,PTEN,PDPK1,MYC,RAF1,LEF1	1.51E-53
	ko05212	Pancreatic cancer	TGFB1,TGFBR1,STAT3,E2F2,STAT1,RAC1,IKBKB,CDK6,JAK1,TP53,RELA,RAF1	3.49E-77
	ko05211	Renal cell carcinoma	TGFB1,RAC1,JUN,NRAS,HIF1A,RAPGEF1,PAK4,EP300,RAF1,SLC2A1,PAK3	3.45E-76
	ko05210	Colorectal cancer	TGFB1,TGFBR1,TP53,AXIN1,RAC1,BIRC5,JUN,TCF7,MSH6,GSK3B,TCF7L2,MYC,RAF1,LEF1	3.67E-66
	ko05131	Shigellosis	SRC,CTTN,DIAPH1,WASF2,RAC1,VCL,ITGA5,ARPC5,IKBKB,ABL1,RELA	3.10E-92
	ko04730	Long-term depression	IGF1R,GNAQ,GNAS,RYR1,NRAS,GNA11,ITPR3,RAF1	4.14E-68
	ko04810	Regulation of actin cytoskeleton	ITGA5,ITGA6,ITGB3,MSN,RAF1,SLC9A1,WASF2,ARHGEF4,RAC1,NRAS,PPP1R12A,SRC,ARHGEF12,ITGB5,DIAPH1,PIKFYVE,VCL,ARPC5,FGFR2,FGFR3,FGFR1,GIT1,PAK4,PAK3	7.58E-178
	ko04210	Apoptosis	RIPK1,TP53,TNFRSF1A,IKBKB,ENDOG,DFFA,CASP10,XIAP,CAPN1,RELA,MYD88,APAF1	1.24E-94
	ko04912	GnRH signaling pathway	SRC,ADCY6,GNAQ,GNAS,MAP3K3,GNA11,JUN,ADCY9,MAP2K4,ITPR3,RAF1,NRAS	2.19E-88
	ko04910	Insulin signaling pathway	SOCS4,GCK,NRAS,CBL,GSK3B,IKBKB,PTPRF,FASN,RAPGEF1,PDPK1,PTPN1,RAF1,TSC1	1.86E-113
	ko04916	Melanogenesis	TCF7,FZD4,GNAQ,FZD6,GNAS,WNT3,WNT1,NRAS,ADCY6,TCF7L2,GSK3B,ADCY9,WNT4,CREB1,POMC,LEF1,EP300,RAF1	1.29E-81
	ko04350	TGF-beta signaling pathway	TGFB1,TGFBR1,ACVR1,RBL1,SKP1,SP1,ACVR2B,BMPR1B,CHRD,BMPR2,MYC,EP300,CUL1,ACVR2A	8.12E-68
	ko05200	Pathways in cancer	TGFB1,MYC,MSH6,CSF1R,CDKN1A,TCF7,ITGA6,RAC1,IKBKB,XIAP,TCF7L2,AXIN1,ABL1,WNT4,RAF1,FLT3,CEBPA,EP300,STAT3,STAT1,STAT5B,NRAS,JUN,FGFR3,CBL,JAK1,RELA,BIRC5,TGFBR1,IGF1R,TRAF3,TPM3,FLT3LG,HIF1A,SHH,FZD4,LEF1,LAMC1,FGFR2,PAX8,FGFR1,TP53,FZD6,WNT3,MAX,WNT1,E2F2,SLC2A1,GSK3B,PTEN,CDK6	2.32E-237
	ko05202	Transcriptional misregulation in cancers	CDKN1A,REL,FLT3,CEBPA,SIX4,GOLPH3,CSF1R,BMP2K,KLF3,MYC,SIN3A,IGF1R,SP1,FLI1,PAX5,PAX8,PBX1,ETV1,RELA,TP53,HMGA2,WHSC1,RUNX2,DDX5,ELK4,MAX,EWSR1	3.44E-173
	ko05203	Viral carcinogenesis	HDAC7,NRAS,CDKN1A,REL,SRF,MAPKAPK2,STAT3,CHD4,STAT5B,UBE3A,JUN,JAK1,RELA,DDB1,YWHAE,SRC,TRAF3,DDX3X,RBL1,RAC1,CREB1,EP300,RASA2,UBR4,IRF3,TP53,CDK1,CDK6	1.41E-156
	ko04310	Wnt signaling pathway	GSK3B,FZD4,AXIN1,SKP1,CHD8,PSEN1,JUN,MAP3K7,CSNK1E,MYC,CUL1,TCF7,RAC1,LEF1,EP300,TP53,FZD6,WNT3,WNT1,TCF7L2,WNT4,FRAT2	2.15E-121
	ko04722	Neurotrophin signaling pathway	MAPKAPK2,FOXO3,TP53,RAC1,MAP3K3,PSEN1,NRAS,JUN,GSK3B,IKBKB,RAPGEF1,MAGED1,ABL1,NTRK3,BDNF,FRS2,RAF1,RELA,YWHAE	3.87E-112
	ko04540	Gap junction	SRC,ADCY6,GNAQ,GNAS,DRD2,NRAS,ADCY9,TUBB,CDK1,CSNK1D,GNA11,ITPR3,RAF1	1.45E-76
	ko00533	Glycosaminoglycan biosynthesis - keratan sulfate	CHST1,CHST2,B4GALT2	2.13E-27
	ko00532	Glycosaminoglycan biosynthesis - chondroitin sulfate	CHST11,CHPF2,CHST12,CHST15,CHST3	1.11E-36
	ko04340	Hedgehog signaling pathway	WNT3,WNT1,WNT4,CSNK1D,CSNK1E,SHH,GSK3B	1.42E-63

	ko04510	Focal adhesion	ITGA5,ITGA6,ITGB3,XIAP,RAPGEF1,FLT4,RAF1,CAV3,ZYX,JUN,PPP1R12A,KDR,SRC,IGF1R,ITGB5,DIAPH1,RAC1,VCL,PDPK1,LAMC1,GSK3B,PTEN,PAK4,FLNA,PAK3	1.25E-163
	ko04012	ErbB signaling pathway	ERBB3,NRG2,ERBB4,SRC,JUN,CDKN1A,NRAS,CBL,GSK3B,STAT5B,PAK4,MAP2K4,ABL1,MYC,ABL2,RAF1,PAK3	4.79E-79
	ko04010	MAPK signaling pathway	TGFB1,MYC,STMN1,NRAS,IKBKB,MAP2K4,BDNF,RAF1,MAPKAPK2,PPM1B,PPM1A,MAP3K3,JUN,MAP3K7,RELA,FGFR1,TGFBR1,SRF,MAPT,RAC1,CDC25B,FGFR2,FGFR3,RASA2,MAPK8IP3,TP53,RASGRF2,TNFRSF1A,MAX,ELK4,FLNA	2.42E-207
	ko04151	PI3K-Akt signaling pathway	MYC,GHR,NRAS,CDKN1A,EPHA2,ITGA6,IKBKB,MCL1,ITGB5,FLT4,RAF1,TSC1,IL6R,TEK,PRLR,CSF1R,GNG11,JAK1,ANGPT2,RELA,KDR,YWHAE,IGF1R,DDIT4,RAC1,GNB5,GNB4,CREB1,ITGA5,LAMC1,FGFR2,FGFR3,FGFR1,FOXO3,TP53,SGK1,ITGB3,GSK3B,PTEN,CDK6,PDPK1	2.54E-237
	ko00563	Glycosylphosphatidylinositol(GPI)-anchor biosynthesis	PIGP,PIGV,PIGC,DPM2,PIGN	1.54E-46
	ko04711	Circadian rhythm - fly	CSNK1E,GSK3B	1.65E-16
	ko04710	Circadian rhythm - mammal	NR1D1,CSNK1D,CSNK1E,CUL1,SKP1	1.56E-30
	ko04971	Gastric acid secretion	CCKBR,ADCY6,SLC9A1,GNAS,ADCY9,SLC4A2,ITPR3,CFTR,GNAQ	1.56E-72
	ko04972	Pancreatic secretion	ADCY6,SLC9A1,RAC1,GNAS,CCKAR,ADCY9,RAB11A,SLC4A2,ITPR3,CFTR,GNAQ	6.56E-97
	ko04370	VEGF signaling pathway	SRC,RAC1,NRAS,RAF1,MAPKAPK2,KDR	6.44E-59
	ko04110	Cell cycle	TGFB1,BUB1B,CDKN1A,PRKDC,ABL1,CDC6,SKP1,CDH1,WEE1,CUL1,YWHAE,RBL1,CDC25B,MCM7,MCM6,MCM5,MCM4,EP300,MYC,TP53,E2F2,GSK3B,CDK1,CDK6	1.35E-129
	ko04270	Vascular smooth muscle contraction	ADORA2A,ADORA2B,ARHGEF12,ADCY6,GNAS,RAMP1,ADCY9,MYL6,PPP1R12A,GNA11,ITPR3,RAF1,NPPC,GNAQ	6.12E-120
	ko05222	Small cell lung cancer	TRAF3,TP53,E2F2,MAX,ITGA6,IKBKB,PTEN,XIAP,CDK6,MYC,LAMC1,RELA,APAF1	6.22E-94
	ko05223	Non-small cell lung cancer	TP53,E2F2,NRAS,FOXO3,CDK6,PDPK1,RAF1	7.89E-69
	ko05220	Chronic myeloid leukemia	TGFB1,TGFBR1,TP53,E2F2,CDKN1A,NRAS,CBL,IKBKB,STAT5B,CDK6,ABL1,RELA,RAF1,MYC	9.85E-73
	ko05221	Acute myeloid leukemia	CEBPA,MYC,STAT3,NRAS,TCF7,TCF7L2,IKBKB,STAT5B,LEF1,RELA,RAF1,FLT3	6.71E-64
	ko04612	Antigen processing and presentation	RFXANK,CIITA,CTSB,NFYA,CREB1,B2M,RFX5,CANX	2.23E-66
	ko04620	Toll-like receptor signaling pathway	IRF3,LBP,RIPK1,TRAF3,STAT1,IRF5,RAC1,JUN,MAP3K7,IKBKB,MAP2K4,RELA,MYD88,TLR8	2.65E-108
	ko04622	RIG-I-like receptor signaling pathway	IRF3,IKBKB,TRAF3,DDX3X,MAP3K7,RIPK1,CASP10,RELA,MAVS	3.95E-84
	ko04520	Adherens junction	SRC,IGF1R,SSX2IP,WASF2,RAC1,VCL,TGFBR1,TCF7L2,MAP3K7,PTPRF,TCF7,PTPN1,EP300,LEF1,FGFR1	3.56E-80
	ko05164	Influenza A	IRF3,MYD88,NLRP3,STAT1,TNFRSF1A,CIITA,EP300,MAP2K4,JUN,DNAJC3,GSK3B,IKBKB,KPNA2,JAK1,RELA,MAVS,RAF1,EIF2S1,HNRNPUL1,AGFG1	1.35E-145
	ko05166	HTLV-I infection	TGFB1,BUB1B,NRAS,CDKN1A,IKBKB,XIAP,MAP2K4,WNT4,CANX,FZD4,FZD6,MAP3K3,EGR1,JUN,POLD2,JAK1,MYC,TGFBR1,SRF,CREB1,TP53INP1,TRRAP,EP300,RELA,ADCY6,TP53,E2F2,TNFRSF1A,WNT3,WNT1,ADCY9,SLC2A1,GSK3B,STAT5B,ELK4,ATF3	5.14E-220
	ko05161	Hepatitis B	TGFB1,NRAS,CDKN1A,HSPG2,IKBKB,MAP2K4,RAF1,STAT3,STAT1,STAT5B,JUN,JAK1,MYC,MYD88,BIRC5,APAF1,SRC,TGFBR1,DDX3X,CREB1,EP300,RELA,IRF3,CASP10,DDB1,TP53,E2F2,PTEN,CDK6,MAVS	2.19E-119
	ko05160	Hepatitis C	IRF3,RIPK1,TRAF3,STAT3,STAT1,TNFRSF1A,RAF1,CDKN1A,NRAS,GSK3B,IKBKB,JAK1,OCLN,TP53,RELA,MAVS,EIF2S1	2.61E-103
	ko05169	Epstein-Barr virus infection	PSMD8,JUN,CDKN1A,PSMD6,PSMD2,IKBKB,MAP2K4,GSK3B,NCOR2,STAT3,NEDD4,MAP3K7,JAK1,NUP214,RELA,YWHAE,TRAF3,HSPB2,EP300,MYC,IRF3,RIPK1,TP53,FCER2,PSMD11,VIM,CDK1	3.44E-179

Supplementary Table S5. Consensus pathways using miRPathDB v2.0 and EVmiRNAs downstream of up and down regulated miRNAs in nicotine-treated DPSCs vs control.

				**ND: No data**
	**mirPathDB v2.0_UP regulated miRNAs**	**EVmiRNA_UP regulated miRNAs**	**mirPathDB v2.0_DOWN regulated miRNAs**	**EVmiRNA_DOWN regulated miRNAs**
1	Acute myeloid leukemia	Acute myeloid leukemia	Acute myeloid leukemia	Acute myeloid leukemia
2	ND	Adherens junction	Adherens junction	Adherens junction
3	ND	ND	Adipocytokine signaling pathway	ND
4	Adrenergic signaling in cardiomyocytes	ND	Adrenergic signaling in cardiomyocytes	ND
5	African trypanosomiasis	ND	ND	ND
6	ND	ND	Alcoholism	ND
7	ND	ND	Aldosterone-regulated sodium reabsorption	ND
8	ND	Allograft rejection	ND	ND
9	Amoebiasis	ND	Amoebiasis	ND
10	ND	ND	Amphetamine addiction	ND
11	ND	Amyotrophic lateral sclerosis (ALS)	Amyotrophic lateral sclerosis (ALS)	ND
12	ND	ND	ND	Antigen processing and presentation
13	Apoptosis	Apoptosis	Apoptosis	Apoptosis
14	ND	ND	Arachidonic acid metabolism	ND
15	Arrhythmogenic right ventricular cardiomyopathy (ARVC)	ND	ND	ND
16	ND	ND	Ascorbate and aldarate metabolism	ND
17	ND	Autoimmune thyroid disease	ND	ND
18	Axon guidance	Axon guidance	Axon guidance	Axon guidance
19	B cell receptor signaling pathway	B cell receptor signaling pathway	B cell receptor signaling pathway	B cell receptor signaling pathway
20	ND	Bacterial invasion of epithelial cells	Bacterial invasion of epithelial cells	ND
21	ND	Basal cell carcinoma	ND	Basal cell carcinoma
22	ND	Base excision repair	ND	ND
23	Biosynthesis of amino acids	ND	ND	ND
24	Bladder cancer	Bladder cancer	Bladder cancer	Bladder cancer
25	Calcium signaling pathway	ND	Calcium signaling pathway	ND
26	ND	ND	Carbohydrate digestion and absorption	ND
27	Carbon metabolism	ND	ND	ND
28	Cardiac muscle contraction	ND	ND	ND
29	Cell cycle	Cell cycle	Cell cycle	Cell cycle
30	ND	Chagas disease (American trypanosomiasis	Chagas disease (American trypanosomiasis)	ND
31	ND	ND	Chemical carcinogenesis	ND
32	Chemokine signaling pathway	Chemokine signaling pathway	Chemokine signaling pathway	ND
33	ND	Cholinergic synapse	Cholinergic synapse	ND
34	Chronic myeloid leukemia	Chronic myeloid leukemia	Chronic myeloid leukemia	Chronic myeloid leukemia
35	Circadian entrainment	ND	Circadian entrainment	ND
36	ND	ND	ND	Circadian rhythm - fly
37	ND	ND	ND	Circadian rhythm - mammal
38	ND	ND	Cocaine addiction	ND
39	ND	ND	Collecting duct acid secretion	ND
40	Colorectal cancer	Colorectal cancer	Colorectal cancer	Colorectal cancer
41	Cytokine-cytokine receptor interaction	ND	Cytokine-cytokine receptor interaction	ND
42	ND	ND	Dilated cardiomyopathy	ND
43	ND	Dilated cardiomyopathy (DCM)	ND	ND
44	ND	ND	DNA replication	ND
45	ND	ND	Dopaminergic synapse	ND
46	Dorso-ventral axis formation	ND	ND	ND
47	ND	ND	Drug metabolism - cytochrome P450	ND
48	ND	ND	Drug metabolism - other enzymes	ND
49	ND	ND	ECM-receptor interaction	ECM-receptor interaction
50	ND	ND	ND	Endocrine and other factor-regulated calcium reabsorption
51	ND	Endocytosis	Endocytosis	Endocytosis
52	Endometrial cancer	Endometrial cancer	Endometrial cancer	Endometrial cancer
53	Epithelial cell signaling in Helicobacter pylori infection	Epithelial cell signaling in Helicobacter pyl	Epithelial cell signaling in Helicobacter pylori infection	ND
54	Epstein-Barr virus infection	Epstein-Barr virus infection	Epstein-Barr virus infection	Epstein-Barr virus infection
55	ErbB signaling pathway	ErbB signaling pathway	ErbB signaling pathway	ErbB signaling pathway
56	Estrogen signaling pathway	ND	Estrogen signaling pathway	ND
57	Fc epsilon RI signaling pathway	Fc epsilon RI signaling pathway	Fc epsilon RI signaling pathway	ND
58	ND	Fc gamma R-mediated phagocytosis	Fc gamma R-mediated phagocytosis	ND
59	Focal adhesion	Focal adhesion	Focal adhesion	Focal adhesion
60	FoxO signaling pathway	ND	FoxO signaling pathway	ND
61	GABAergic synapse	ND	GABAergic synapse	ND
62	Gap junction	Gap junction	Gap junction	Gap junction
63	ND	ND	ND	Gastric acid secretion
64	Glioma	Glioma	Glioma	Glioma
65	ND	ND	Glutamatergic synapse	ND
66	ND	ND	ND	Glycosaminoglycan biosynthesis - chondroitin sulfate
67	ND	ND	Glycosaminoglycan biosynthesis - chondroitin sulfate / derm	ND
68	Glycosaminoglycan biosynthesis - heparan sulfate / heparin	ND	ND	ND
69	ND	Glycosaminoglycan biosynthesis - keratan s	ND	Glycosaminoglycan biosynthesis - keratan sulfate
70	ND	Glycosaminoglycan degradation	ND	ND
71	ND	Glycosphingolipid biosynthesis - lacto and	ND	ND
72	ND	Glycosylphosphatidylinositol(GPI)-anchor	ND	Glycosylphosphatidylinositol(GPI)-anchor biosynthesis
73	GnRH signaling pathway	GnRH signaling pathway	GnRH signaling pathway	GnRH signaling pathway
74	ND	Graft-versus-host disease	ND	Graft-versus-host disease
75	ND	ND	ND	Hedgehog signaling pathway
76	Hepatitis B	Hepatitis B	Hepatitis B	Hepatitis B
77	Hepatitis C	Hepatitis C	Hepatitis C	Hepatitis C
78	ND	Herpes simplex infection	ND	ND
79	HIF-1 signaling pathway	ND	HIF-1 signaling pathway	ND
80	Hippo signaling pathway	ND	Hippo signaling pathway	ND
81	HTLV-I infection	HTLV-I infection	HTLV-I infection	HTLV-I infection
82	ND	ND	Huntington's disease	ND
83	Hypertrophic cardiomyopathy (HCM)	ND	Hypertrophic cardiomyopathy (HCM)	ND
84	Inflammatory bowel disease (IBD)	ND	Inflammatory bowel disease (IBD)	ND
85	ND	Influenza A	Influenza A	Influenza A
86	Insulin secretion	ND	ND	ND
87	Insulin signaling pathway	Insulin signaling pathway	Insulin signaling pathway	Insulin signaling pathway
88	Intestinal immune network for IgA production	Intestinal immune network for IgA producti	ND	ND
89	Jak-STAT signaling pathway	Jak-STAT signaling pathway	ND	ND

90	ND	ND	Legionellosis	ND
91	ND	ND	Leishmaniasis	ND
92	ND	ND	Leukocyte transendothelial migration	ND
93	ND	ND	ND	Lipoic acid metabolism
94	Long-term depression	Long-term depression	Long-term depression	Long-term depression
95	Long-term potentiation	Long-term potentiation	Long-term potentiation	Long-term potentiation
96	ND	Lysine degradation	ND	ND
97	ND	ND	Malaria	ND
98	MAPK signaling pathway	MAPK signaling pathway	MAPK signaling pathway	MAPK signaling pathway
99	ND	ND	ND	MAPK signaling pathway - fly
100	Measles	Measles	Measles	Measles
101	Melanogenesis	ND	ND	Melanogenesis
102	Melanoma	Melanoma	Melanoma	Melanoma
103	ND	ND	Metabolism of xenobiotics by cytochrome P450	ND
104	MicroRNAs in cancer	ND	MicroRNAs in cancer	ND
105	Morphine addiction	ND	Morphine addiction	ND
106	ND	ND	mRNA surveillance pathway	ND
107	ND	ND	mTOR signaling pathway	ND
108	ND	Mucin type O-glycan biosynthesis	ND	ND
109	Natural killer cell mediated cytotoxicity	Natural killer cell mediated cytotoxicity	Natural killer cell mediated cytotoxicity	ND
110	Neurotrophin signaling pathway	Neurotrophin signaling pathway	Neurotrophin signaling pathway	Neurotrophin signaling pathway
111	NF-kappa B signaling pathway	NF-kappa B signaling pathway	NF-kappa B signaling pathway	ND
112	ND	ND	NOD-like receptor signaling pathway	ND
113	ND	ND	Non-alcoholic fatty liver disease (NAFLD)	ND
114	ND	Non-homologous end-joining	ND	ND
115	Non-small cell lung cancer	Non-small cell lung cancer	Non-small cell lung cancer	Non-small cell lung cancer
116	Notch signaling pathway	ND	ND	ND
117	Oocyte meiosis	Oocyte meiosis	Oocyte meiosis	ND
118	ND	Osteoclast differentiation	Osteoclast differentiation	Osteoclast differentiation
119	Ovarian steroidogenesis	ND	Ovarian steroidogenesis	ND
120	p53 signaling pathway	p53 signaling pathway	p53 signaling pathway	p53 signaling pathway
121	Pancreatic cancer	Pancreatic cancer	Pancreatic cancer	Pancreatic cancer
122	ND	ND	ND	Pancreatic secretion
123	Pathogenic Escherichia coli infection	ND	Pathogenic Escherichia coli infection	ND
124	Pathways in cancer	Pathways in cancer	Pathways in cancer	Pathways in cancer
125	ND	ND	Pentose and glucuronate interconversions	ND
126	Pentose phosphate pathway	ND	ND	ND
127	ND	ND	Peroxisome	ND
128	Pertussis	ND	Pertussis	ND
129	PI3K-Akt signaling pathway	PI3K-Akt signaling pathway	PI3K-Akt signaling pathway	PI3K-Akt signaling pathway
130	ND	ND	Porphyrin and chlorophyll metabolism	ND
131	ND	Primary immunodeficiency	ND	ND
132	ND	ND	ND	Prion diseases
133	Progesterone-mediated oocyte maturation	ND	Progesterone-mediated oocyte maturation	ND
134	Prolactin signaling pathway	ND	Prolactin signaling pathway	ND
135	Prostate cancer	Prostate cancer	Prostate cancer	Prostate cancer
136	ND	Proteasome	ND	ND
137	ND	ND	Protein digestion and absorption	ND
138	ND	Protein processing in endoplasmic reticulur	Protein processing in endoplasmic reticulum	ND
139	Proteoglycans in cancer	ND	Proteoglycans in cancer	ND
140	Rap1 signaling pathway	ND	Rap1 signaling pathway	ND
141	Ras signaling pathway	ND	Ras signaling pathway	ND
142	Regulation of actin cytoskeleton	Regulation of actin cytoskeleton	Regulation of actin cytoskeleton	Regulation of actin cytoskeleton
143	ND	ND	ND	Regulation of autophagy
144	Renal cell carcinoma	Renal cell carcinoma	Renal cell carcinoma	Renal cell carcinoma
145	ND	ND	Retinol metabolism	ND
146	ND	ND	Retrograde endocannabinoid signaling	ND
147	ND	ND	Rheumatoid arthritis	ND
148	Ribosome	ND	Ribosome	ND
149	ND	RIG-I-like receptor signaling pathway	ND	RIG-I-like receptor signaling pathway
150	ND	RNA transport	ND	ND
151	ND	Salivary secretion	ND	ND
152	ND	ND	Salmonella infection	ND
153	Serotonergic synapse	Serotonergic synapse	Serotonergic synapse	ND
154	ND	Shigellosis	Shigellosis	Shigellosis
155	ND	Small cell lung cancer	Small cell lung cancer	Small cell lung cancer
156	SNARE interactions in vesicular transport	ND	SNARE interactions in vesicular transport	SNARE interactions in vesicular transport
157	ND	ND	Sphingolipid metabolism	ND
158	ND	Spliceosome	ND	ND
159	ND	ND	Starch and sucrose metabolism	ND
160	ND	ND	Steroid hormone biosynthesis	ND
161	ND	T cell receptor signaling pathway	T cell receptor signaling pathway	T cell receptor signaling pathway
162	TGF-beta signaling pathway	TGF-beta signaling pathway	TGF-beta signaling pathway	TGF-beta signaling pathway
163	Thyroid cancer	Thyroid cancer	Thyroid cancer	Thyroid cancer
164	Thyroid hormone signaling pathway	ND	Thyroid hormone signaling pathway	ND
165	ND	Tight junction	Tight junction	ND
166	TNF signaling pathway	ND	TNF signaling pathway	ND
167	ND	Toll-like receptor signaling pathway	Toll-like receptor signaling pathway	Toll-like receptor signaling pathway
168	Toxoplasmosis	Toxoplasmosis	Toxoplasmosis	Toxoplasmosis
169	Transcriptional misregulation in cancer	ND	Transcriptional misregulation in cancer	ND
170	ND	Transcriptional misregulation in cancers	ND	Transcriptional misregulation in cancers
171	Tuberculosis	ND	Tuberculosis	Tuberculosis
172	ND	Type I diabetes mellitus	ND	ND
173	Type II diabetes mellitus	Type II diabetes mellitus	Type II diabetes mellitus	ND
174	ND	ND	Tyrosine metabolism	ND
175	ND	Ubiquitin mediated proteolysis	Ubiquitin mediated proteolysis	ND
176	ND	ND	ND	Vascular smooth muscle contraction
177	ND	Vasopressin-regulated water reabsorption	Vasopressin-regulated water reabsorption	Vasopressin-regulated water reabsorption
178	VEGF signaling pathway	VEGF signaling pathway	VEGF signaling pathway	VEGF signaling pathway
179	Vibrio cholerae infection	ND	Vibrio cholerae infection	ND
180	Viral carcinogenesis	Viral carcinogenesis	Viral carcinogenesis	Viral carcinogenesis
181	ND	Viral myocarditis	Viral myocarditis	ND
182	Wnt signaling pathway	Wnt signaling pathway	Wnt signaling pathway	Wnt signaling pathway
